# The mechanism by which enoxaparin sodium–high-viscosity bone cement reduces thrombosis by regulating CD40 expression in endothelial cells

**DOI:** 10.1186/s12891-022-05469-5

**Published:** 2022-05-30

**Authors:** Linchao Sang, Kangning Hao, Luobin Ding, Xiaoyu Shen, Hui Sun, Dehao Fu, Xiangbei Qi

**Affiliations:** 1 Department of Orthopaedic Surgery, The Third Hospital of Hebei Medical University, Shijiazhuang, China; 2 Department of Orthopaedic Surgery, Faculty of Medicine, Oita University, Oita, Japan; 3 Department of Orthopedics, Shanghai General Hospital, Shanghai Jiao Tong University School of Medicine, Shanghai, PR China

**Keywords:** Thrombosis, Enoxaparin sodium, Bone cement, CD40, Endothelial cells

## Abstract

**Objective:**

PMMA bone cement leads to the development of local thrombi. Our study found that ES-PMMA bone cement, a novel material, can reduce local thrombosis. We used a simple and reproducible animal model to confirm the reduction in local thrombosis and preliminarily explored the associated molecular mechanism.

**Methods:**

New Zealand rabbits, which were used to model thrombosis using extracorporeal carotid artery shunts, were divided into the following three groups, with 10 rabbits in each group: the sham group, PMMA group and ES-PMMA group. Four hours after modelling, experimental samples were collected, and the degree of thrombosis was compared between the groups. The expression of thrombomodulin in endothelial cells was quantified in vascular tissues samples.

**Results:**

Thrombosis was observed in the PMMA group and ES-PMMA group but not in the sham group. The thrombosis weight was 0.00732 ± 0.00089 g/cm in the PMMA group and 0.00554 ± 0.00077 g/cm in the ES-PMMA group (*P* < 0.001). Quantitative real-time polymerase chain reaction (RT–qPCR) and Western blotting revealed that the expression of CD40, which can regulate thrombosis in vascular endothelial cells, was significantly lower in the ES-PMMA group than in the PMMA group.

**Conclusion:**

Compared with PMMA bone cement, ES-PMMA bone cement can reduce local thrombosis by decreasing the expression of the thrombus-associated regulatory protein CD40 in vascular endothelial cells.

**Supplementary Information:**

The online version contains supplementary material available at 10.1186/s12891-022-05469-5.

## Background

Bone cement has been used for a long time in the clinic, and with the continuous development of joint replacement strategies, operation-related complications and adverse events have gradually emerged. The incidence of bone cement implantation syndrome (BCIS), one of the important and severe complications, is as high as 28% [1]. However, the exact mechanism of BCIS has not yet been fully determined. At present, it is believed that BCIS is mainly caused by allergic reactions, pulmonary embolism, complement activation and histamine release combined with an increase in pulmonary vascular resistance and ventilation/perfusion mismatch, eventually leading to acute hypoxia, right ventricular failure and cardiogenic shock [2]. Although pulmonary embolism is rare after joint replacement, the mortality rate is very high. According to relevant reports, the incidence of pulmonary embolism after joint replacement may be as high as 0.2–0.4% [3].

The current research focus is the toxicity of bone cement particles in the blood, as thrombosis results from the activation of the human complement system and the human coagulation system due to thermal effects [4]. Thrombus shedding can lead to reductions in extremity venous thrombosis or pulmonary embolism, but whether the physical and chemical properties of bone cement can directly lead to local thrombosis has not been studied.

Enoxaparin sodium is an anticoagulant drug available as an injection or powder that is widely used for the prevention and treatment of thrombosis-related diseases in orthopaedic patients [5]. Enoxaparin sodium high-viscosity bone cement is a biomaterial made by mixing enoxaparin sodium powder with bone cement powder in a certain proportion and then mixing the product with liquid [6].

This study shows that high-viscosity bone cement can induce local thrombosis in animal models; confirms that compared with ordinary high-viscosity bone cement, enoxaparin sodium high-viscosity bone cement, a novel material, can reduce local thrombosis; and elucidates the preliminary molecular mechanism by which this material reduces local thrombosis, as described in detail below.

## Material and method

### Animal model, grouping and experimental reagents

**Fig. 1:**
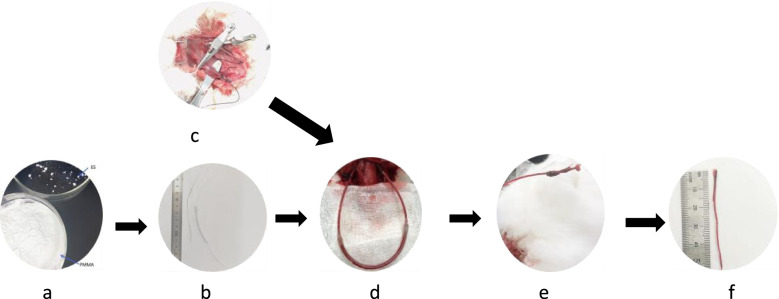
**a** Enoxaparin sodium (ES) and high-viscosity bone cement powder samples. **b** The extracorporeal shunt and prepared silk thread with bone cement. **c** The exposed arteriovenous vessels of New Zealand rabbits. **d** Establishment of the animal model. **e** Blood vessels contacting thread covered with bone cement. f: Intravascular bone cement-induced thrombus samples (the original images are presented in Supplementary Fig. 1)

**Fig. 2:**
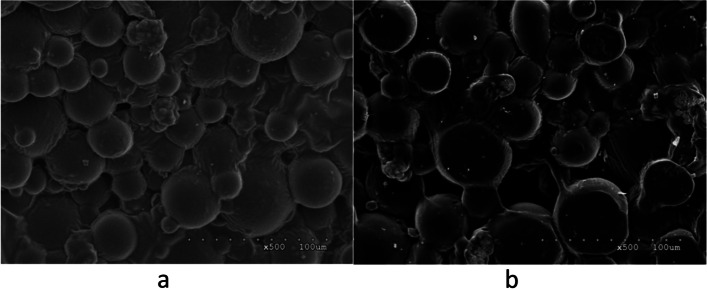
**a** PMMA. There were many gaps between the bone cement particles. **b** ES-PMMA. The surface of the bone cement particles was covered with a syrup-like substance, which was considered the attached enoxaparin (the original images are presented in Supplementary Fig. 2)

Six-month-old male New Zealand rabbits (Wangdu Tonghui Animal Breeding Co., Ltd., animal certificate No.: 210426) (protocol approved by the Medical Ethics Committee of the Third Medical College of Hebei Medical University, ethics acceptance No. z2021–007-2) weighing 2.5 ± 0.5 kg were anaesthetized. An appropriate depth of anaesthesia was maintained with 20% urethane. The rabbits were placed in the supine position, and an extracorporeal carotid artery shunt was placed as described in the literature [7]. The rabbits were divided into three groups: the sham-operated group (FS group) (*n* = 10), ordinary high-viscosity polymethylmethacrylate (PMMA) bone cement group (Con group) (*n* = 10) and novel enoxaparin sodium high-viscosity polymethylmethacrylate (ES-PMMA) bone cement group (M group) (*n* = 10). To generate ES-PMMA, 8000 AXa IU enoxaparin sodium powder (Chengdu Baiyu, China) was premixed with 40 g PMMA bone cement (Heraeus, Germany) [6] followed by liquid. Then, the mixture was placed under a high-power scanning electron microscope (SEM, Hebei Medical University Electron Microscopy Center, Hitachi, S-3500 N), and the characteristics of the two types of bone cement were compared. The bone cement was used to glue surgical silk thread in the different groups, and we ensured that the bone cement completely and evenly covered the thread. Then, the bone cement was inserted into the shunt and extended approximately 1 cm into the blood vessel through the shunt so that it made contact with the vascular endothelium. The bone cement was maintained in the circulation for 4 hours. Rabbits in the FS group underwent the same procedure as those in the other two groups except nothing was placed in the shunt. The procedures used for modelling and samples collected are shown in Fig. 1, and the characteristics of PMMA and ES-PMMA are shown in Fig. 2.

### Sample collection

Four hours after modelling, we collected and analysed samples. Bone cement-induced thrombus samples were obtained from the Con group and M group, and the degree of thrombus attached to the thread covered with bone cement was determined. Vascular tissue sample that contacted the bone cement were stored in a − 80 °C freezer and processed in the same manner as those from the FS group. The expression of thrombus-related regulatory proteins in endothelial cells in vascular tissue cells was determined by quantitative real-time polymerase chain reaction (RT–qPCR), Western blotting and immunofluorescence.

### RT–qPCR analysis of the mRNA expression of thrombus-associated proteins in endothelial cells in vascular tissue

Total RNA was extracted from tissues from the different groups with TRIzol reagent (Invitrogen, USA) according to the manufacturer’s instructions. The mRNA was reverse transcribed with a PrimeScript RT Reagent Kit (Takara, Japan). A SYBR Premix Ex Taq Kit (Servicebio, Wuhan) was used for RT–qPCR on an iQ5 Real-Time PCR instrument (Applied Biosystems USA). The reaction conditions were as follows: 94 °C for 4 min, 94 °C for 30 s, 60 °C for 30 s and 72 °C for 30 s for a total of 40 cycles. Each sample was analysed three times. The primer sequences for the polymerase chain reaction (PCR) were as follows: CD31: F, TCCTACGATGCCAGGTCTGA, and R, CATTTCGGCATGGGAATGGC; CD40: F, GGCGGGAACTAACAAGACAG, and R, GCGGTAGCCCTTATCTATTGG; CD62p: F, AGTGTGTAGCTGTCCAGTGC, and R, AGTCACCAAAGGGATGCGAG; CD106: F, GCCCTTTGGAGGTTGGAGAA, and R, GAACTGGTAGACCCTCGCTG; endothelin: F, TGACTCCCAGAGAGGACGTG, and R, CTCCTGGACGGCTACAATCC; thrombomodulin: F, TTCCTCTGCGAGTTCCCCTT, and R, CGTAACAGGTCAGCTCCAAG; and GAPDH: F, TGGAATCCACTGGCGTCTTC, and R, TCATGAGCCCCTCCACAATG. The 2^-ΔΔCT^ method was used for quantitative analysis of relative mRNA expression, and a histogram was drawn. The expression of each gene was normalized to the expression of GAPDH.

#### Western blot analysis

Blood vessel samples from the different groups were washed with ice-cold PBS and then lysed with RIPA buffer (Beyotime, China) containing protease inhibitor. The protein samples were separated on SDS–PAGE gels of different percentages and then transferred to a polyvinylidene fluoride (PVDF) membrane (Millipore, USA). The membrane was incubated with primary antibodies against CD31 (Abcam, UK), CD40 (Abcam, UK), CD62P (Abcam, UK), CD106 (Abcam, UK), endothelin (Abcam, UK) and thrombomodulin (Abcam, UK) at 4 °C with shaking overnight and then with a corresponding secondary antibody for 1.5 hours. Western LightningTM Chemiluminescence Reagent was used to develop the blot for 30 s, and then the membrane was immediately placed in an exposure box and exposed for 1 min in a darkroom. The membrane was imaged and analysed with a LabWorksTM gel imaging and analysis system (UVP, USA). GAPDH was used as an internal control.

#### Tissue immunofluorescence

Frozen vascular tissues from the different groups were allowed to stand for 1 hour and then sliced at a thickness of 8 μm. The tissues were placed in Triton X-100 (mass fraction 0.5%) for 10 min, blocked with 4% goat serum albumin and incubated with an anti-cd40 primary antibody (Abcam, UK) diluted 1:200 overnight at 4 °C. Then, the tissues rinsed with PBS 3 times, incubated with anti-sheep and anti-rabbit IgG secondary antibodies (Abcam, UK) diluted 1:400, rinsed with PBS at room temperature for 2 hours, dried, observed and photographed under a fluorescence microscope. Image-Pro Plus software was used for semiquantitative analysis of the images, and the fluorescence intensity was calculated.

### Data analysis

All experiments were carried out three times. The results are expressed as the mean ± standard deviation. T test was used for comparisons between groups. Statistical analysis was performed using SPSS software (version 17.0). *P* ≤ 0.05 was considered statistically significant. Significant is indicated as follows: *, P ≤ 0.05; **, *P* ≤ 0.01; and ***, *P* ≤ 0.001.

## Result

### Characterization of PMMA and ES-PMMA by electron microscopy

Animals in the M group were treated with 8000 AXa IU enoxaparin sodium mixed with 40 g PMMA powder. Under an electron microscope, it was observed that the gaps between the particles on the surface of ES-PMMA were filled with a syrup-like substance, which we considered the attached enoxaparin.

### Analysis of thrombus in different samples

Four hours after the establishment of the animal model, the thrombosis weight was 0.00732 ± 0.00089 g/cm in the Con group and 0.00554 ± 0.00077 g/cm that in the M group (*P* < 0.001). The difference in thrombosis weight between the groups was significant. No thrombosis was observed in the FS group in our study (Supplementary Table 1).

### Differential expression of thrombus-associated proteins in endothelial cells in vascular tissue

**Fig. 3:**
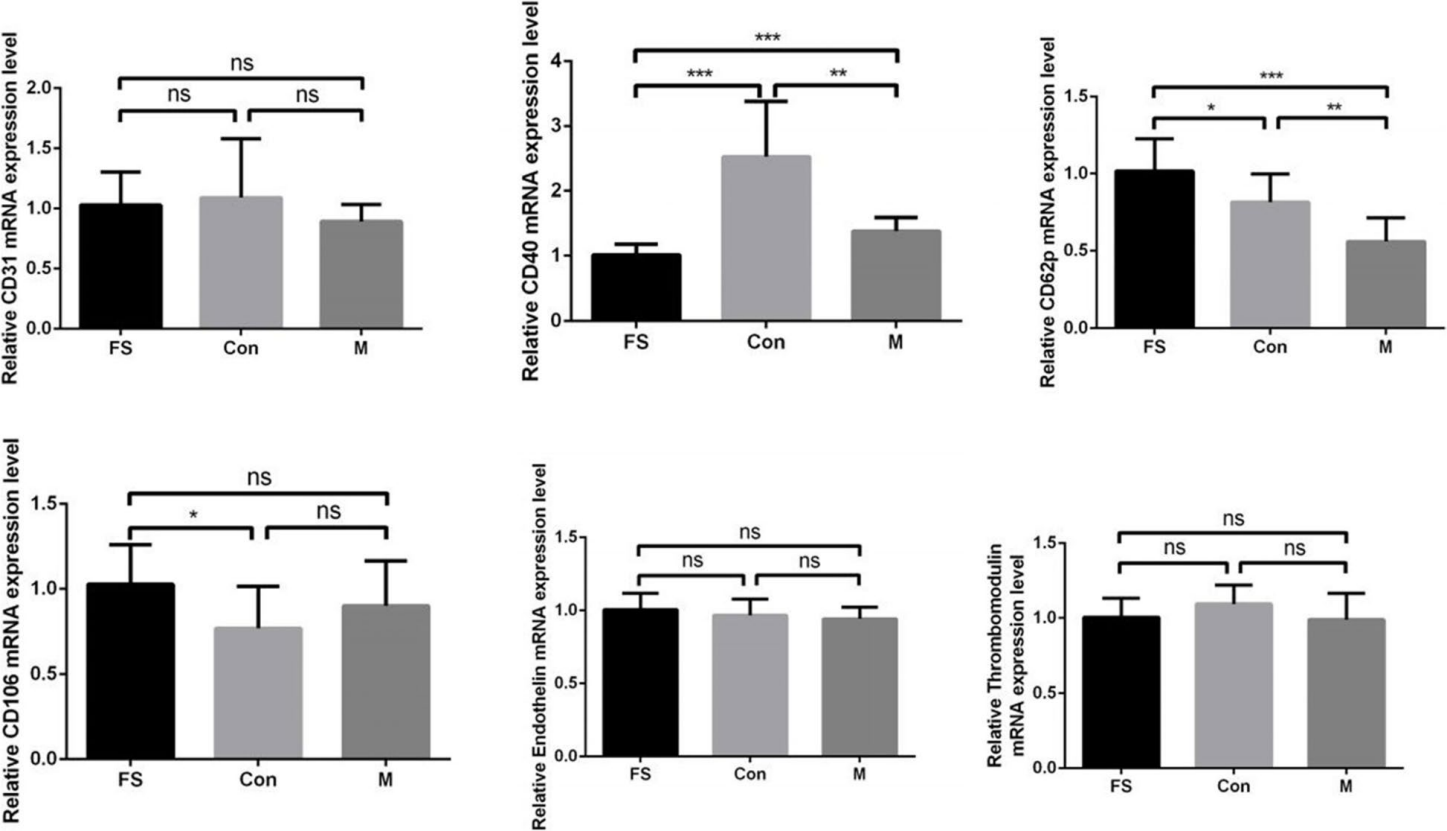
mRNA expression levels of CD31, CD40, CD62p, CD106, endothelin, and thrombomodulin showing that CD62p and CD40 expression was decreased in the M group compared with the Con group (*P* < 0.01) and that CD40 mRNA expression was significantly decreased in the FS group compared with the Con group (*P* < 0.001. (the raw data and image are presented in Supplementary Fig. 3)

Vascular endothelial cells from the Con group and M group, which contacted the bone cement, were collected for RT–qPCR, Western blotting and immunofluorescence. Vascular endothelial cells from the FS group were collected for the same analyses. Relevant studies have shown that thrombosis is related to inflammation, a hypercoagulable state, vascular endothelial cell injury or functional regulation. Proteins related to thrombosis, mainly are CD62p, CD40, CD31, endothelin, thrombomodulin and CD106, are expressed by endothelial cells. We measured the mRNA expression levels of six thrombus-related proteins in the samples by RT–qPCR. The results are shown in Fig. 3. We found that the expression of CD62p and CD40 was significantly decreased in the M group compared with the Con group (*P* < 0.01) and that the expression of CD40 was significantly decreased in the FS group compared with the Con group (*P* < 0.001). There was no significant difference in other indexes between the Con groups and M group (*P* > 0.05).

**Fig. 4:**
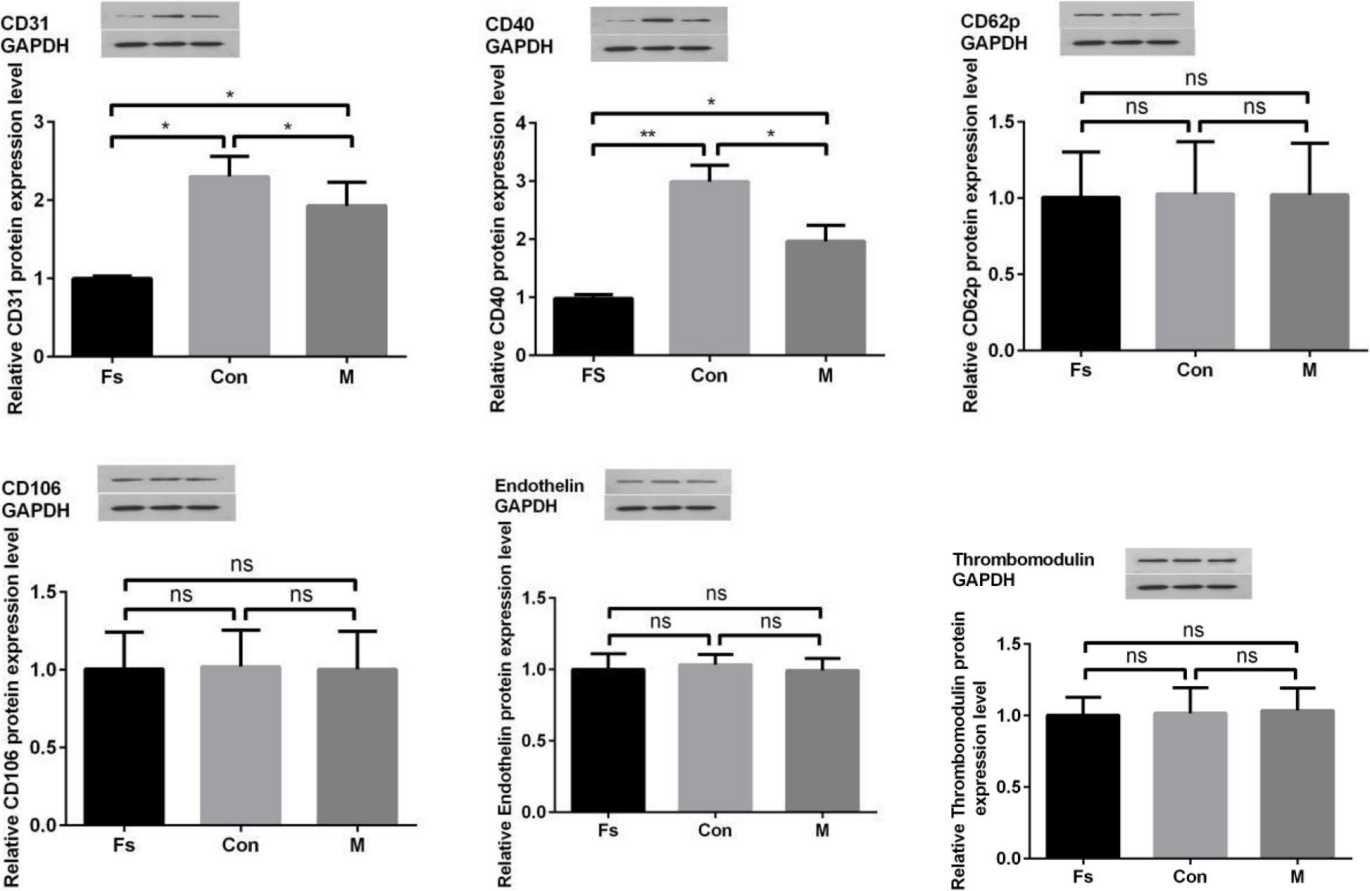
Protein expression levels of CD31, CD40, CD62p, CD106, endothelin, and thrombomodulin. The expression levels of CD31 and CD40 were decreased in the M group compared with the Con group (*P* < 0.05), and the expression levels of CD40 were significantly decreased in the FS group compared with the Con group (*p* < 0.01) (the original images were cropped to improve the clarity and conciseness of the presentation; the original blots/gels are presented in Supplementary Fig. 4)

The expression of these six proteins was assessed by Western blotting. As shown in Fig. 4, the protein expression of CD31 was decreased in the M group compared with the Con group (*P* < 0.05), and the protein expression of CD40 was significantly decreased in the M group compared with the Con group (*P* < 0.01). There was no significant difference in other indexes between the two groups.

### Fluorescence staining of CD40 for identification of vascular endothelial cells

**Fig. 5:**
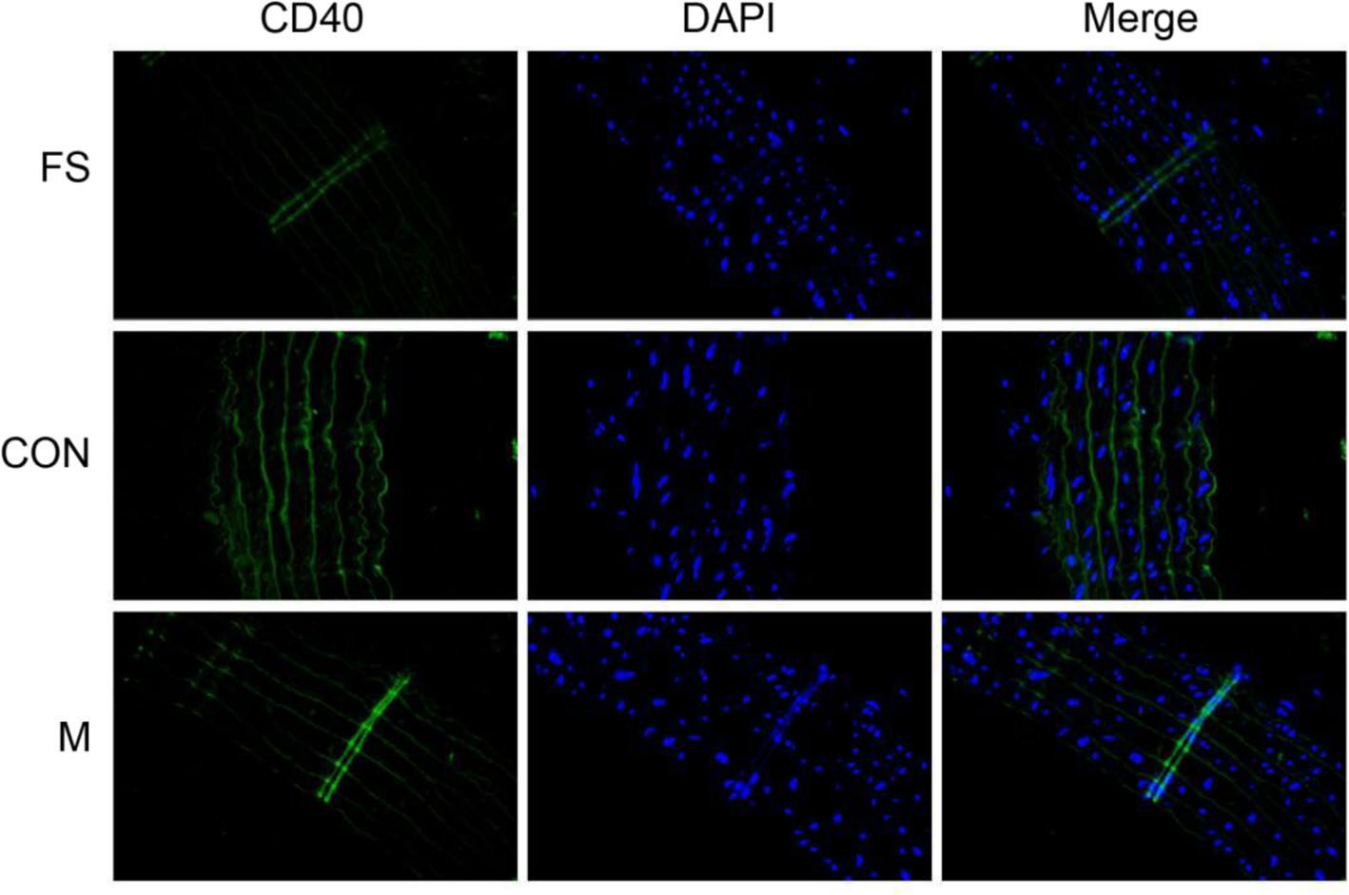
The fluorescence intensity of CD40 in vascular endothelial cells was significantly lower in the M group and FS group than in the Con group (the original images are presented in Supplementary Fig. 5)

Given that CD40 mRNA and protein expression was significantly lower in the M group than in the Con group, immunofluorescence staining of CD40 in the three groups was performed. The results are shown in Fig. 5; base magnification: 200 × .

## Discussion

The application of high-viscosity bone cement in clinical practice is accompanied by BCIS [2, 8], which has an incidence rate as high as 28%. One of the causes of BCIS is embolism caused by bone cement implantation, including pulmonary embolism and lower limb venous embolism [9]. However, whether the physical and chemical properties of bone cement itself can cause local thrombosis to induce lower limb venous thrombosis or pulmonary embolism has not been reported.

We simulated the local surgical environment and studied whether bone cement could cause the development of thrombosis around the bone cement through quantitative analysis. Since bone cement is dispersed in the bodies of animals after the establishment of animal models of hip and knee replacement and it is very difficult to collect tissue samples around bone cement, we simulated the bone cement microenvironment during surgery. During joint replacement, the arteriovenous and small blood vessels around the joint that is removed during the operation, the small blood vessels in the medullary cavity and the blood circulating in the blood vessels contact bone cement. The main reasons for thrombosis include a slow endovascular blood stream, high blood condensation, platelet activation, endothelial cell injury or abnormal expression of thrombosis-related regulatory proteins. Due to its high viscosity, bone cement can affect the blood vessels and the composition of the blood flowing through them. We established a New Zealand rabbit model of extracorporeal carotid artery shunt and then implanted a silk thread covered in bone cement to simulate the microenvironment in which blood flows through the surface of bone cement so that it can contact the inner wall of blood vessels to assess its influence on tiny blood vessels. By using this model, we were able to quantitatively evaluate whether the physical and chemical properties of high-viscosity bone cement can cause the development of local thrombosis.

Our group has developed a novel material by mixing PMMA with ES and determined that the best ratio [6] was 8000 AXa IU ES:40 g PMMA. This bone cement can release heparin sodium to prevent coagulation when the required biomechanical strength is reached.

The reduction in the amount of heparin sodium released from bone cement can theoretically reduce the formation of local thrombosis without causing massive bleeding. We verified whether it can reduce the formation of local thrombosis. In the animal model, we found that thrombosis was attached to three threads covered with high-viscosity bone in the Con group. We also measured the thrombus weight, which was 0.00732 ± 0.00089 g/cm. The thrombus weight in the experimental group was 0.00554 ± 0.00077 g/cm. Although the experimental group exhibited thrombosis, there were significantly fewer thrombi in the presence of ES-PMMA than in the presence of ordinary high-viscosity bone cement.

Thrombosis is mainly caused by blood hypercoagulability, platelet activation, injury of vascular endothelial cells or abnormal expression of clot-related proteins [10–14]. Bone cement causes local thrombosis by directly contacting blood components or acting on vascular endothelial tissue. In this study, vascular tissues in contact with bone cement were selected to compare the influence of ES-PMMA and PMMA on the expression of thrombosis-related proteins in vascular endothelial cells and to illustrate the difference in the influence of these two types of bone cement on thrombosis by measuring the expression of these proteins. Among the many proteins expressed by endothelial cells, endothelin [15], vascular endothelial cell adhesion molecule [16], CD62p [17, 18], CD31 [19], thermoregulatory protein [20, 21], and CD40 [22, 23] are closely related to thrombosis. We assessed the differential expression of thrombus-related proteins in endothelial cells by RT–qPCR and Western blotting and found that the expression of CD40 in the M group was significantly lower than that in the Con group. Immunofluorescence revealed that the fluorescence intensity of CD40 in the M group was lower than that in the Con group. In addition, relevant studies have proven that CD40 in endothelial cells has a regulatory effect on thrombosis [24]. CD40 is a member of the tumour necrosis factor receptor superfamily. The effect of enoxaparin sodium is mainly dependent on by anticoagulant factor Xa [25], which is a serine protease [26]. Serine protease exists in the digestive enzyme and complement system [27, 28]. The complement system is composed of inherent complement components, complement regulatory proteins and complement receptors [29]. Some complement components are expressed in vascular endothelial cells [30], while CD40 is expressed in B cells, mononuclear macrophages, dendritic cells and vascular endothelial cells [31]. Therefore, we found that compared with PMMA bone cement, ES-PMMA bone cement reduced local thrombosis, possibly by regulating vascular endothelial CD40 protein expression.

## Conclusion

In summary, using a simple and reproducible animal model, we proved that PMMA produces local thrombosis and that compared with PMMA, ES-PMMA, a novel material, can reduce local thrombosis A, possibly by regulating low protein expression protein on endothelial cells in vascular tissue.

## Supplementary Information

**Additional file 1.**

## Data Availability

The datasets used and/or analysed during the current study are available from the corresponding author on reasonable request.

## Abbreviations

PMMA: Polymethylmethacrylate
ES: Enoxaparin sodium
SEM: Scanning electron microscopy
