# Supplementary material for: The mechanism by which enoxaparin sodium–high-viscosity bone cement reduces thrombosis by regulating CD40 expression in endothelial cells
Source: BMC Musculoskelet Disord. 2022 May 30;23:513. doi: 10.1186/s12891-022-05469-5 (PMC9150327; doi:10.1186/s12891-022-05469-5)
Supplement: Supplementary file 1 — Additional file 1. [file 12891_2022_5469_MOESM1_ESM.docx]

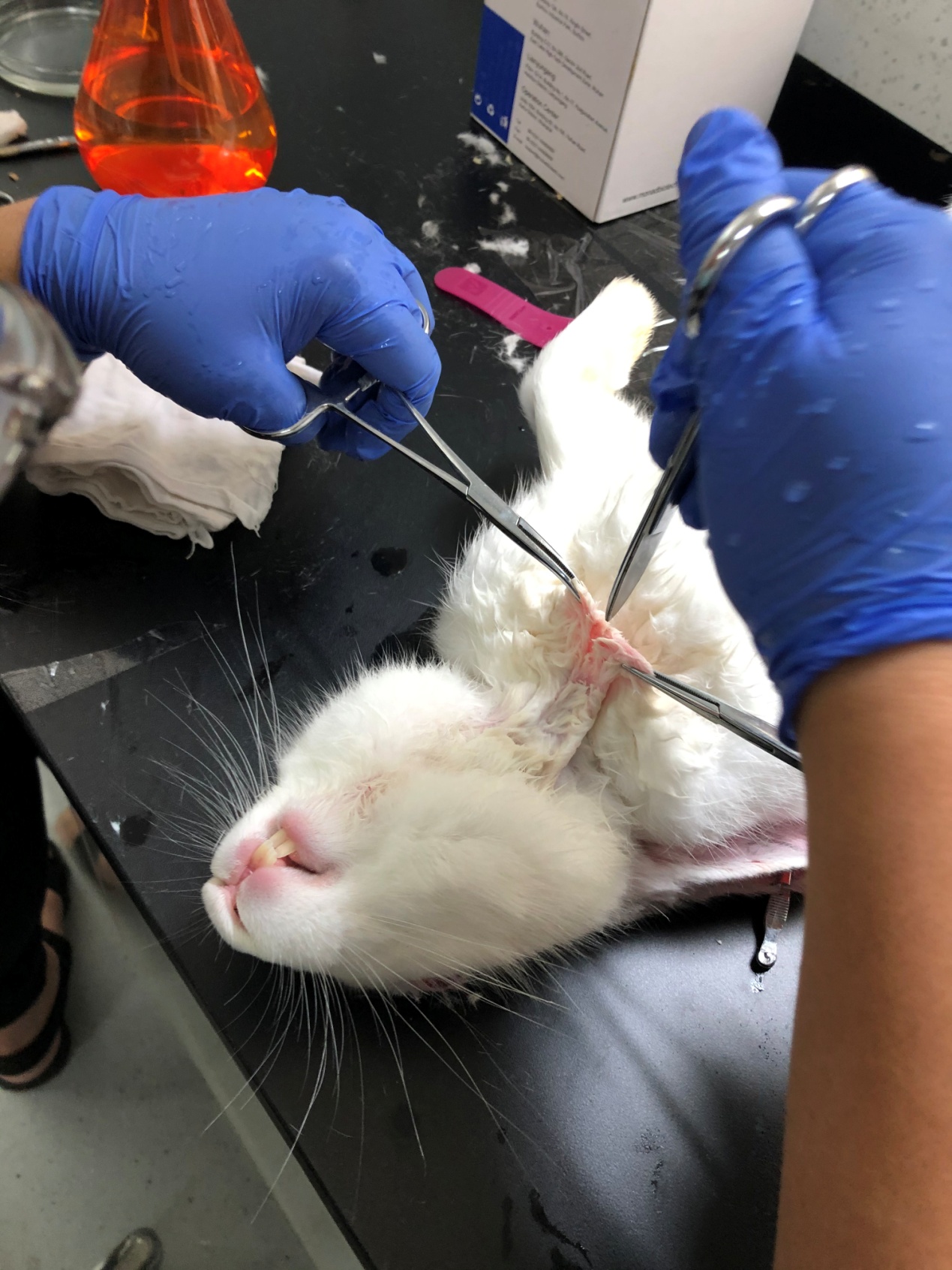

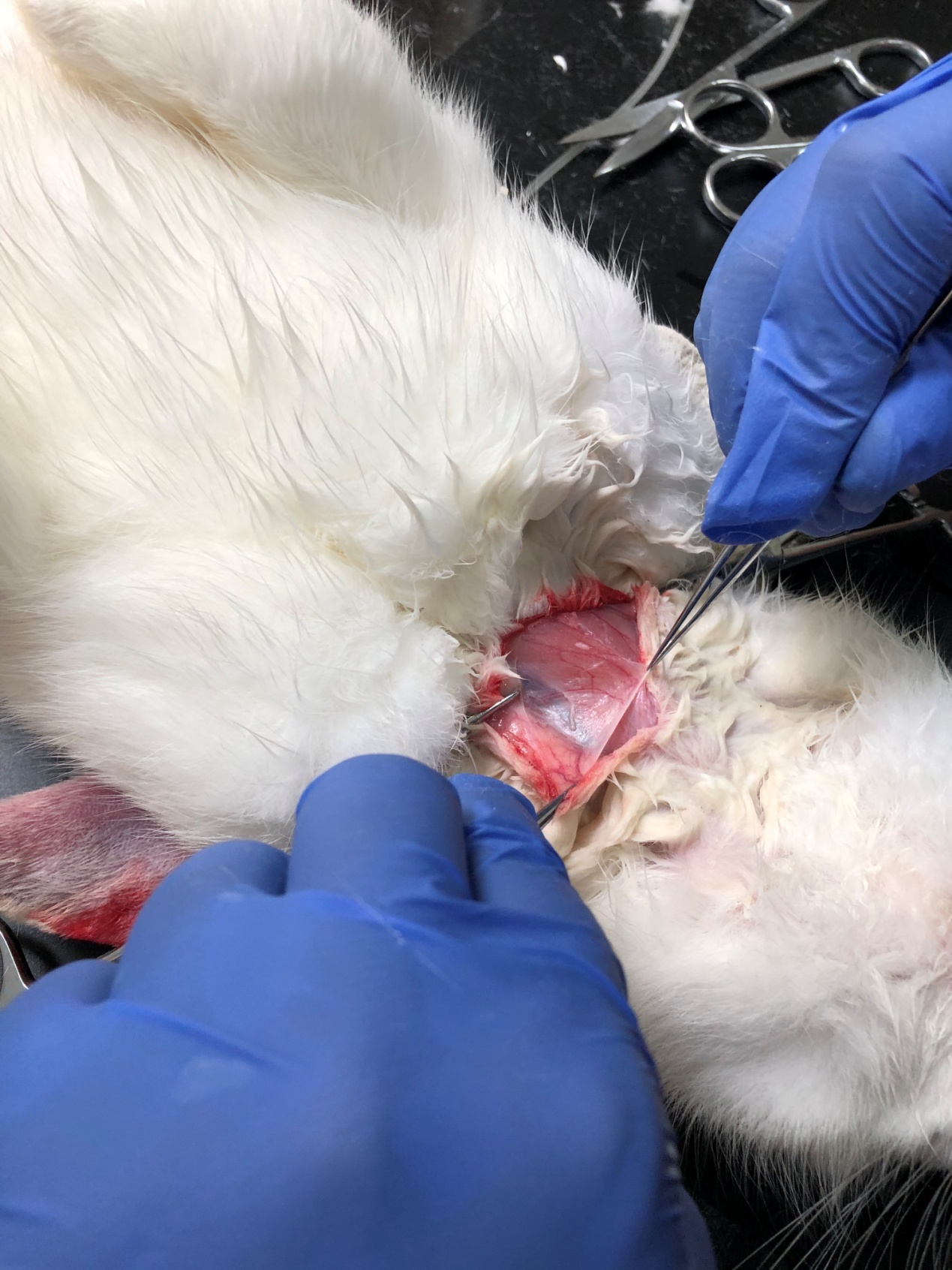

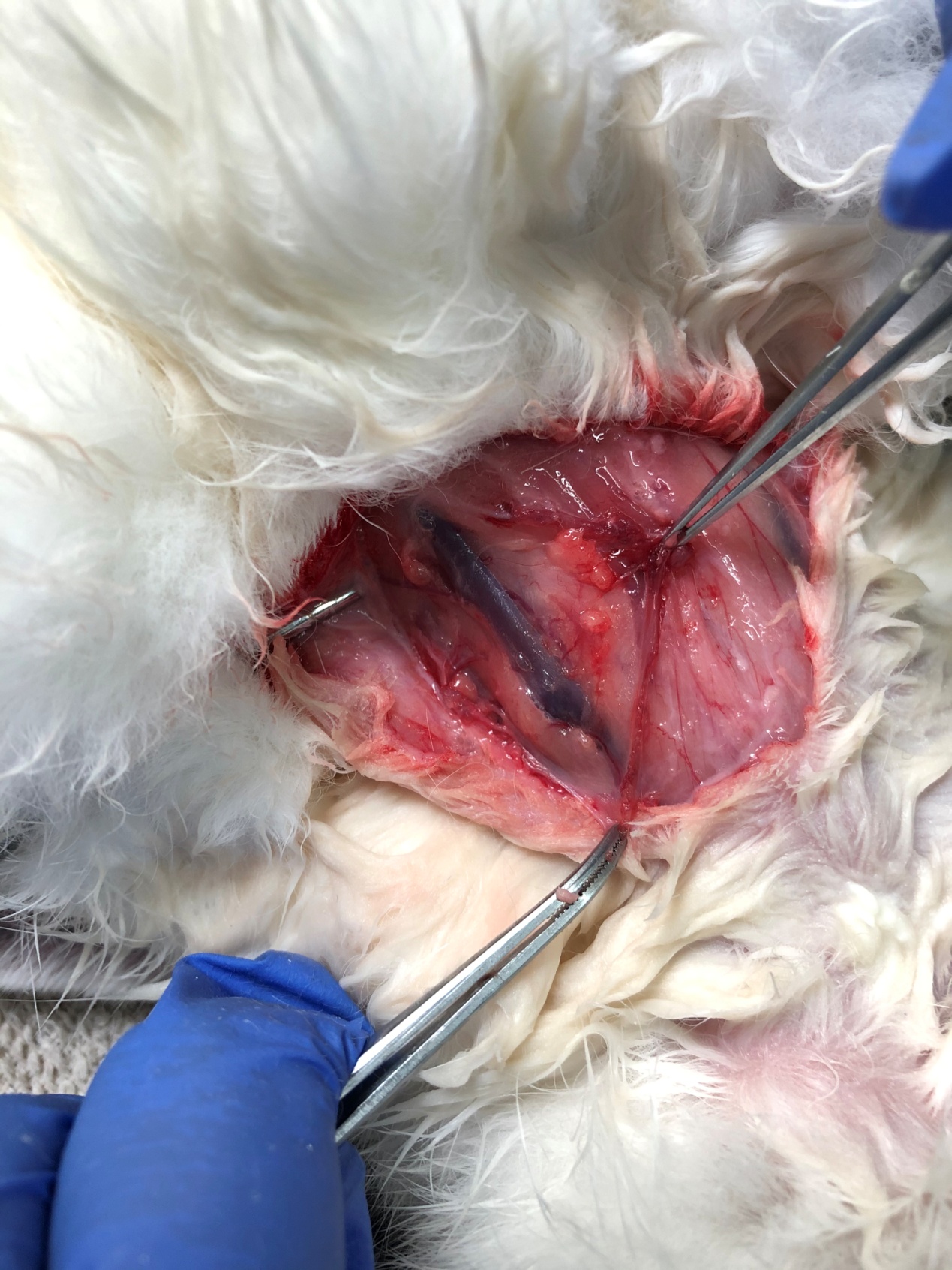

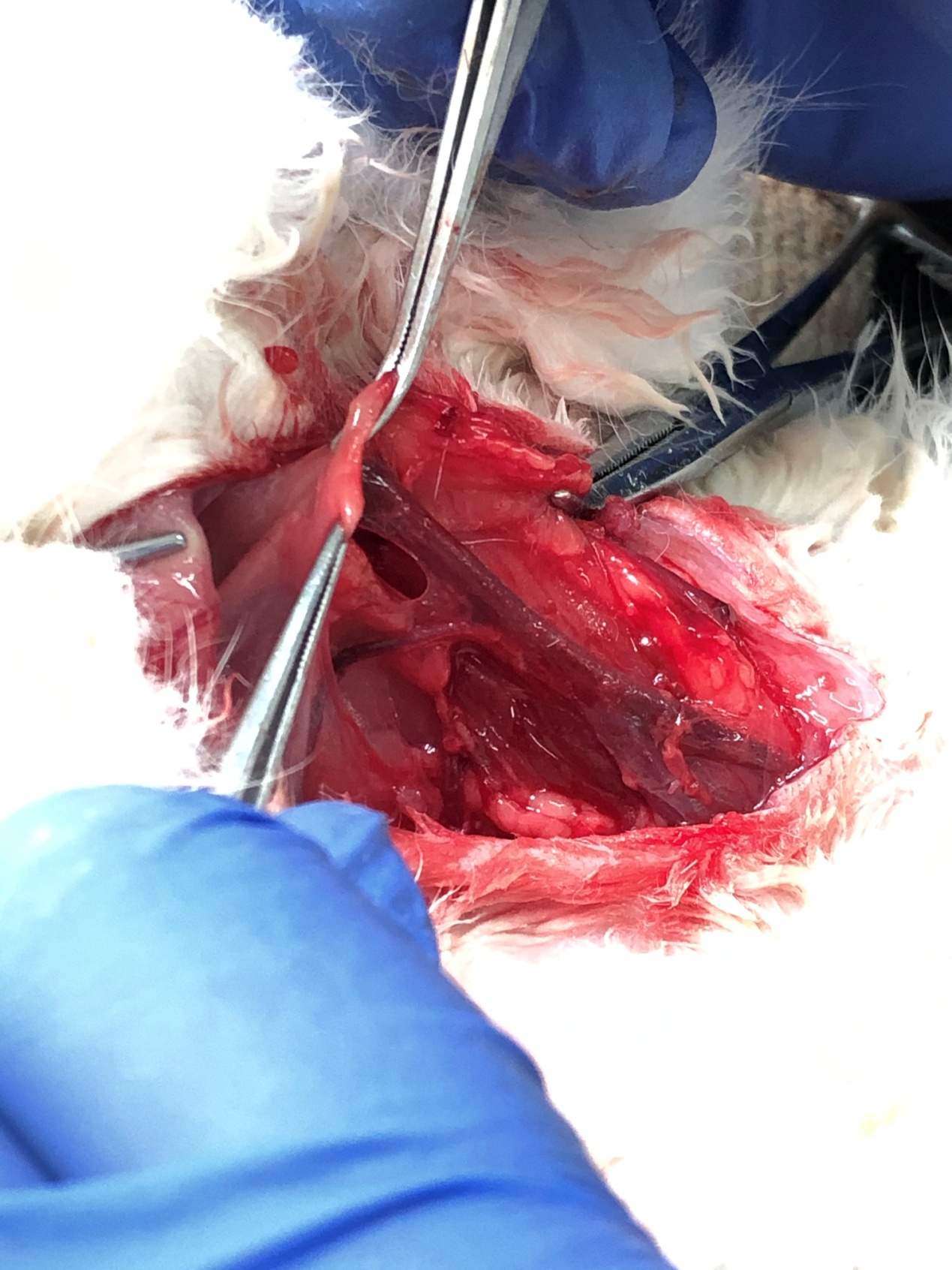

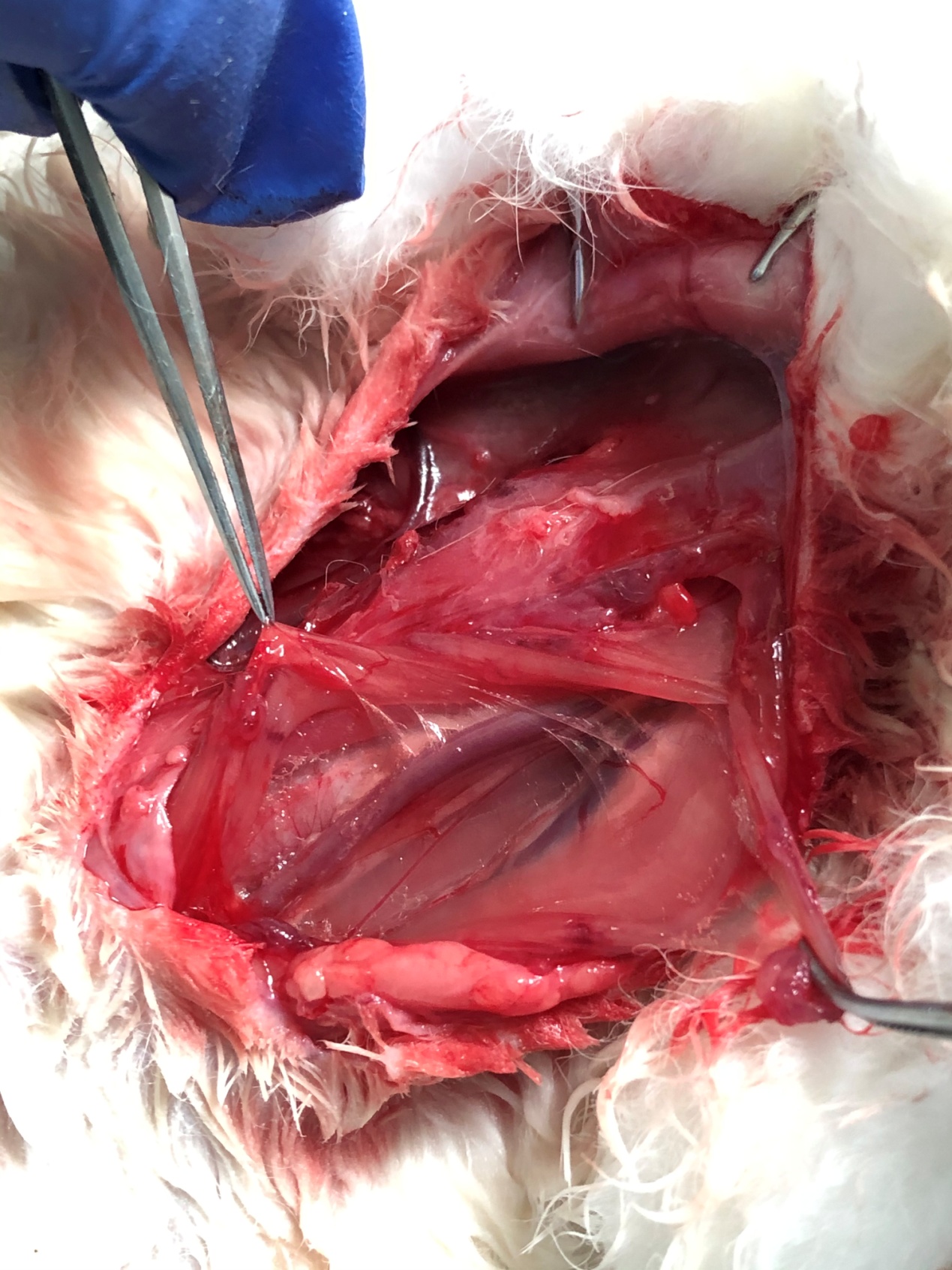

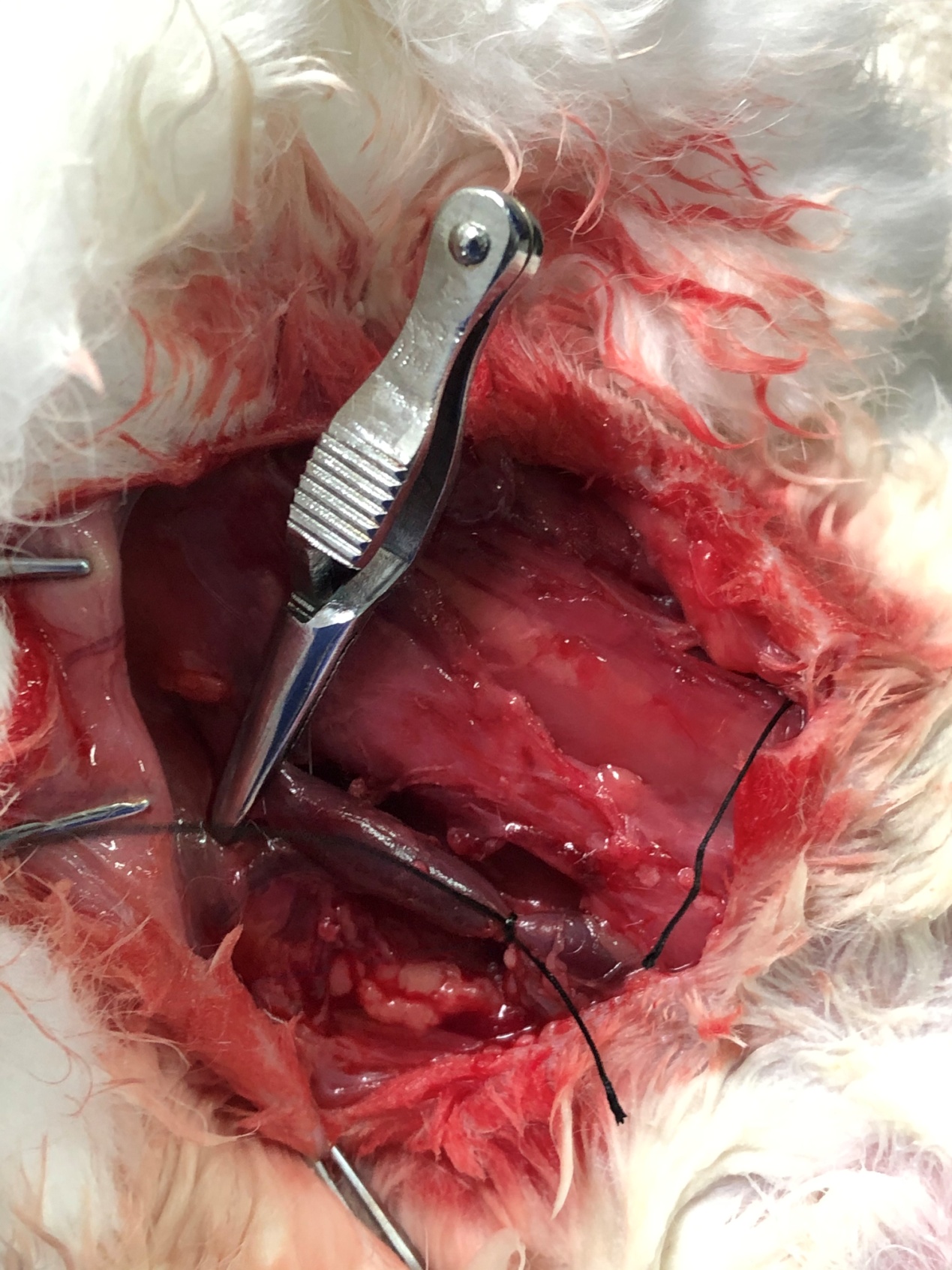

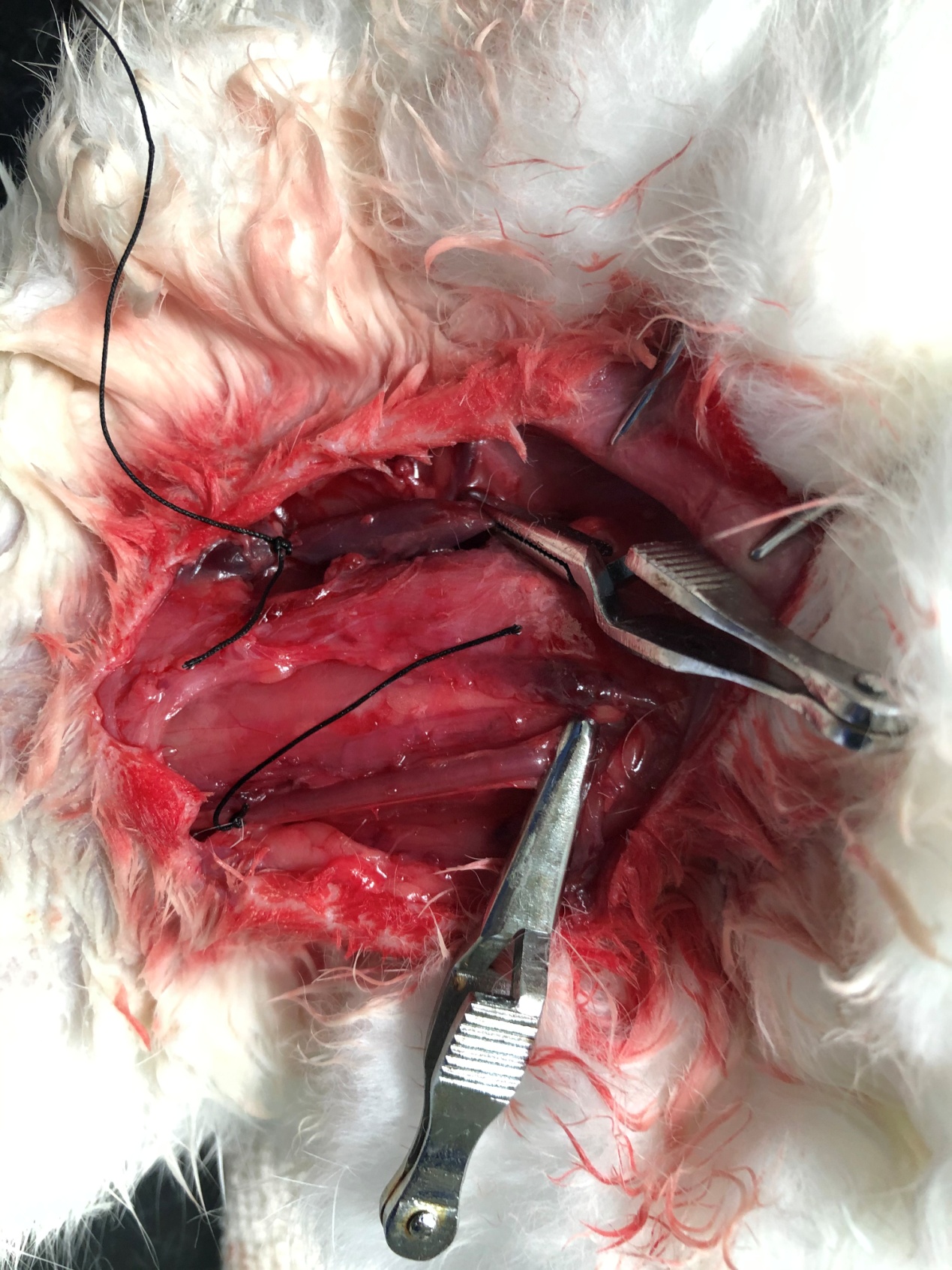

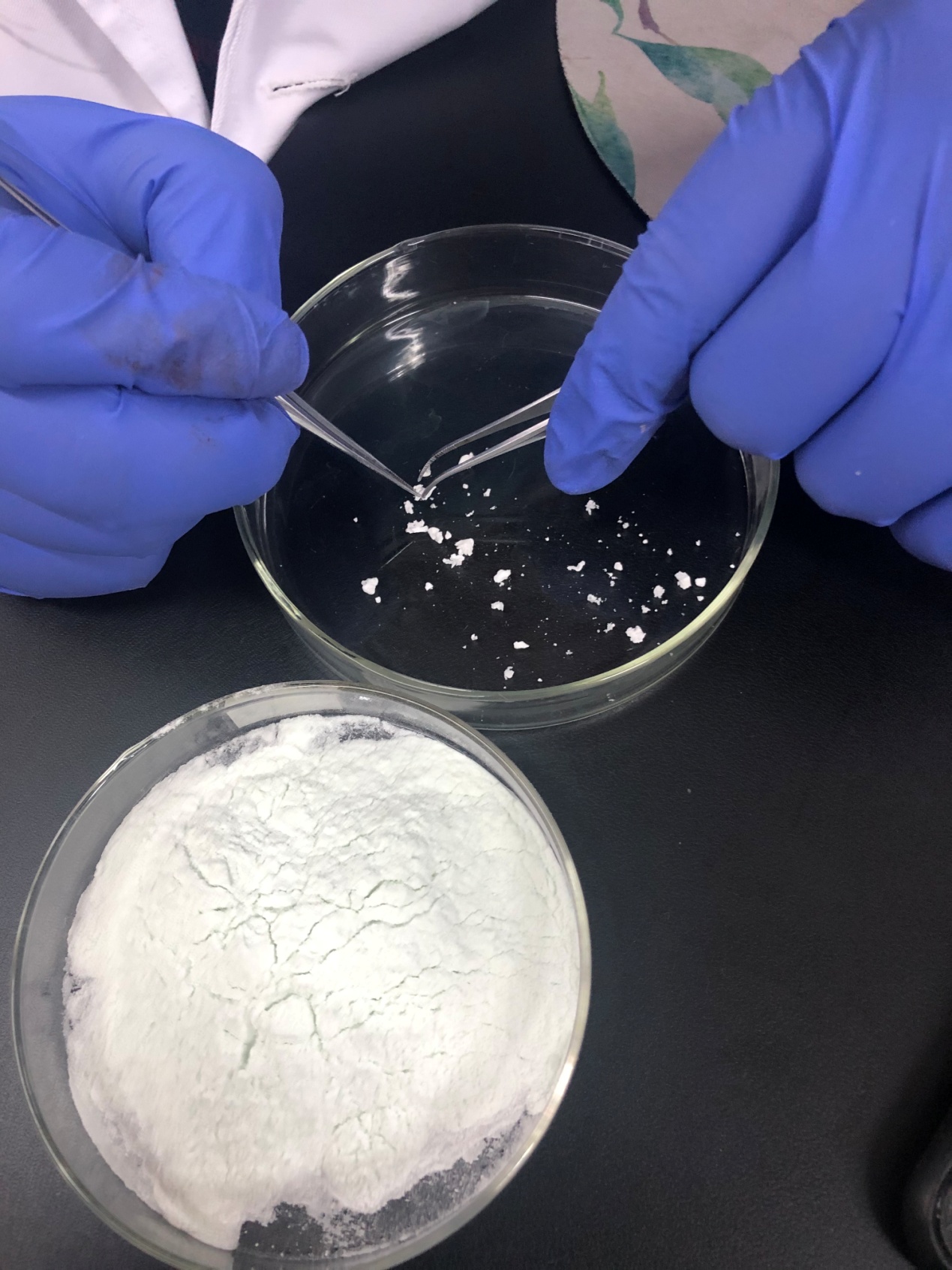

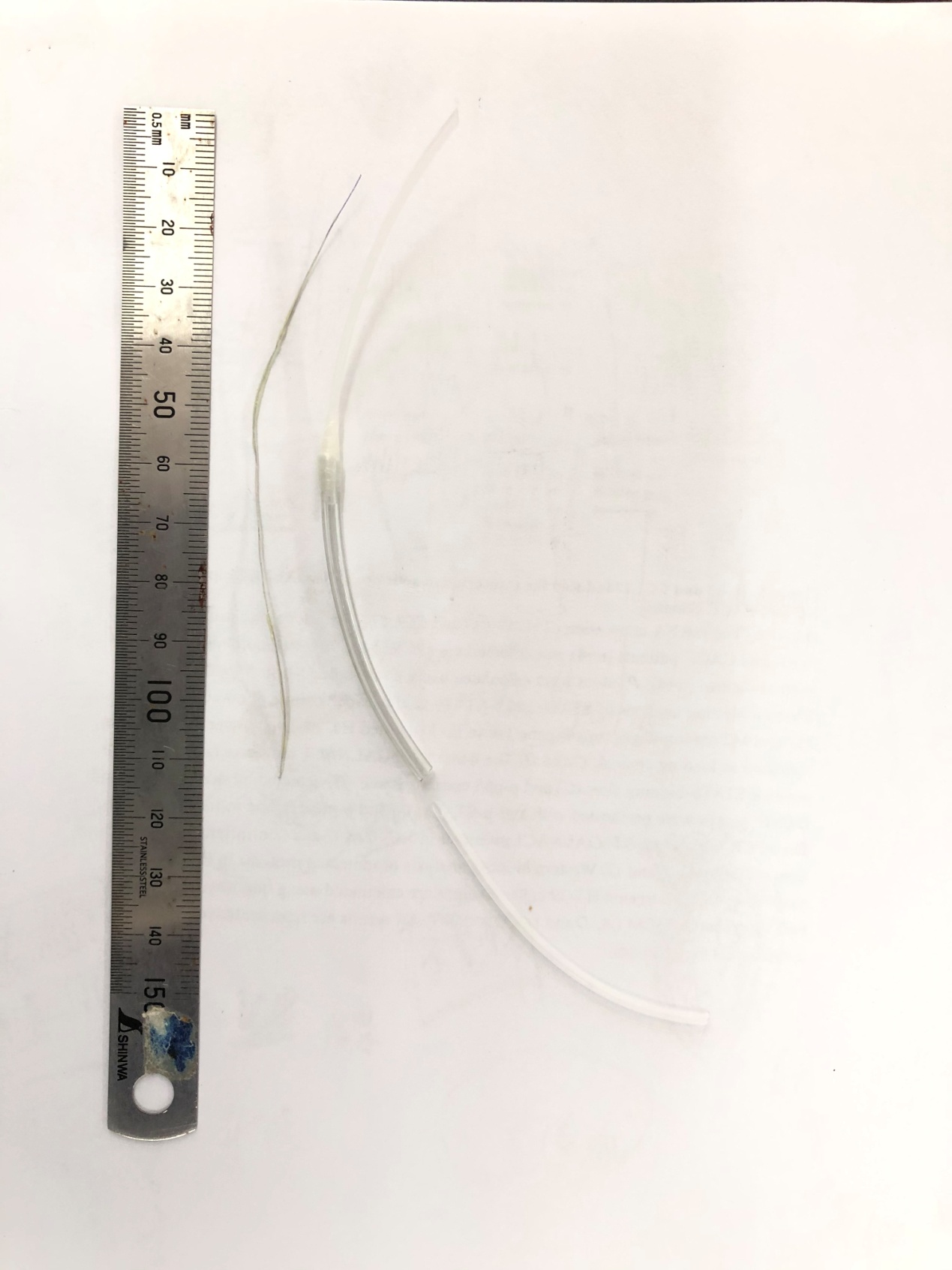

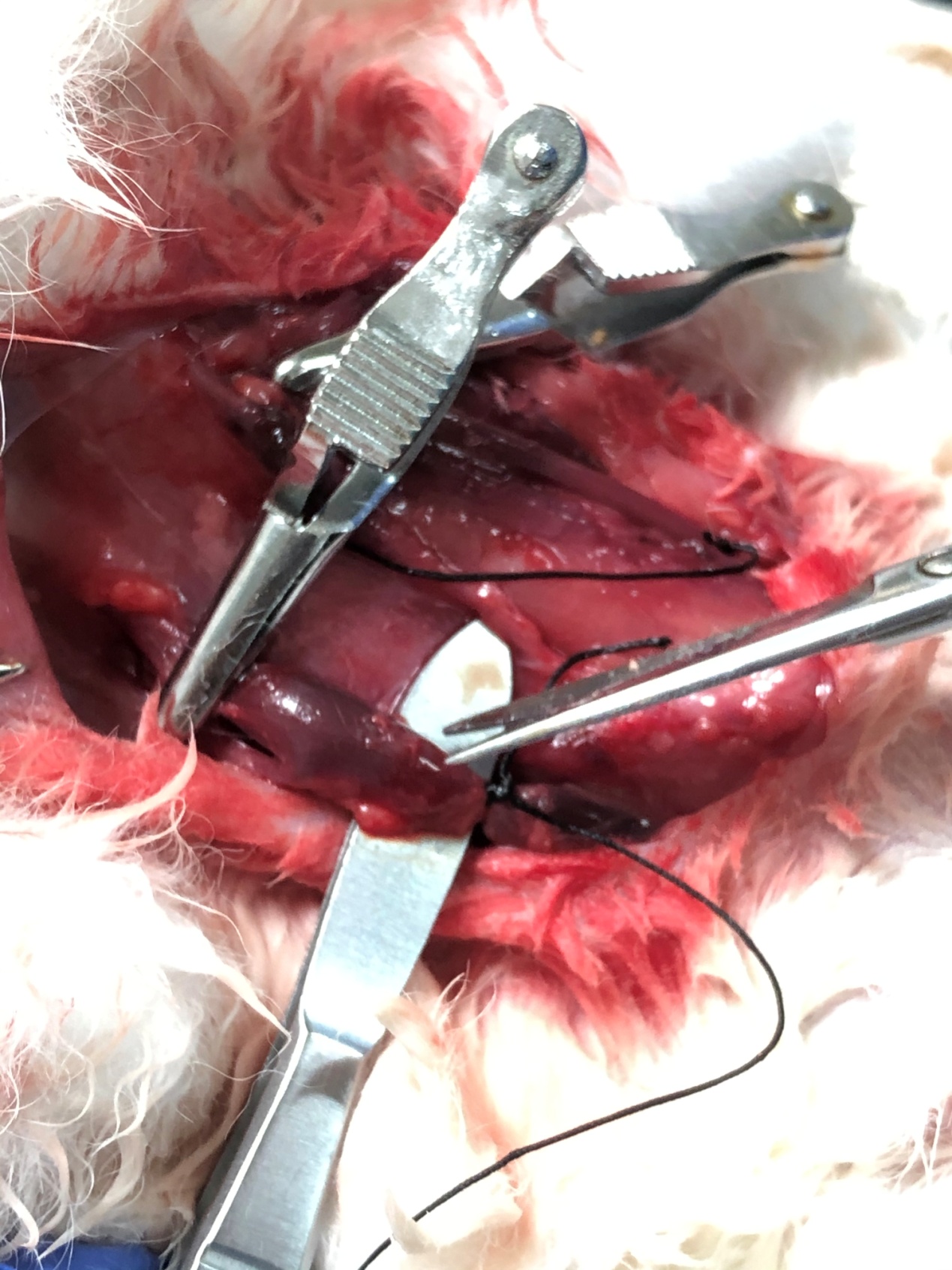

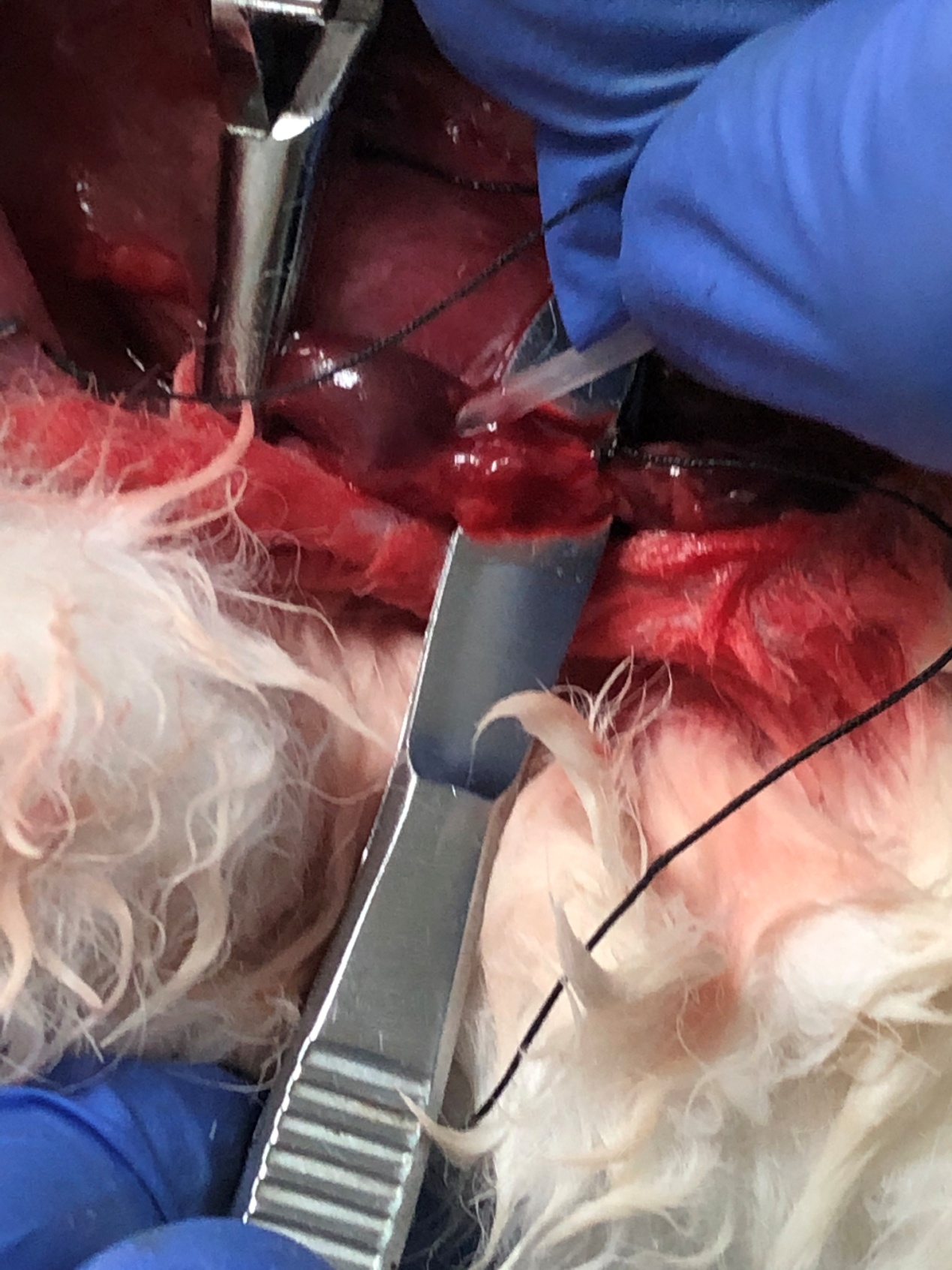

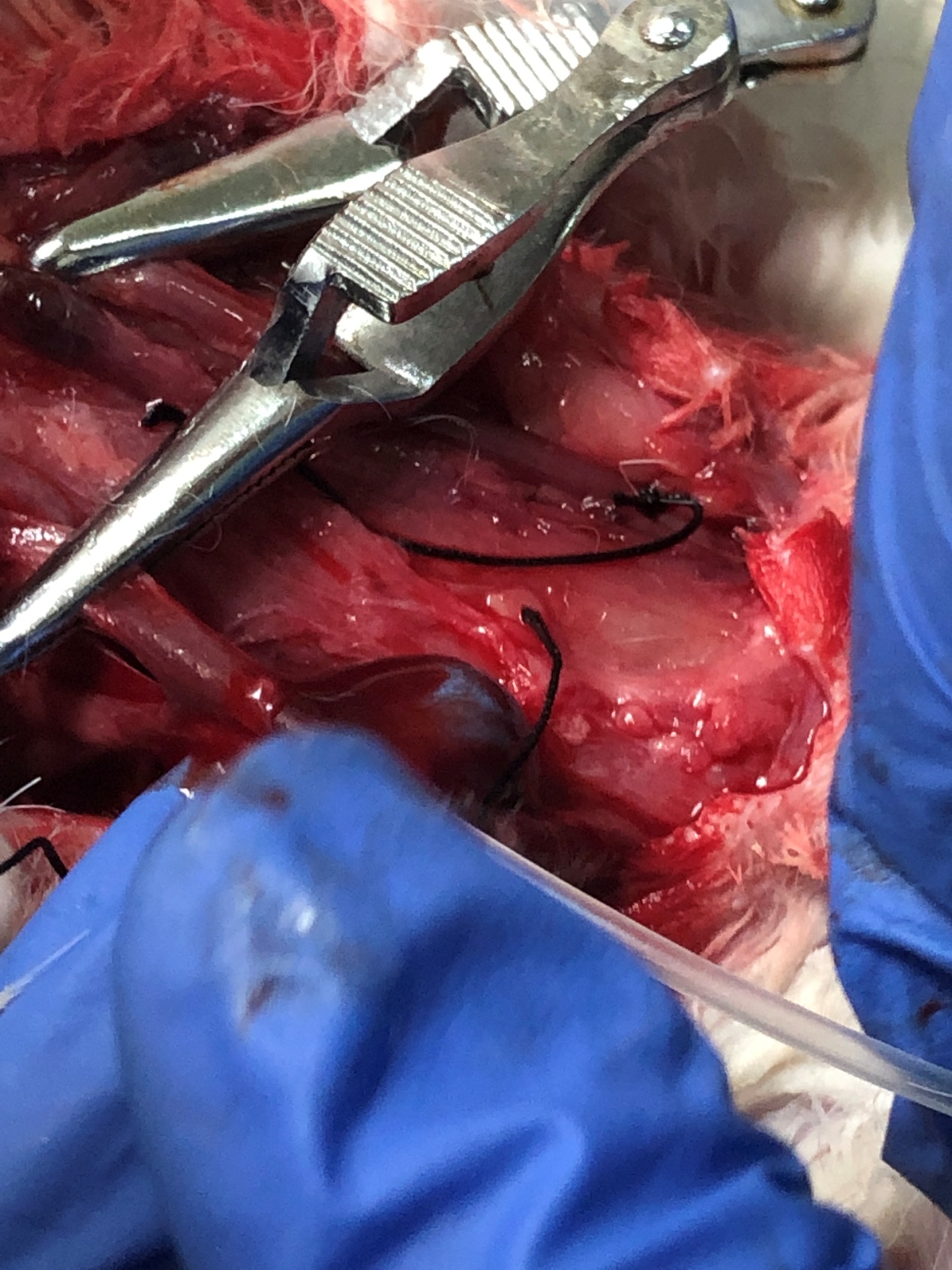

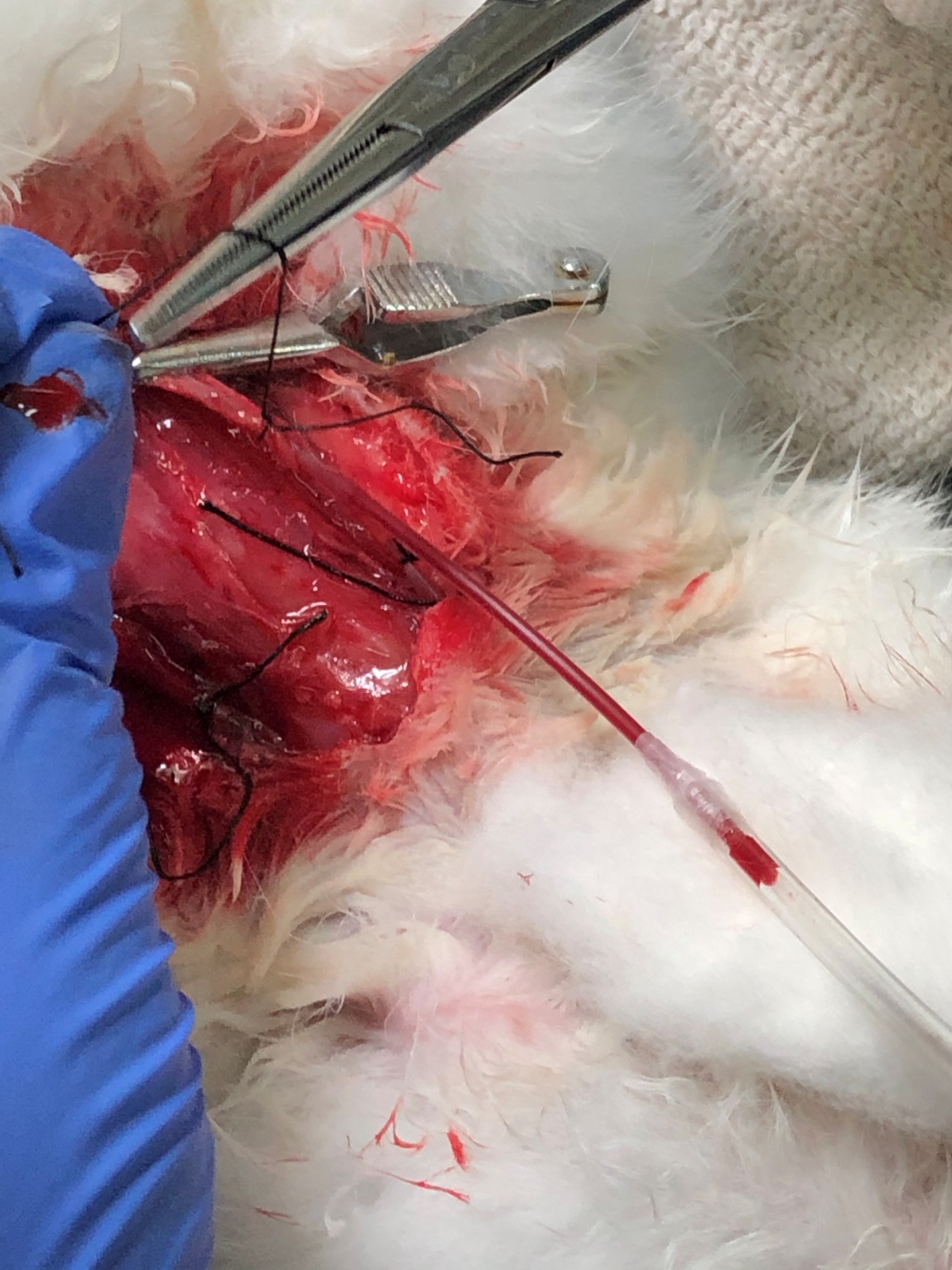

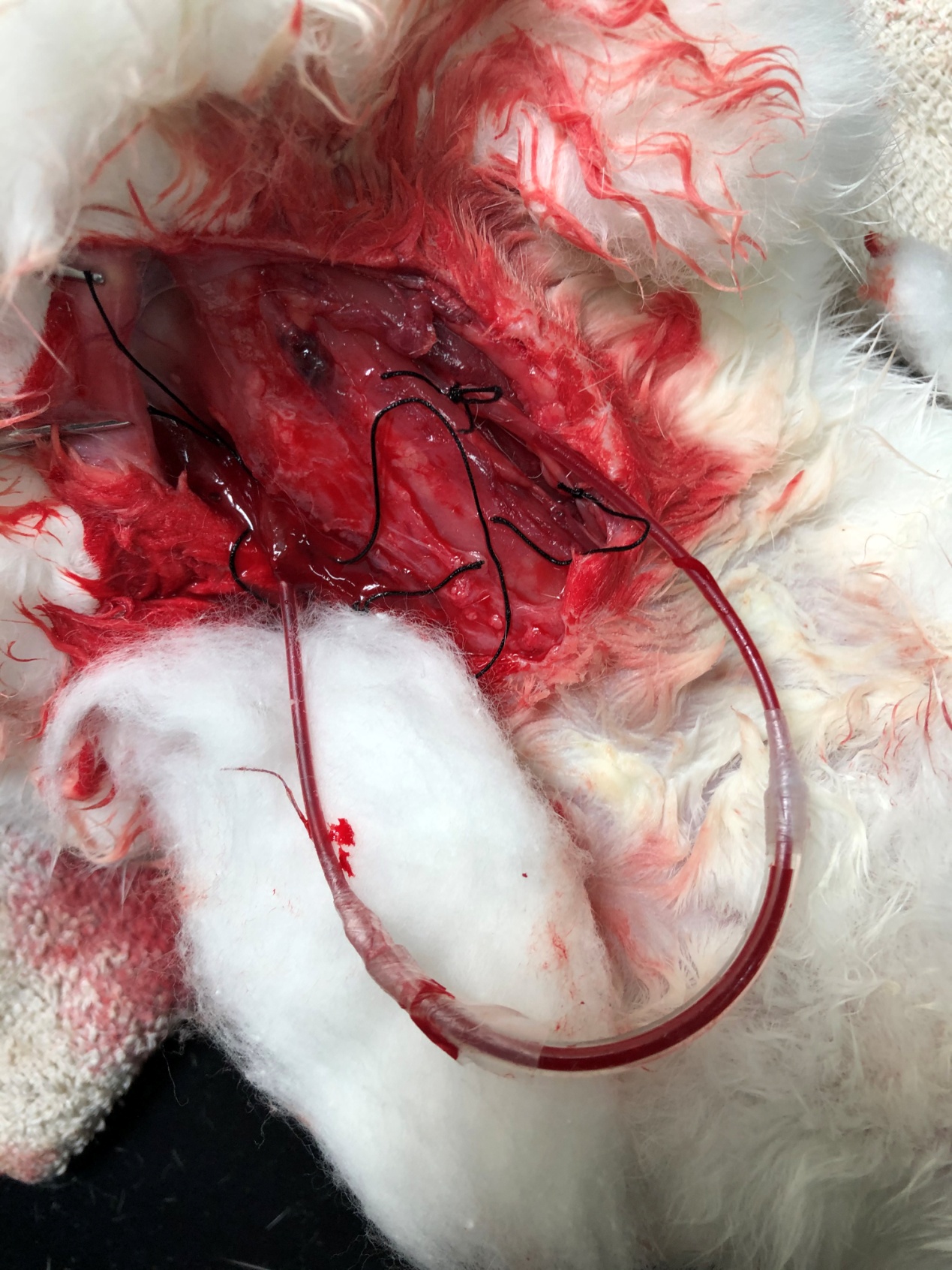

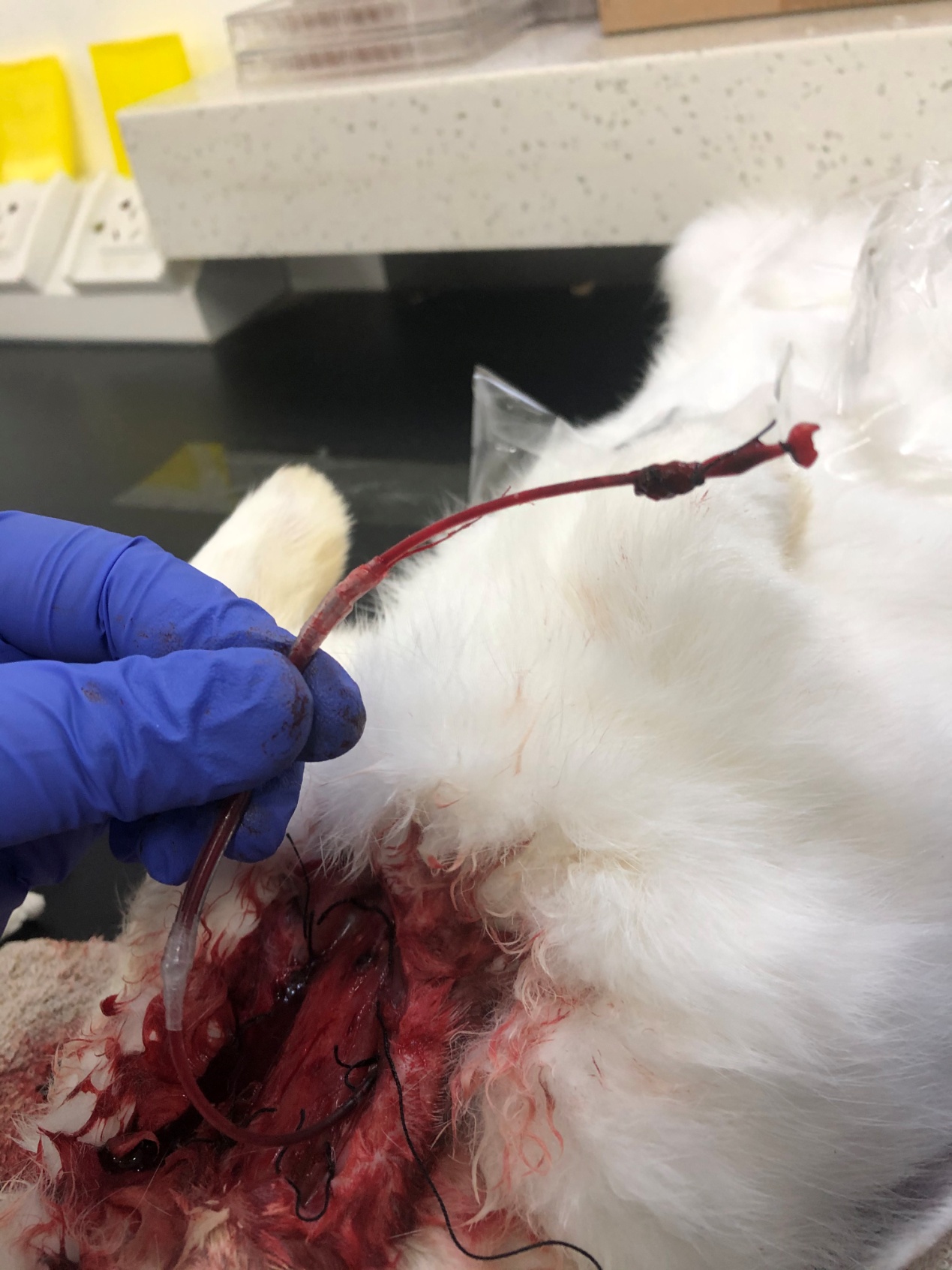

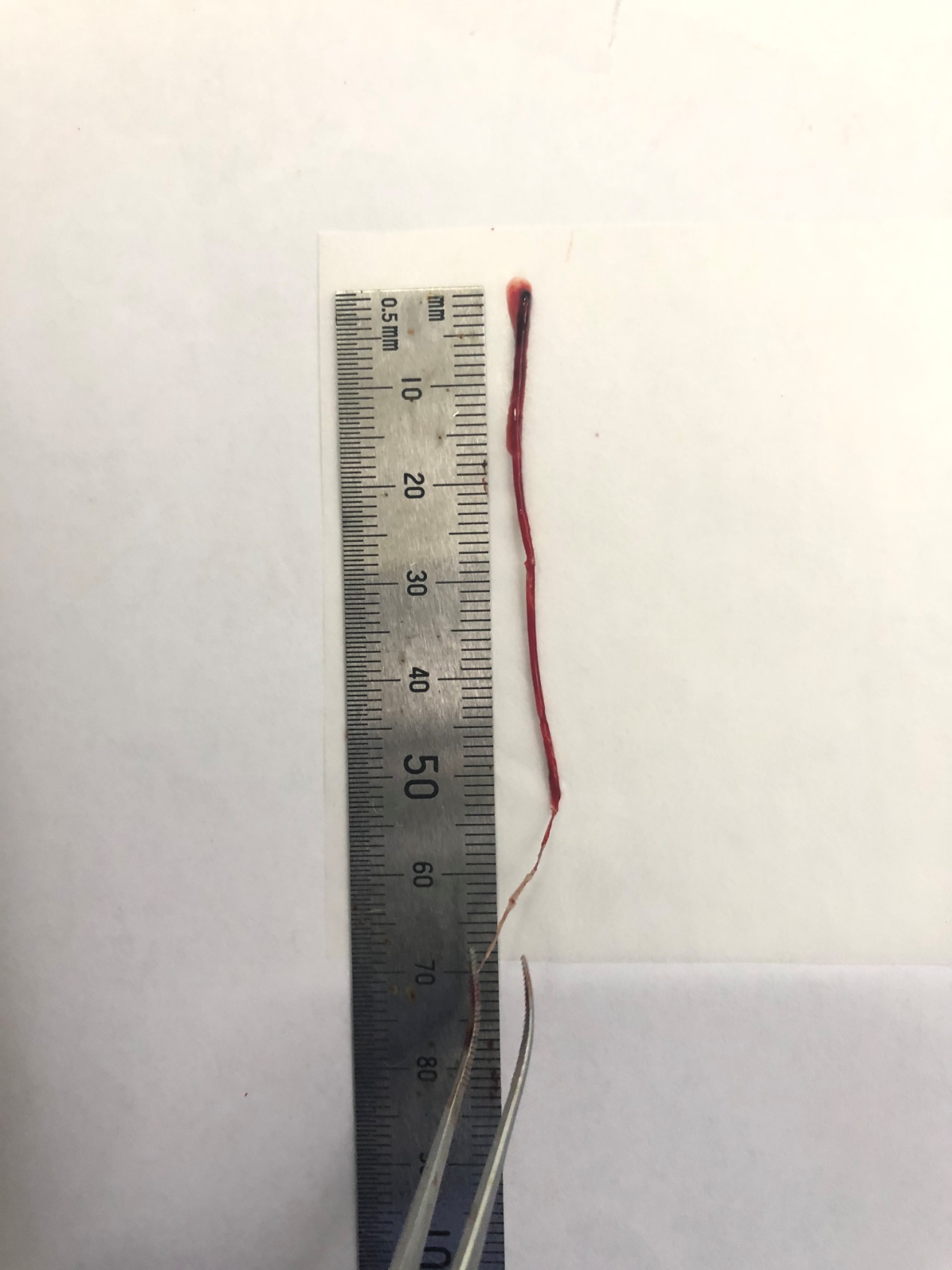


Figure 1：Animal modeling and sample collection.


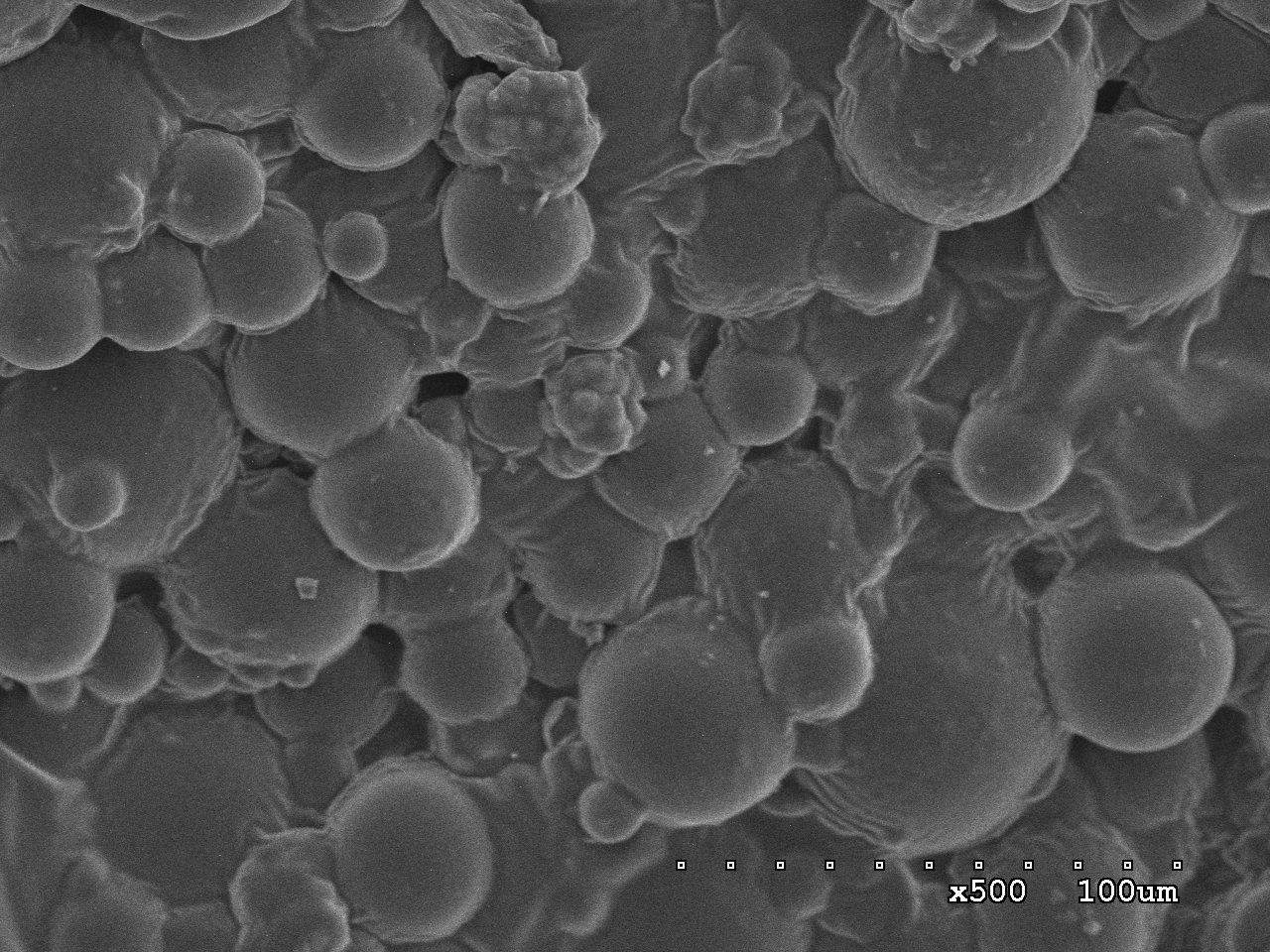


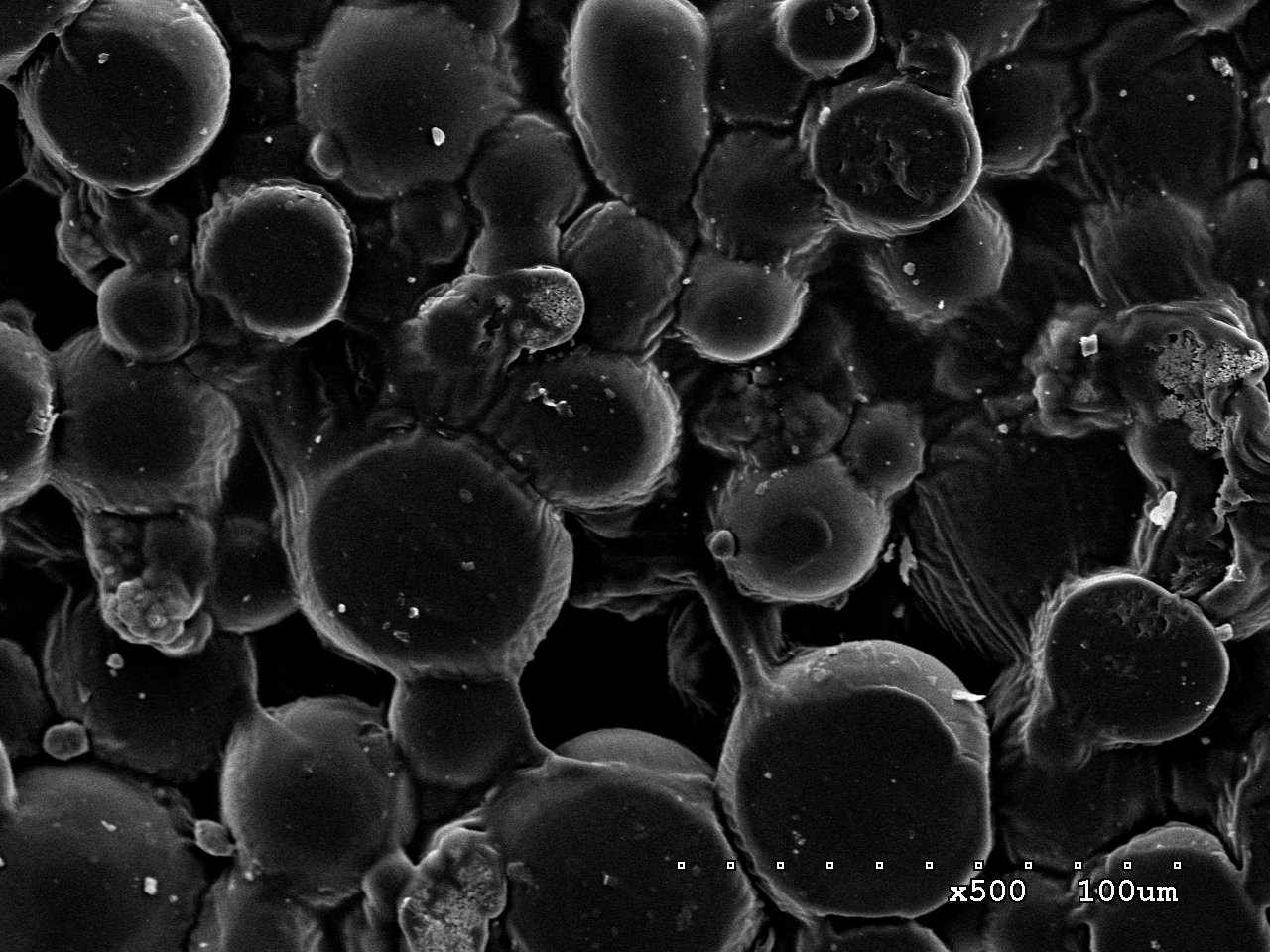


Figure 2:Characterization of PMMA and ES-PMMA by electron microscopy.

Table 1: Raw data of the weight of thrombus formed in different groups.

| \| group \| preoperative（g） \| postoperative（g） \| thrombus weight（g） \| bone cement length(cm) \| thrombus weight/bone cement length(g/cm) \| standard deviation \| \| \| --- \| --- \| --- \| --- \| --- \| --- \| --- \| --- \| \| Con1 \| 0.0113 \| 0.0563 \| 0.0450 \| 5 \| 0.009 \|  \|  \| \| Con2 \| 0.0302 \| 0.0704 \| 0.0402 \| 6 \| 0.0067 \|  \|  \| \| Con3 \| 0.0245 \| 0.0718 \| 0.0473 \| 7 \| 0.006757143 \|  \|  \| \| Con4 \| 0.0198 \| 0.0517 \| 0.0319 \| 5.5 \| 0.0058 \|  \|  \| \| Con5 \| 0.0186 \| 0.0602 \| 0.0416 \| 5.5 \| 0.007563636 \|  \|  \| \| Con6 \| 0.0227 \| 0.0712 \| 0.0485 \| 6 \| 0.008083333 \|  \|  \| \| Con7 \| 0.0298 \| 0.0735 \| 0.0437 \| 6.5 \| 0.006723077 \|  \|  \| \| Con8 \| 0.0216 \| 0.0688 \| 0.0472 \| 6 \| 0.007866667 \|  \|  \| \| Con9 \| 0.0202 \| 0.0603 \| 0.0401 \| 5.5 \| 0.007290909 \|  \|  \| \| Con10 \| 0.0216 \| 0.0737 \| 0.0521 \| 7 \| 0.007442857 \|  \|  \| \|  \|  \| average \|  \|  \| 0.007322762 \| 0.000891133 \|  \| \| M1 \| 0.0217 \| 0.0510 \| 0.0293 \| 7 \| 0.004185714 \|  \|  \| \| M2 \| 0.0122 \| 0.0530 \| 0.0408 \| 6.5 \| 0.006276923 \|  \|  \| \| M3 \| 0.0157 \| 0.0491 \| 0.0334 \| 5.5 \| 0.006072727 \|  \|  \| \| M4 \| 0.0136 \| 0.0417 \| 0.0281 \| 5 \| 0.00562 \|  \|  \| \| M5 \| 0.0203 \| 0.0518 \| 0.0315 \| 5.5 \| 0.005727273 \|  \|  \| \| M6 \| 0.0194 \| 0.0529 \| 0.0335 \| 6 \| 0.005583333 \|  \|  \| \| M7 \| 0.0214 \| 0.0621 \| 0.0407 \| 7 \| 0.005814286 \|  \|  \| \| M8 \| 0.0273 \| 0.0632 \| 0.0359 \| 7 \| 0.005128571 \|  \|  \| \| M9 \| 0.0192 \| 0.0588 \| 0.0396 \| 6 \| 0.0066 \|  \|  \| \| M10 \| 0.0313 \| 0.0556 \| 0.0243 \| 5.5 \| 0.004418182 \|  \|  \| \|  \|  \| average \|  \|  \| 0.005542701 \| 0.000769618 \|  \| |  |  |  |  |  |  |  |
| --- | --- | --- | --- | --- | --- | --- | --- | --- | --- | --- | --- | --- | --- | --- | --- | --- | --- | --- | --- | --- | --- | --- | --- | --- | --- | --- | --- | --- | --- | --- | --- | --- | --- | --- | --- | --- | --- | --- | --- | --- | --- | --- | --- | --- | --- | --- | --- | --- | --- | --- | --- | --- | --- | --- | --- | --- | --- | --- | --- | --- | --- | --- | --- | --- | --- | --- | --- | --- | --- | --- | --- | --- | --- | --- | --- | --- | --- | --- | --- | --- | --- | --- | --- | --- | --- | --- | --- | --- | --- | --- | --- | --- | --- | --- | --- | --- | --- | --- | --- | --- | --- | --- | --- | --- | --- | --- | --- | --- | --- | --- | --- | --- | --- | --- | --- | --- | --- | --- | --- | --- | --- | --- | --- | --- | --- | --- | --- | --- | --- | --- | --- | --- | --- | --- | --- | --- | --- | --- | --- | --- | --- | --- | --- | --- | --- | --- | --- | --- | --- | --- | --- | --- | --- | --- | --- | --- | --- | --- | --- | --- | --- | --- | --- | --- | --- | --- | --- | --- | --- | --- | --- | --- | --- | --- | --- | --- | --- | --- | --- | --- | --- | --- | --- | --- | --- | --- | --- | --- | --- | --- | --- |
|  |  |  |  |  |  |  |  |
|  |  |  |  |  |  |  |  |
|  |  |  |  |  |  |  |  |
|  |  |  |  |  |  |  |  |
| 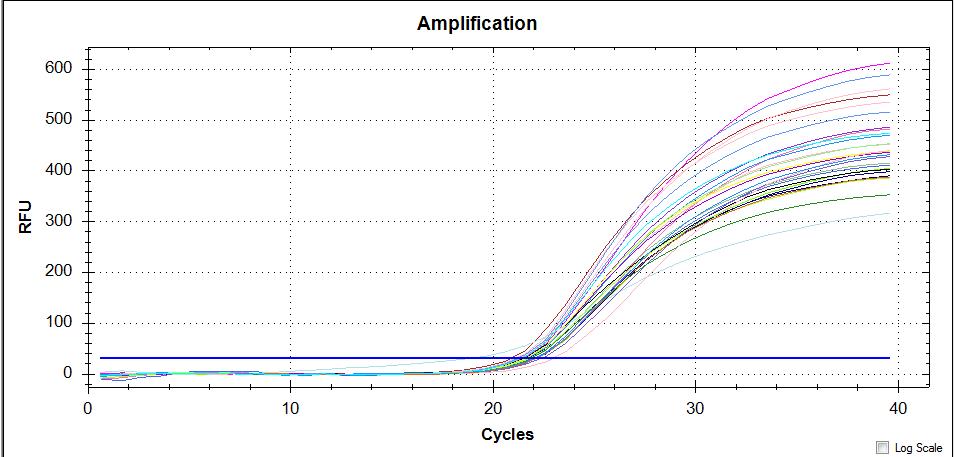  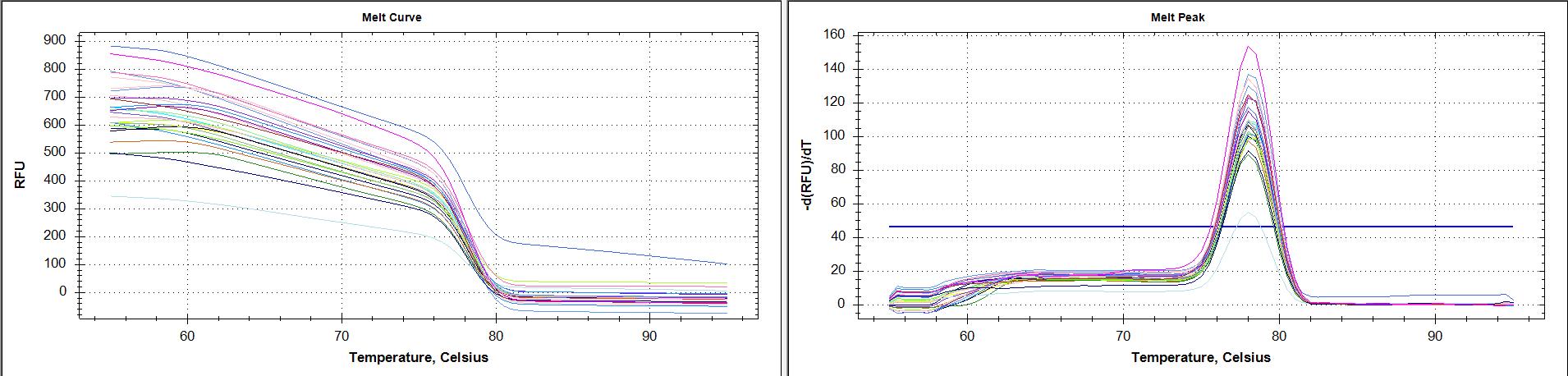 |  |  |  |  |  |  |  |
|  |  |  |  |  |  |  |  |
|  |  |  |  |  |  |  |  |
| Figure 3a: Amplification curve and dissolve curve of CD31. |  |  |  |  |  |  |  |
|  |  |  |  |  |  |  |  |
|  |  |  |  |  |  |  |  |
|  |  |  |  |  |  |  |  |
|  |  |  |  |  |  |  |  |
|  |  |  |  |  |  |  |  |
|  |  |  |  |  |  |  |  |
|  |  |  |  |  |  |  |  |
|  |  |  |  |  |  |  |  |
| 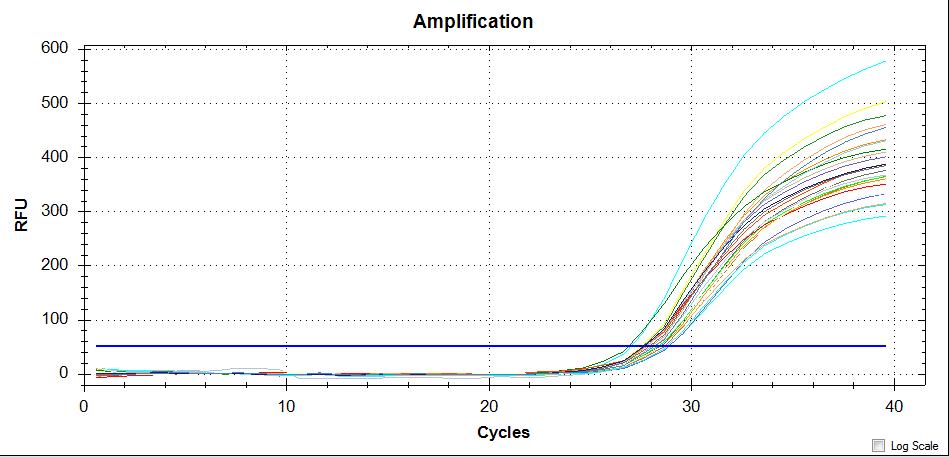 |  |  |  |  |  |  |  |
|  |  |  |  |  |  |  |  |
|  |  |  |  |  |  |  |  |
|  |  |  |  |  |  |  |  |
|  |  |  |  |  |  |  |  |
|  |  |  |  |  |  |  |  |


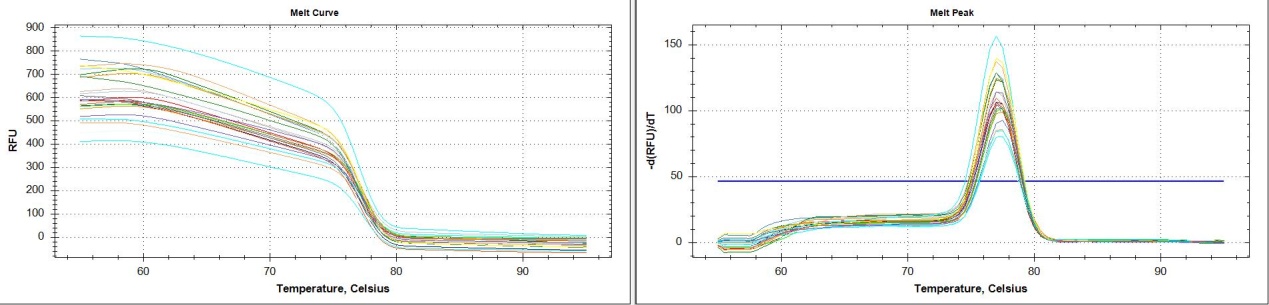


Figure 3b: Amplification curve and dissolve curve of CD40.

|  |  |  |  |  |
| --- | --- | --- | --- | --- |
|  |  |  |  |  |
|  |  |  |  |  |
|  |  |  |  |  |
|  |  |  |  |  |
|  |  |  |  |  |
|  |  |  |  |  |
|  |  |  |  |  |
|  |  |  |  |  |


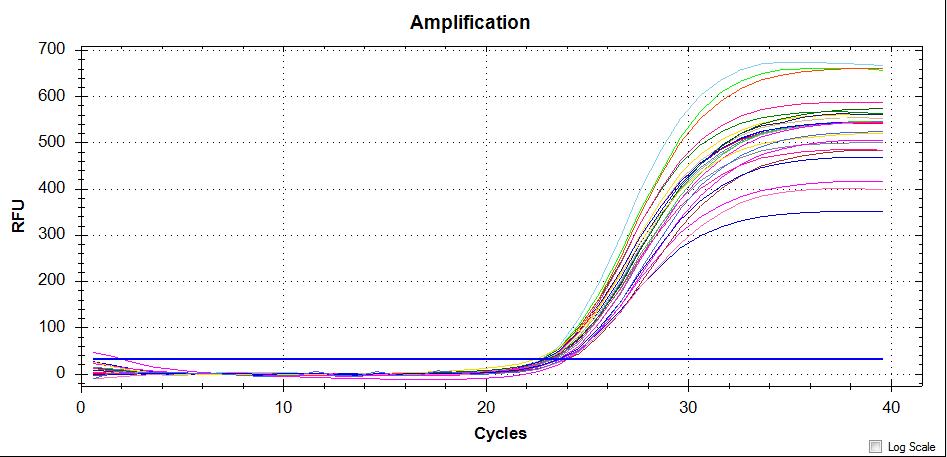

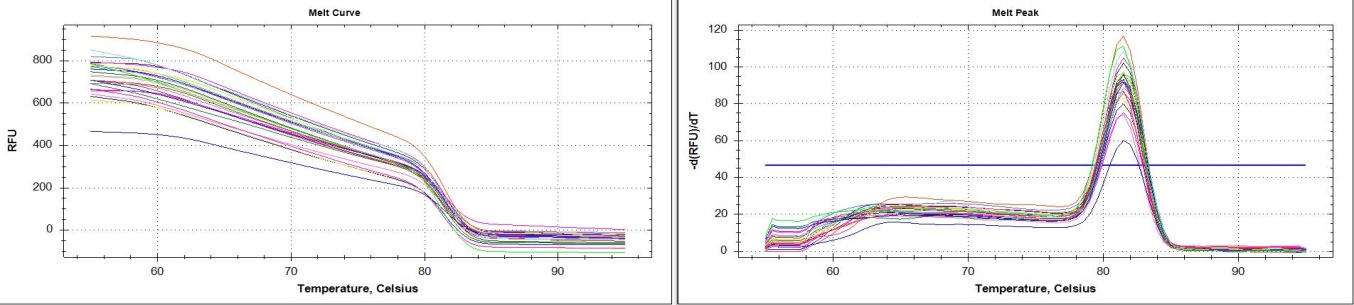


Figure 3c: Amplification curve and dissolve curve of CD62p.


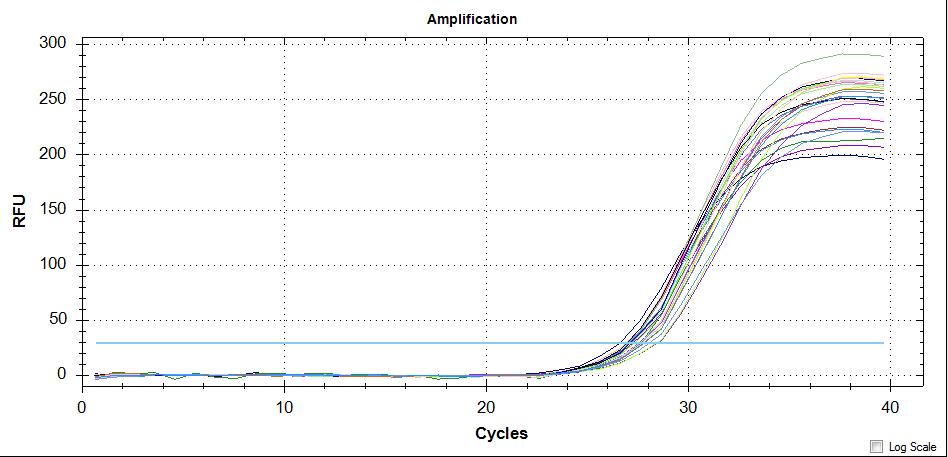

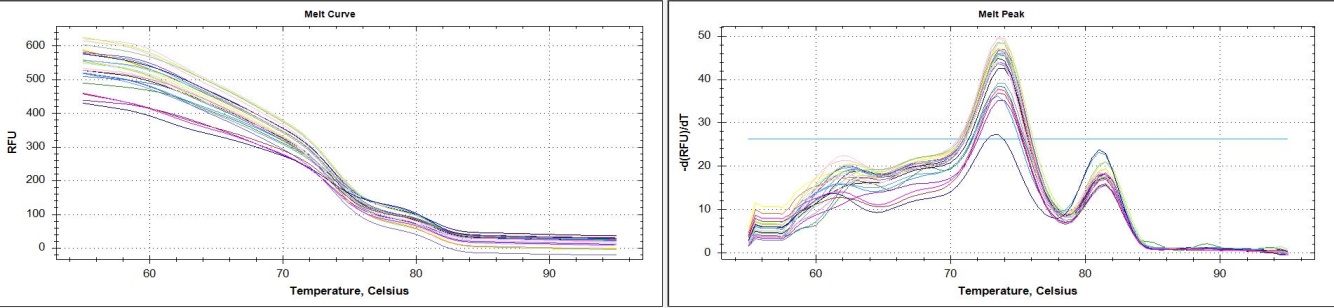


Figure 3d: Amplification curve and dissolve curve of CD106.


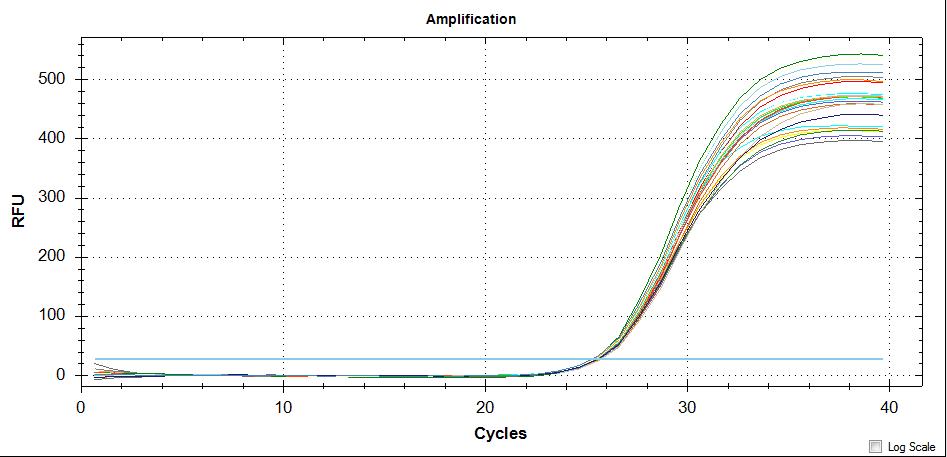


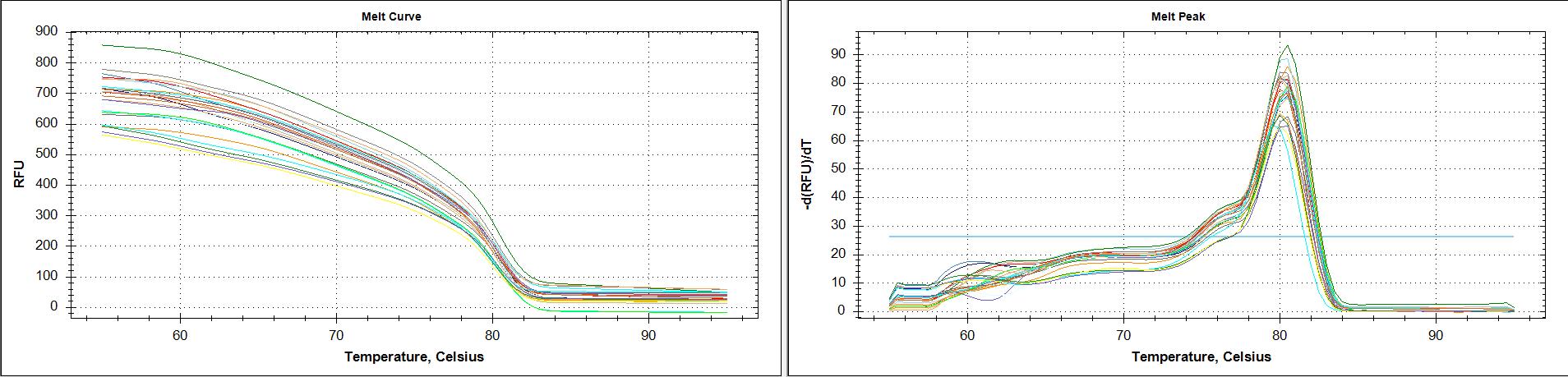


Figure 3e: Amplification curve and dissolve curve of endothelin.


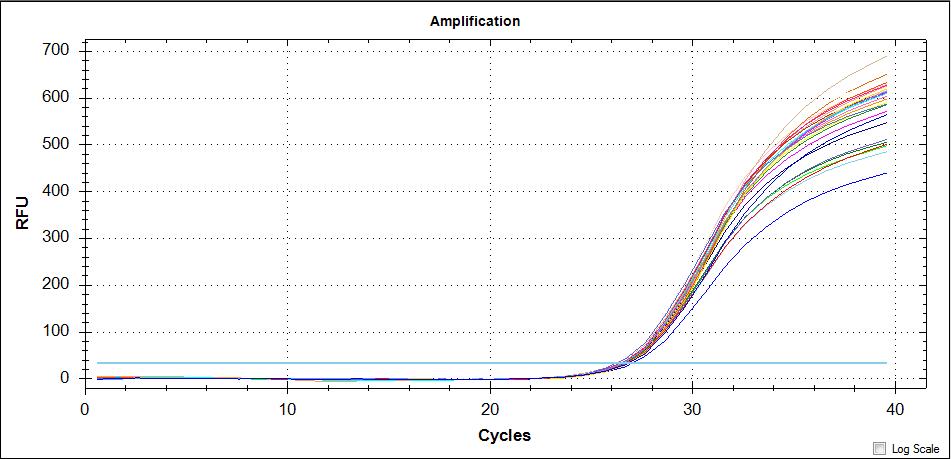

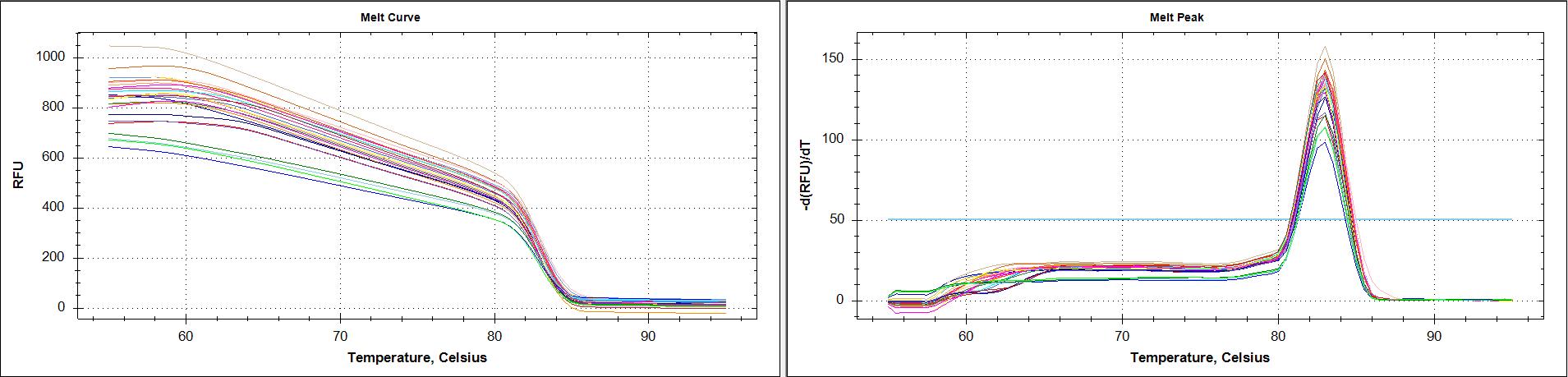


Figure 3f: Amplification curve and dissolve curve of thrombomodulin.


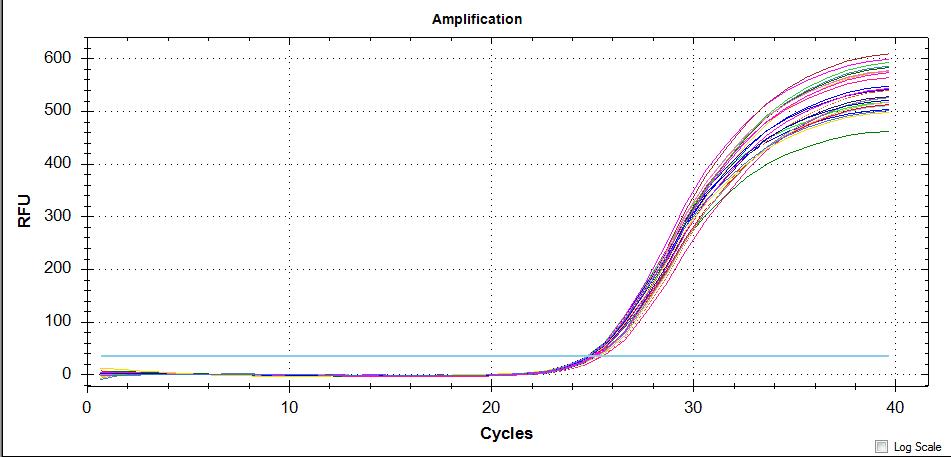

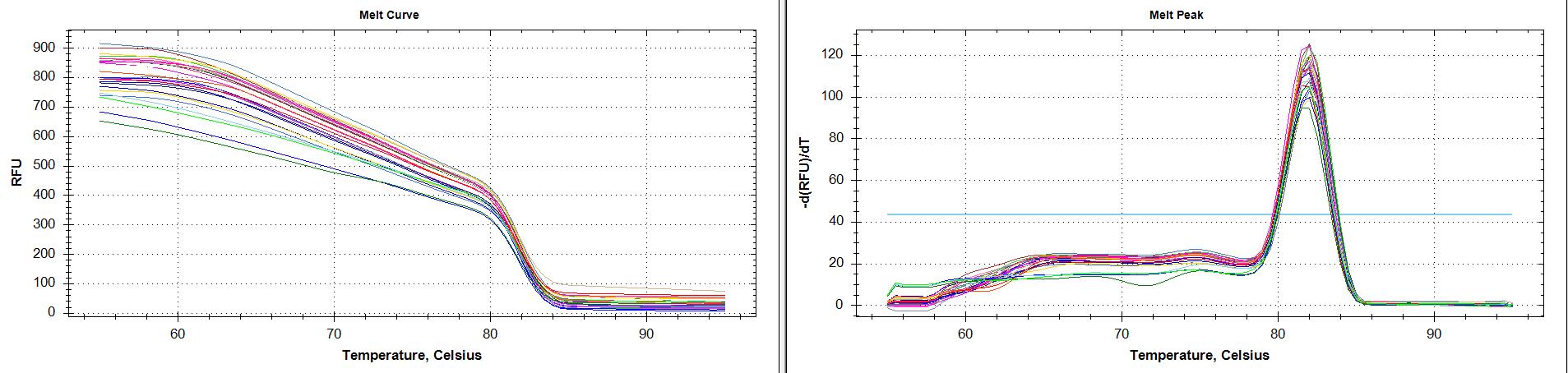


Figure 3g: Amplification curve and dissolve curve of GAPDH.


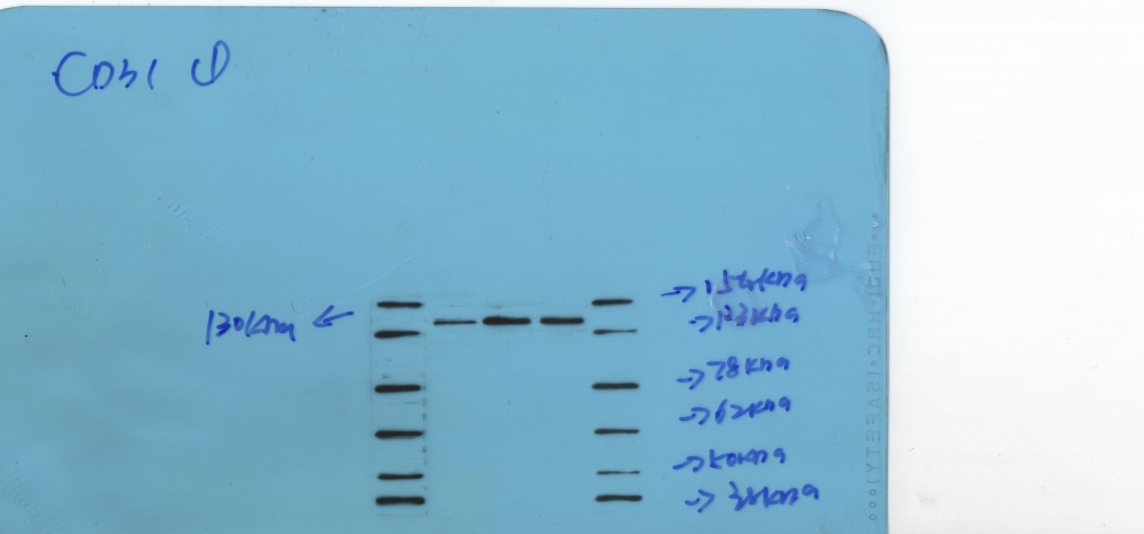

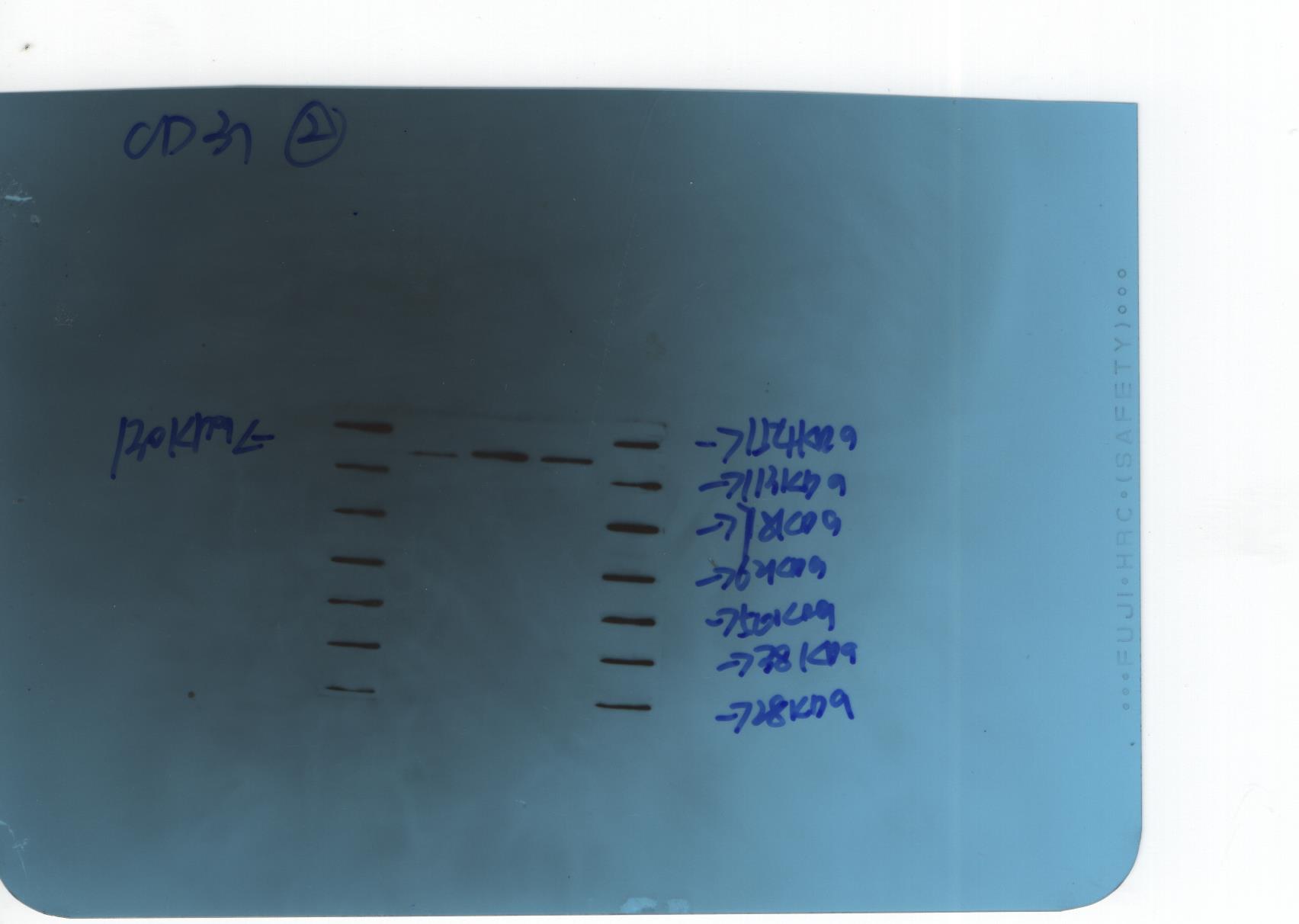


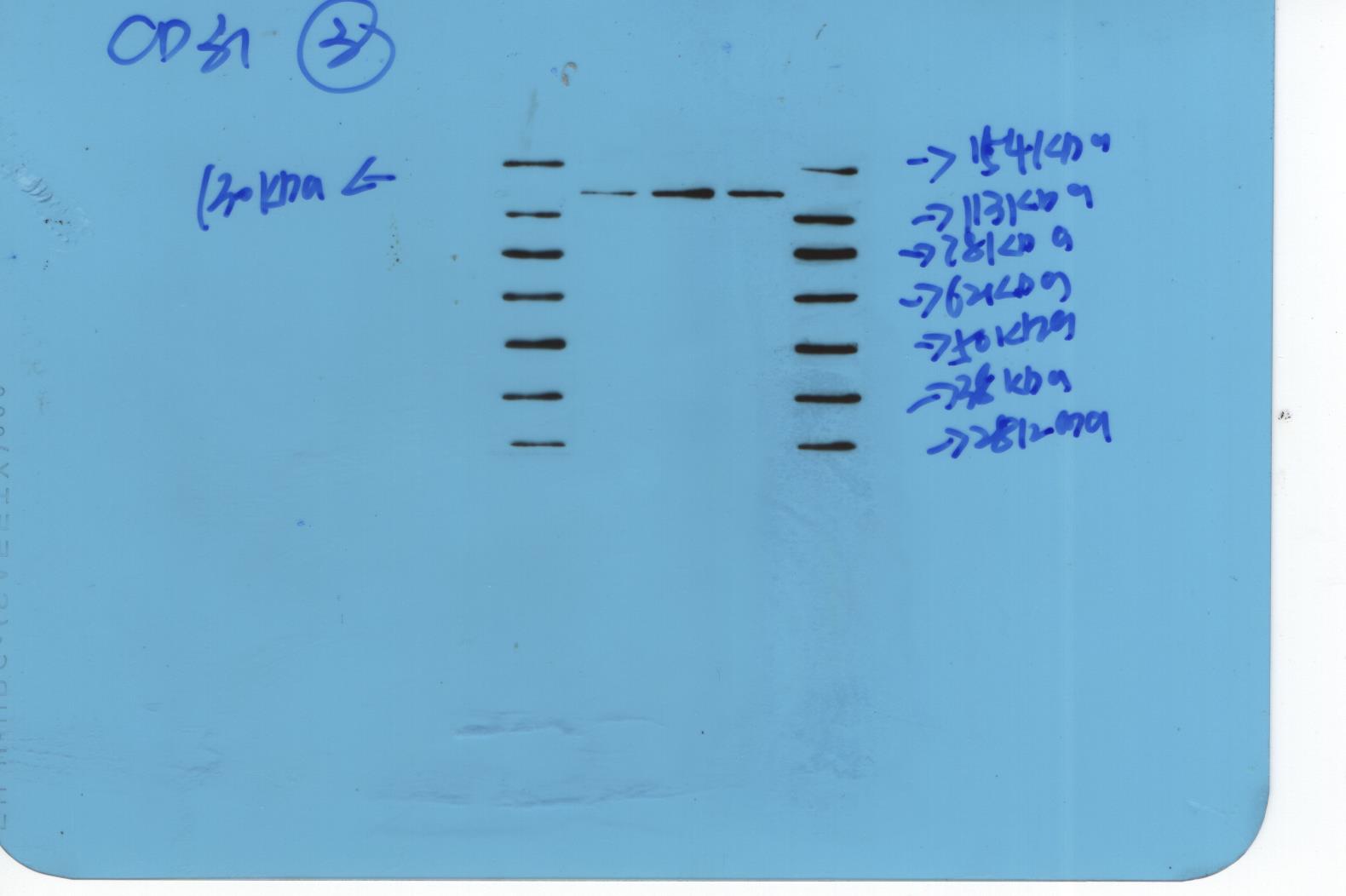


Figure 4a: The original blots/gels of CD31, The figure in the munuscript was cropped from the third one.


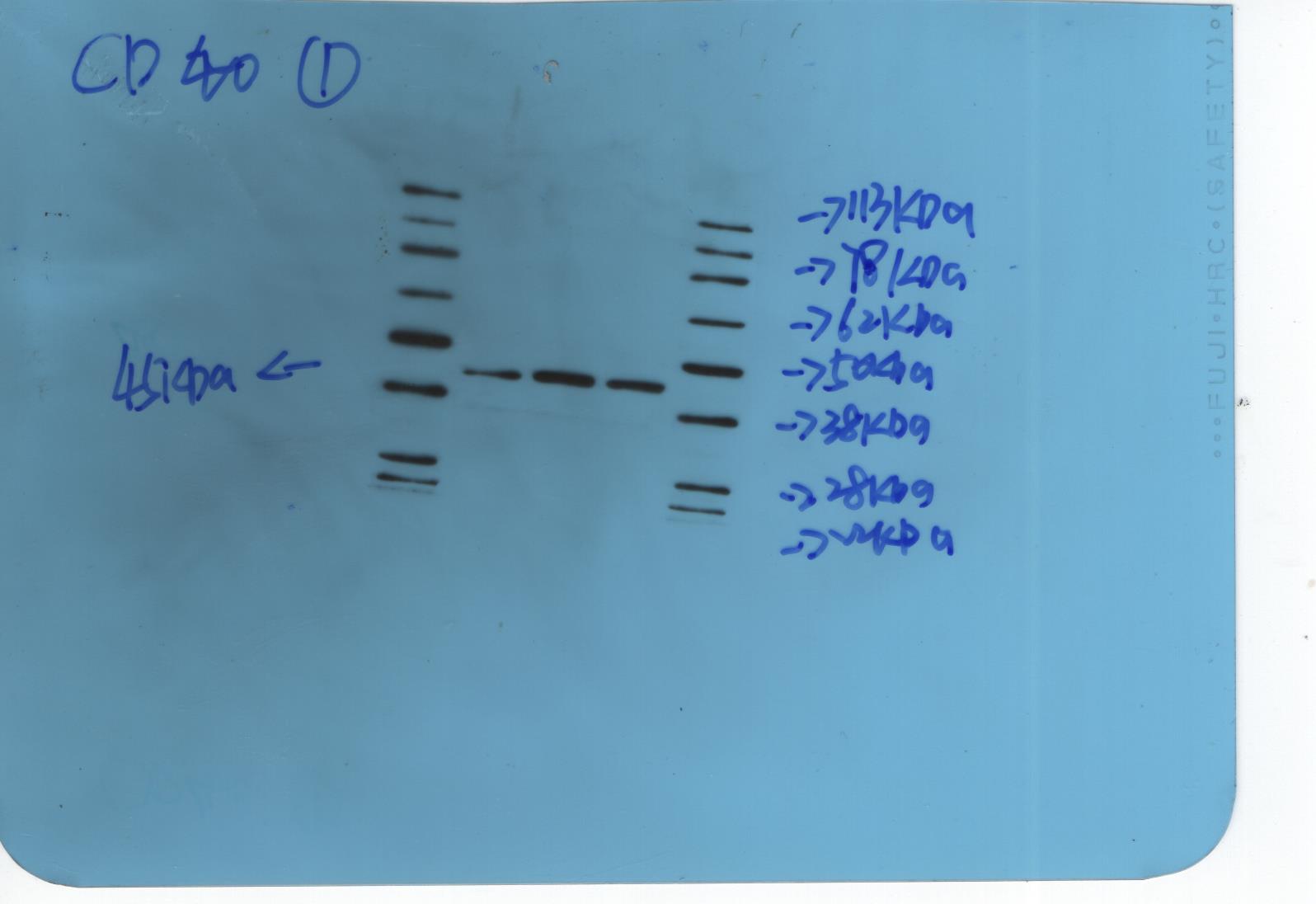

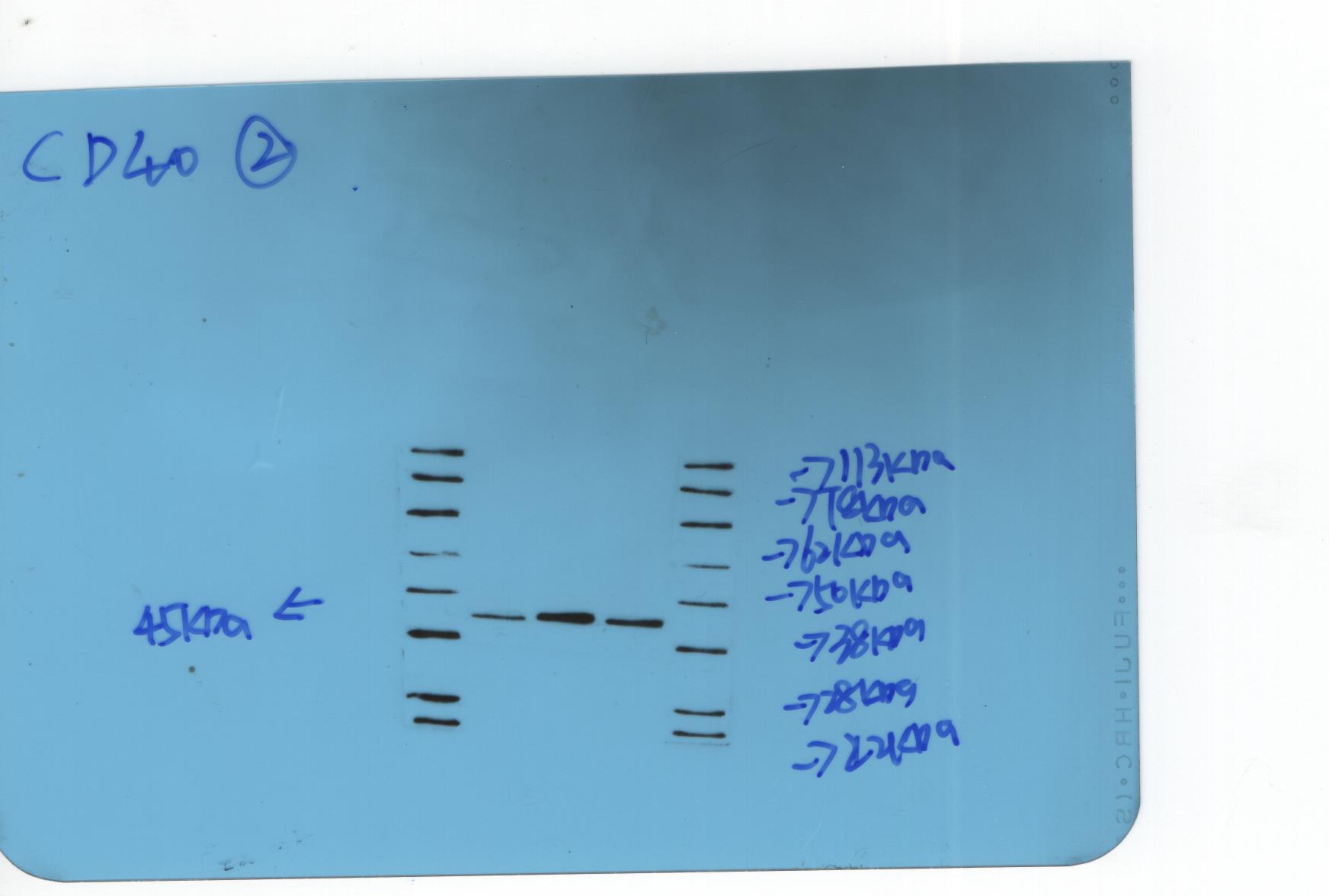

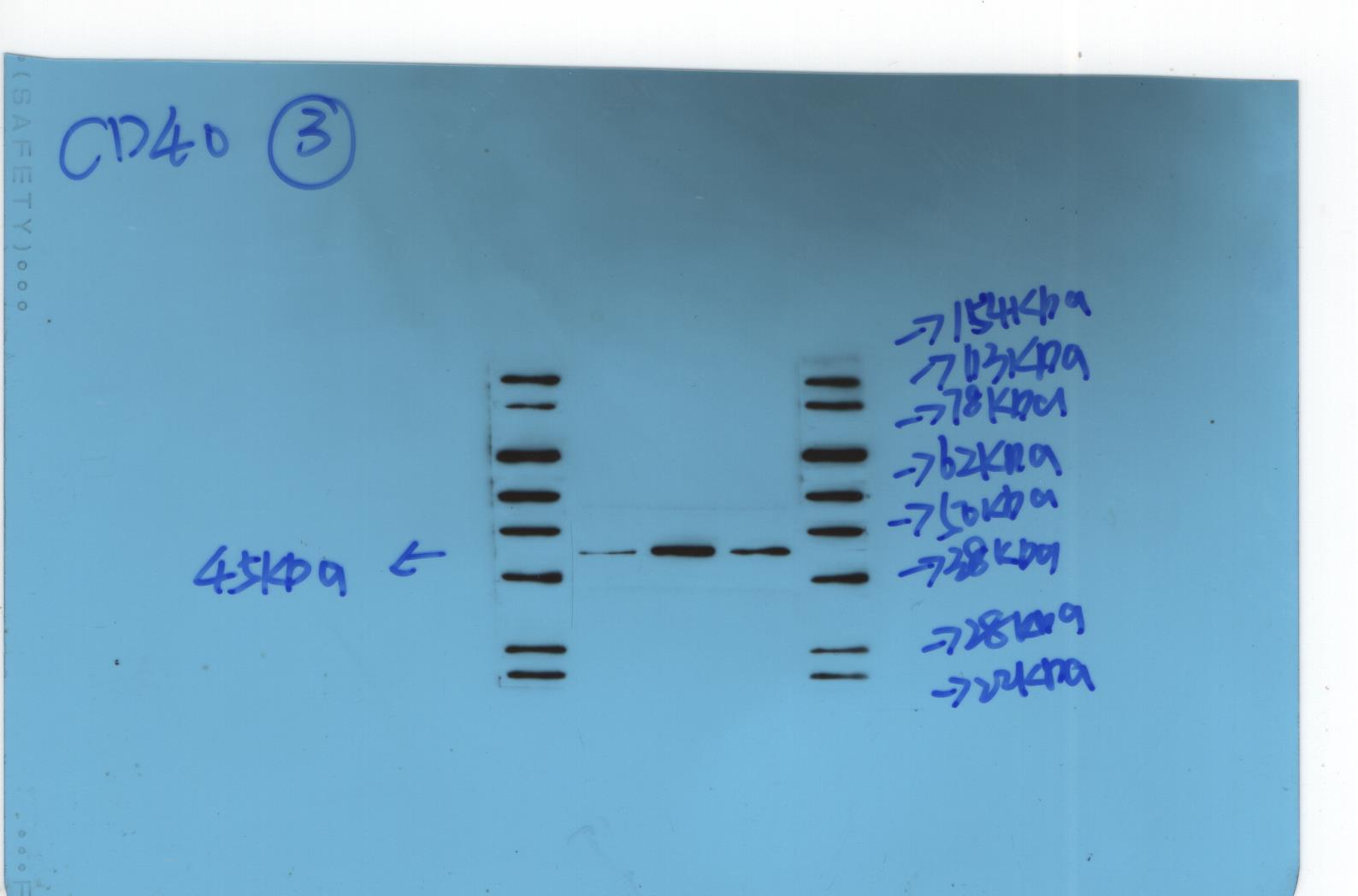


Figure 4b: The original blots/gels of CD40, The figure in the munuscript was cropped from the third one.


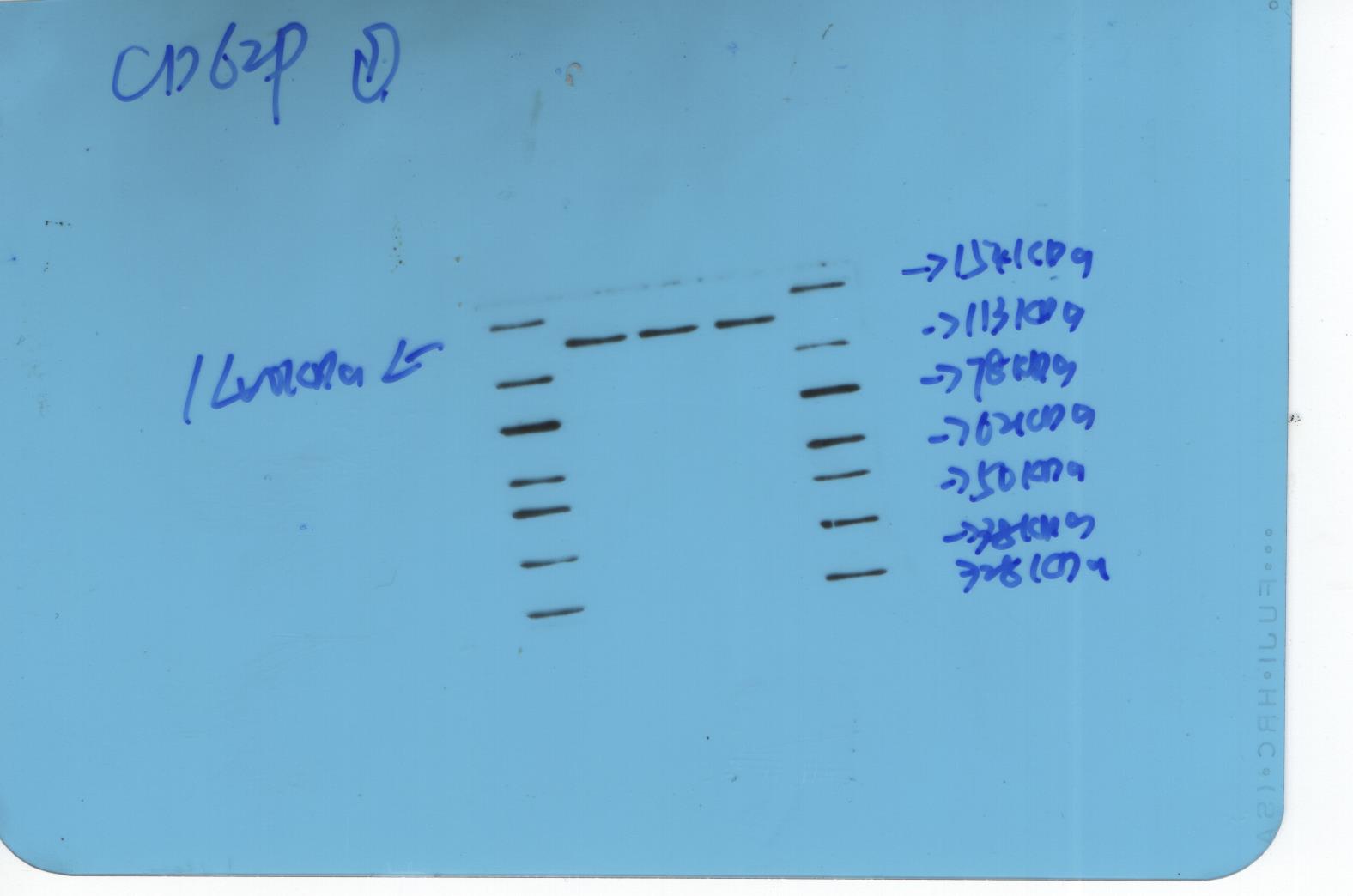

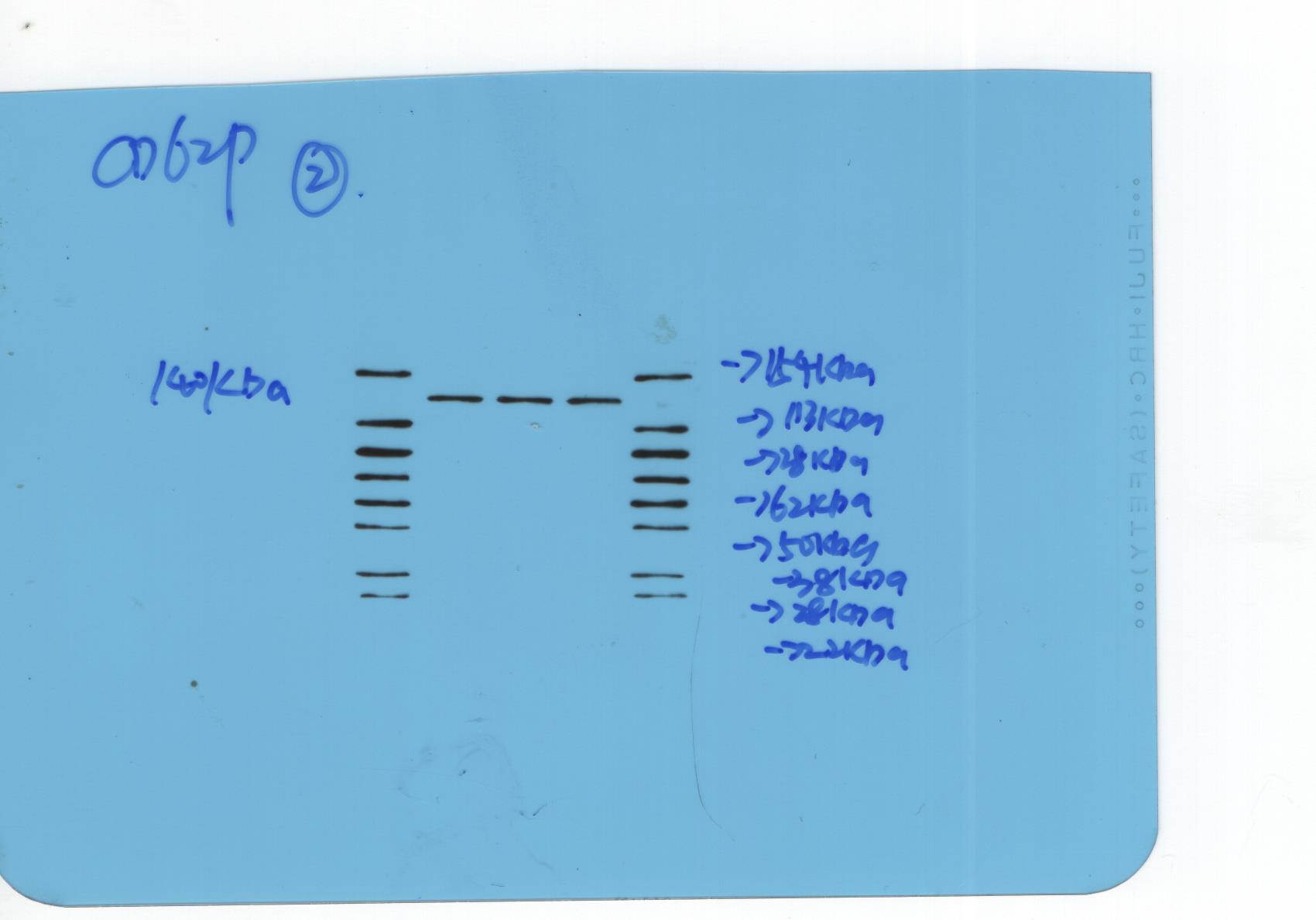

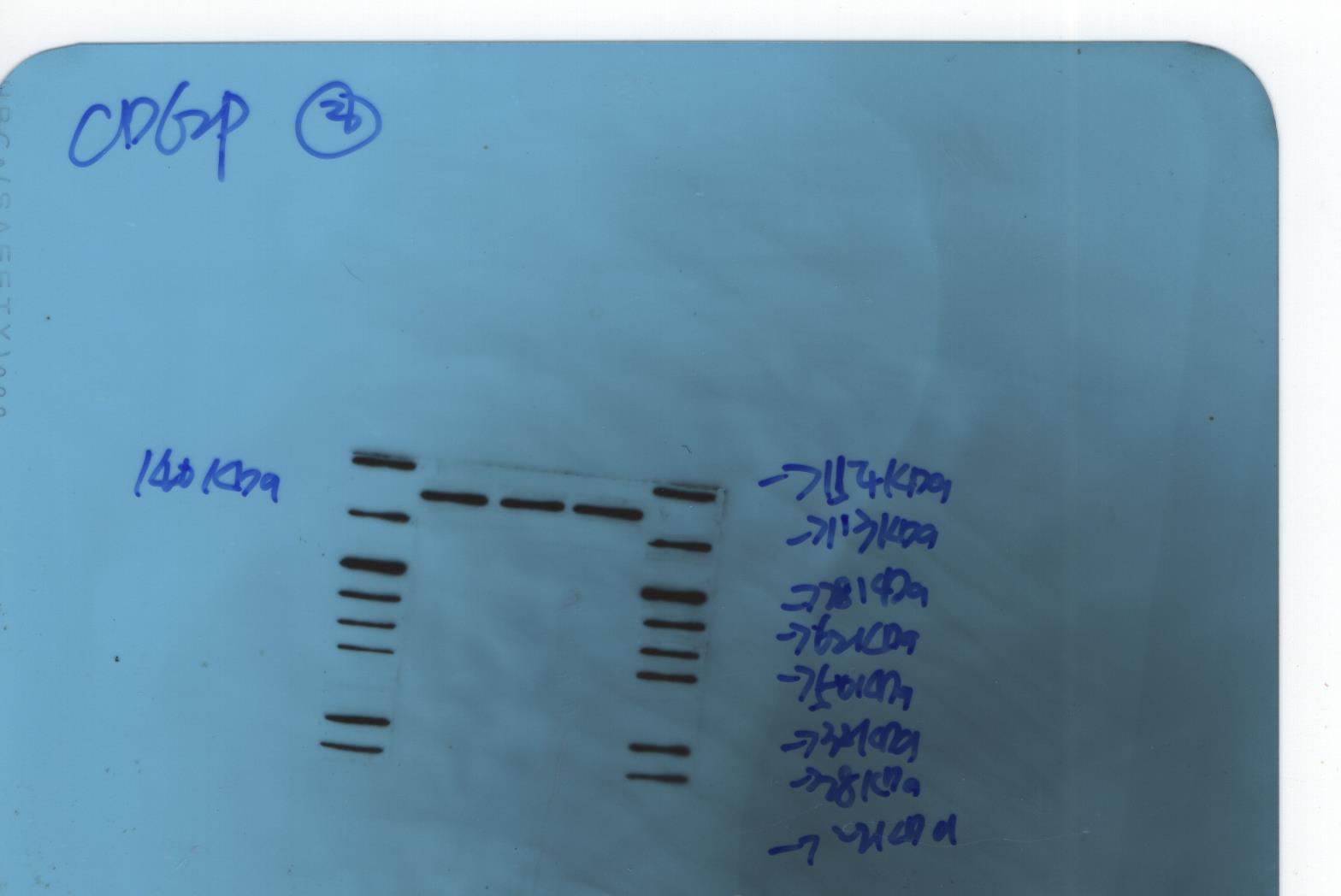


Figure 4c: The original blots/gels of CD62p, The figure in the munuscript was cropped from the second one.


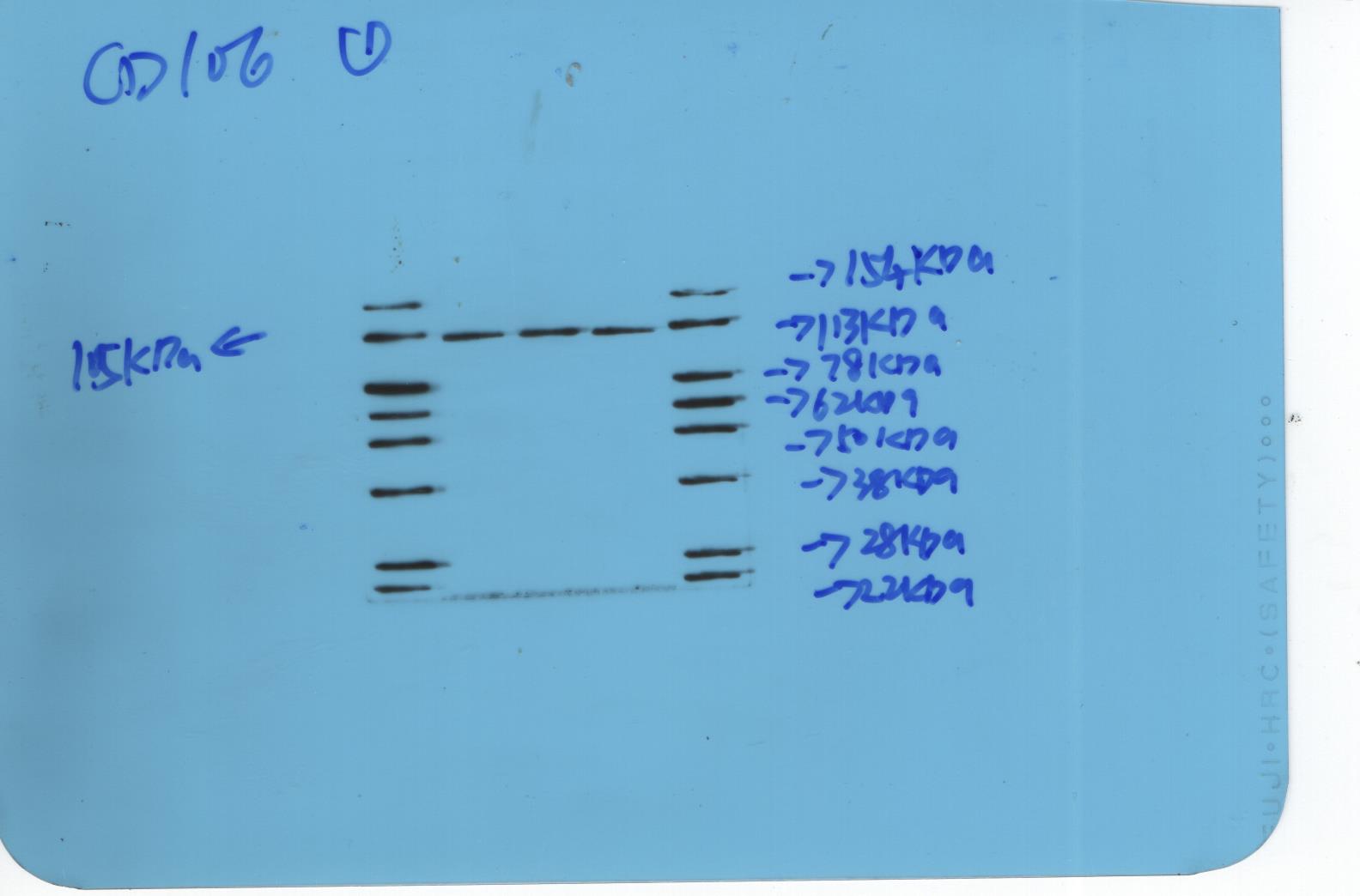

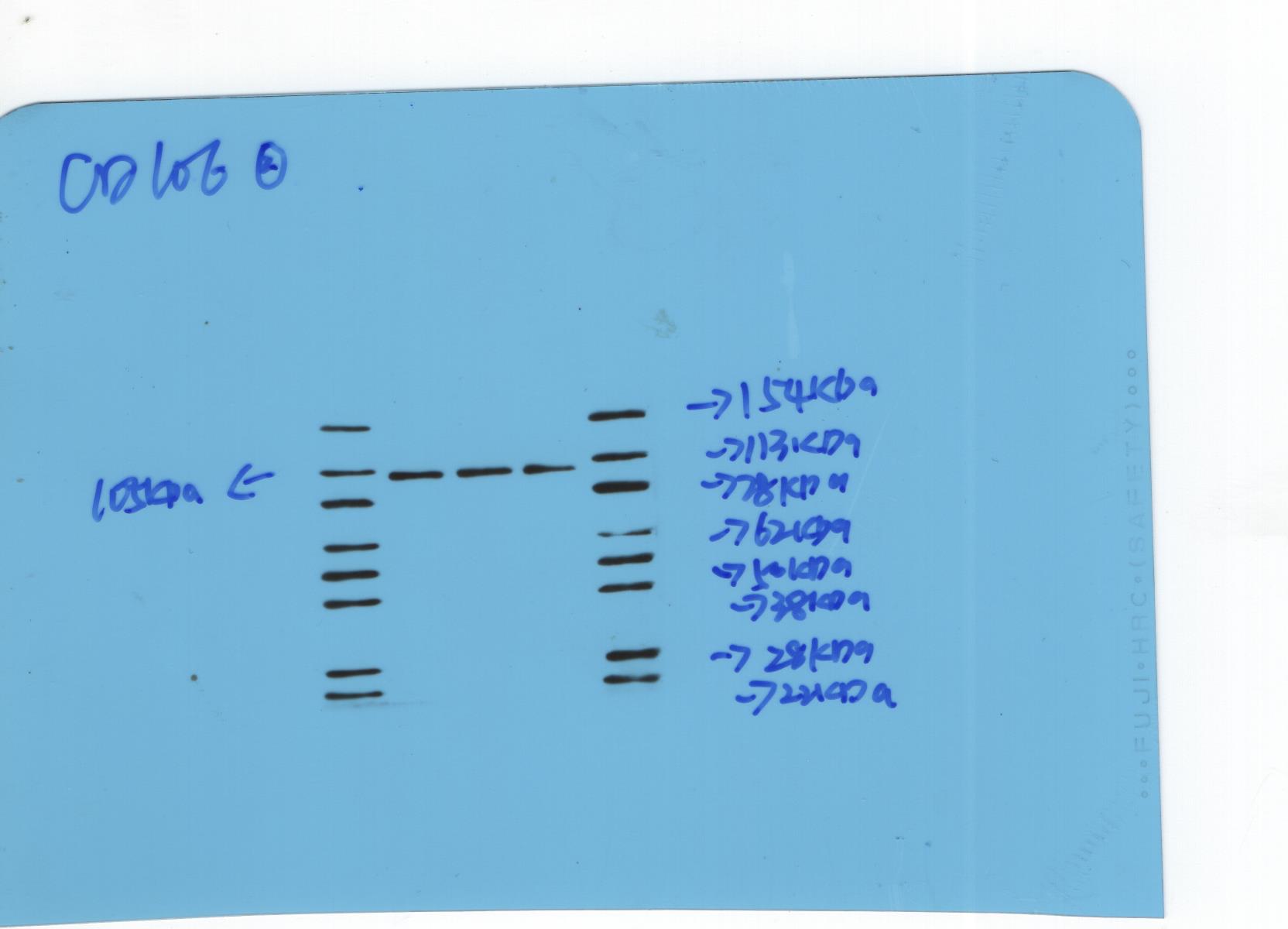

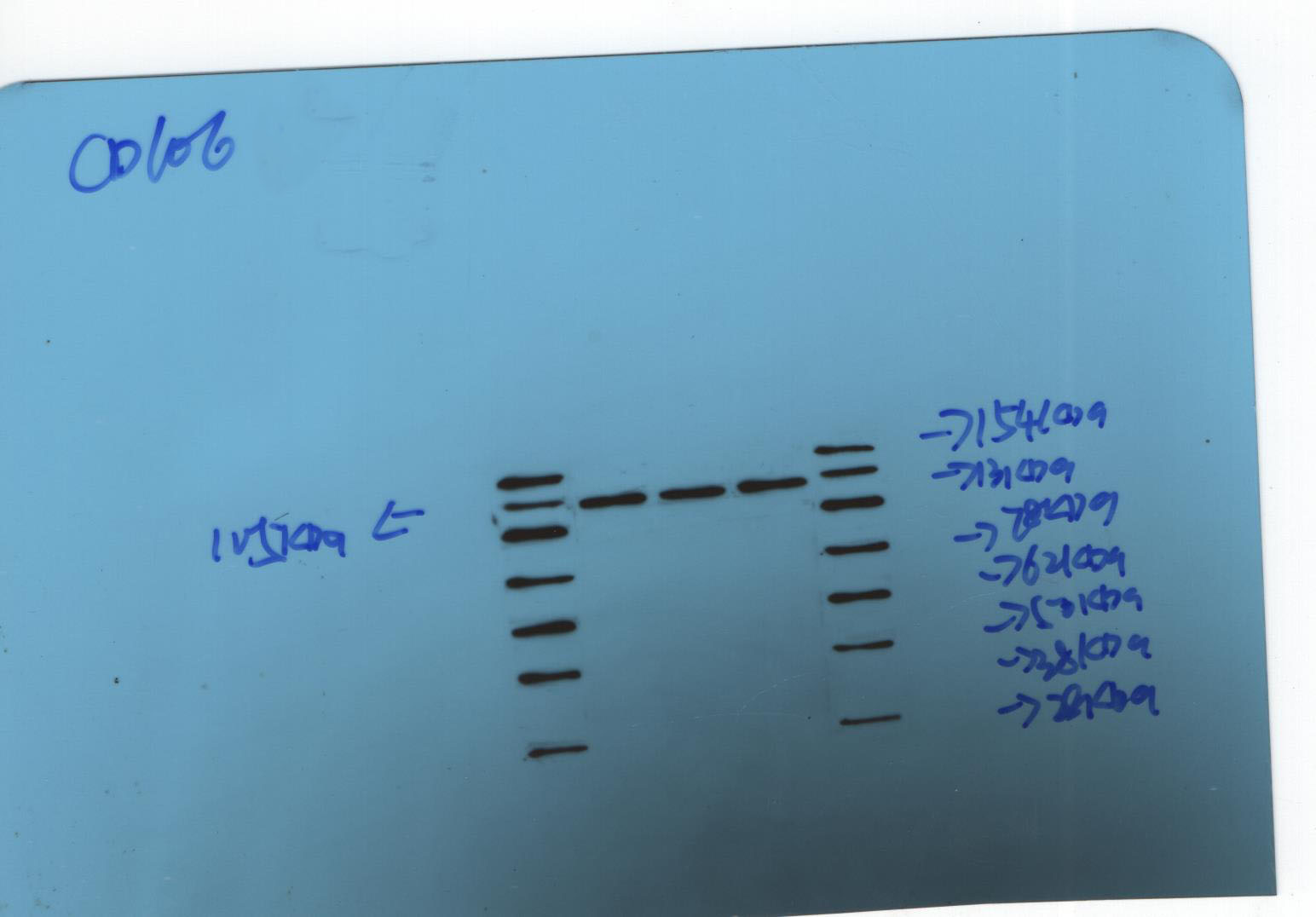


Figure 4d: The original blots/gels of CD106, The figure in the munuscript was cropped from the first one.


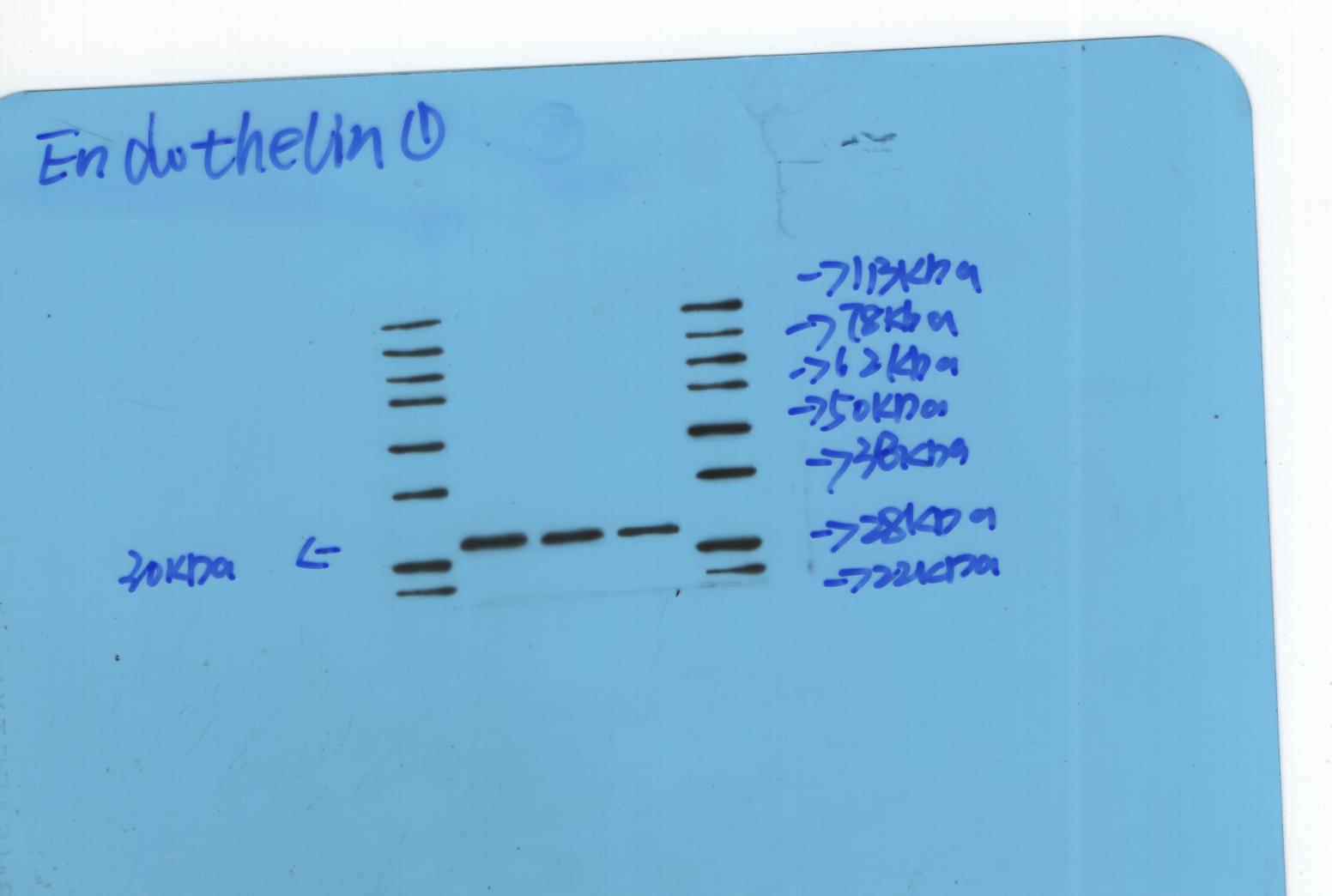

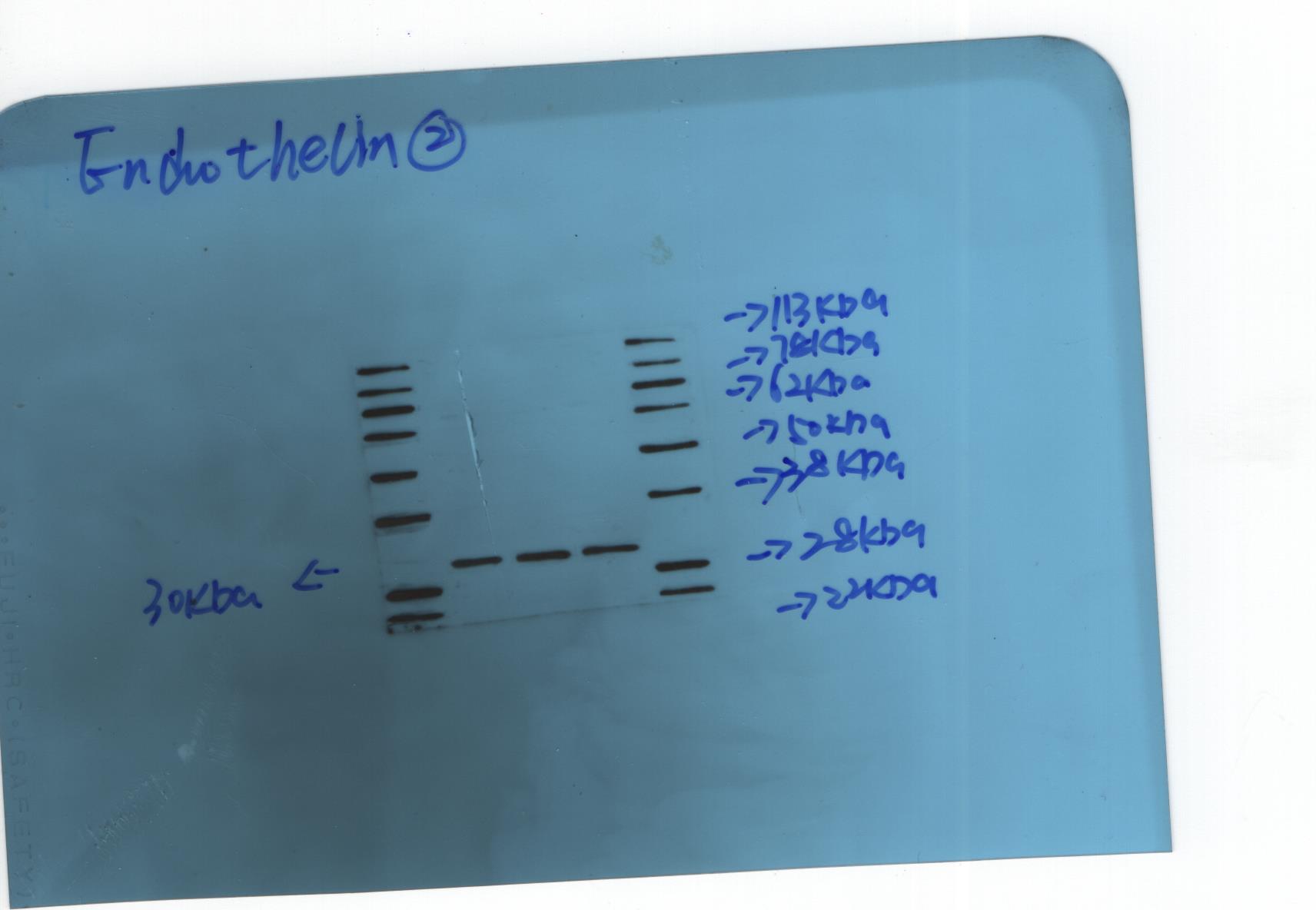

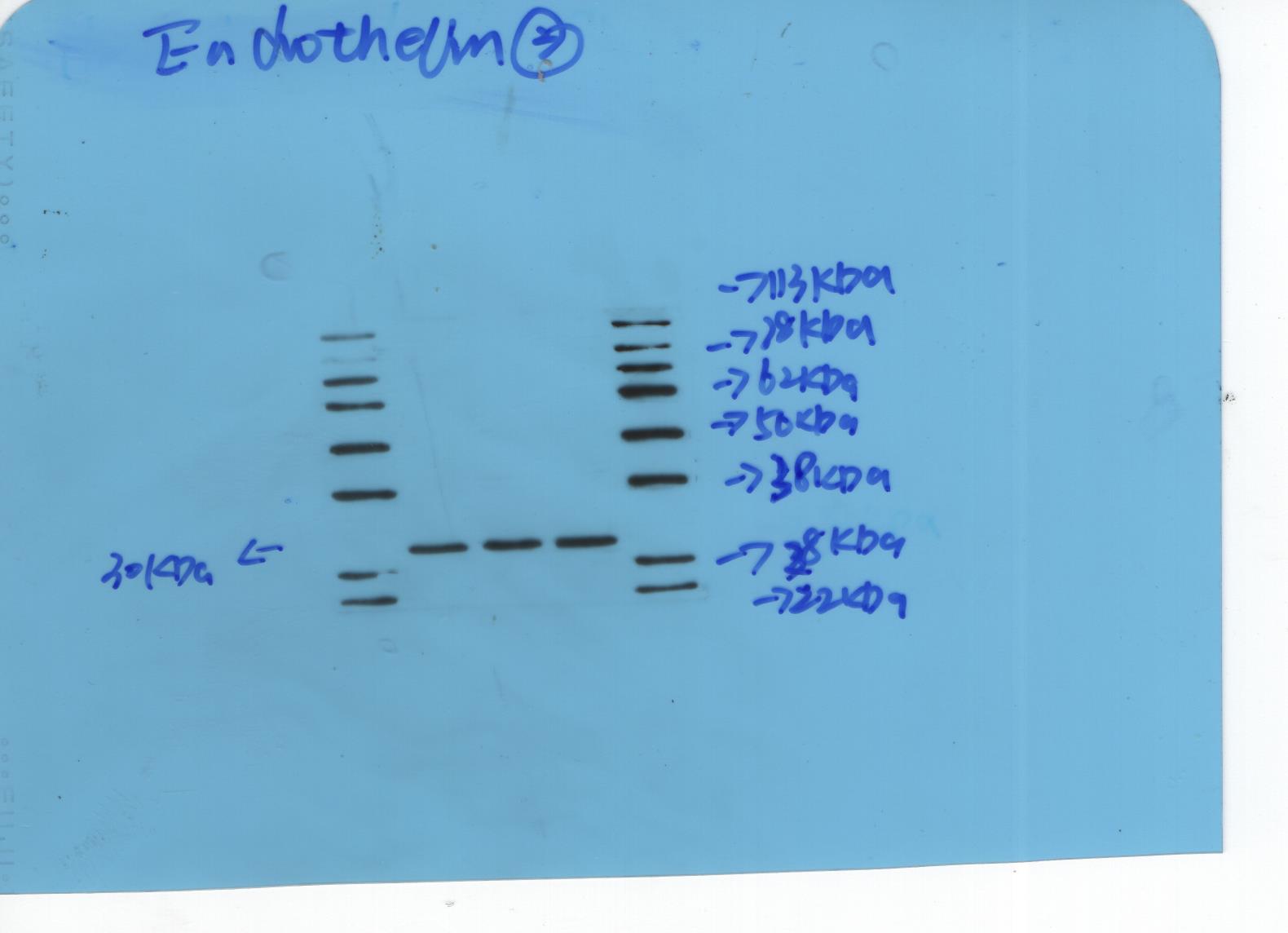


Figure 4e: The original blots/gels of endothelin, The figure in the munuscript was cropped from the second one.


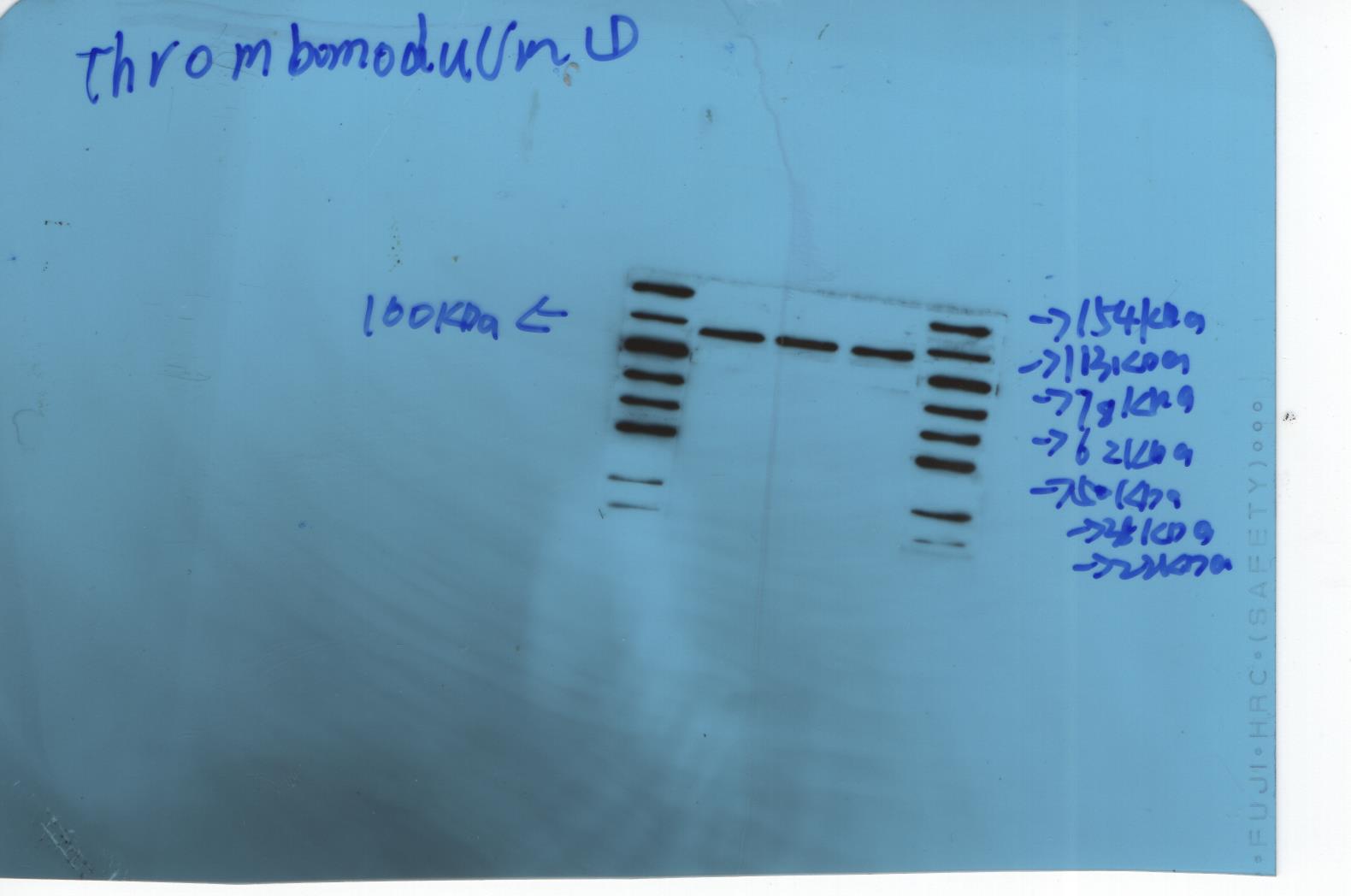

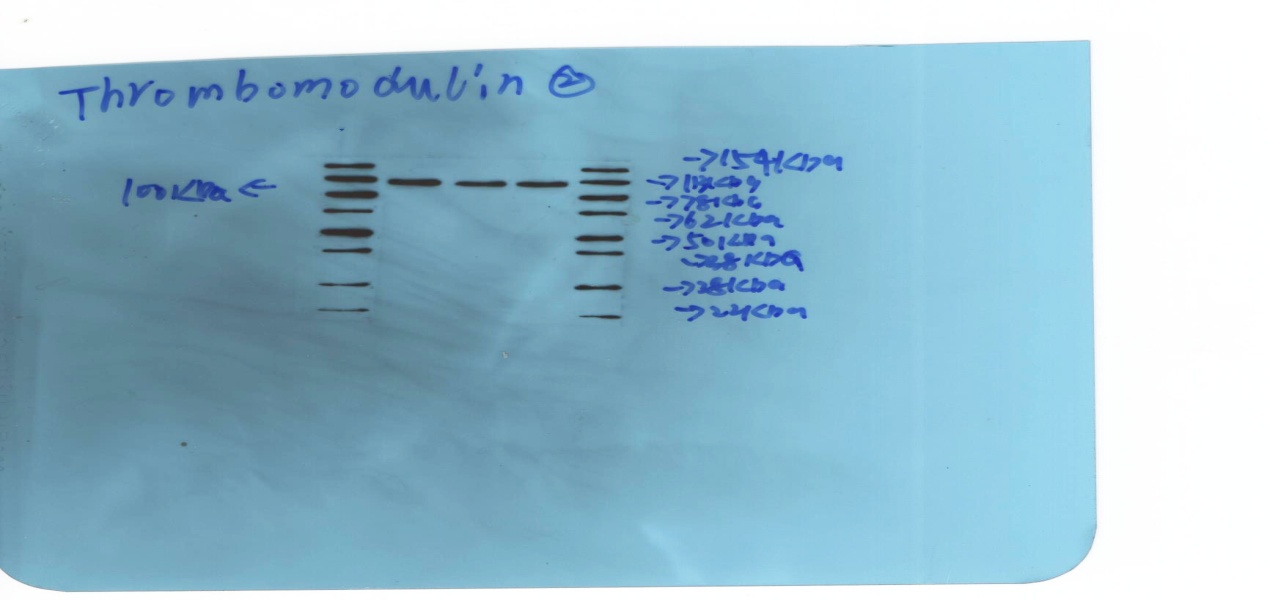

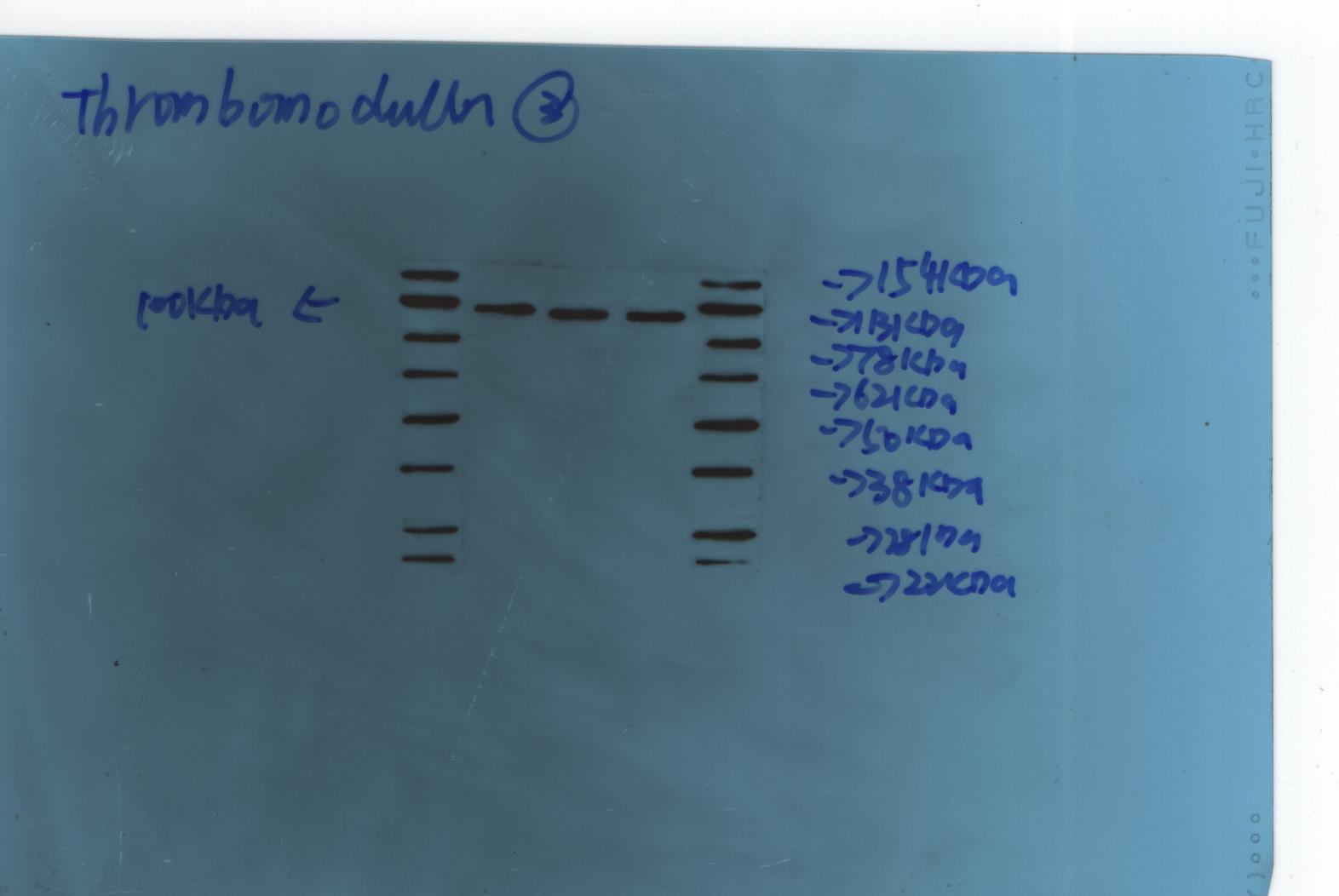


Figure 4f: The original blots/gels of thrombomodulin, The figure in the munuscript was cropped from the first one.


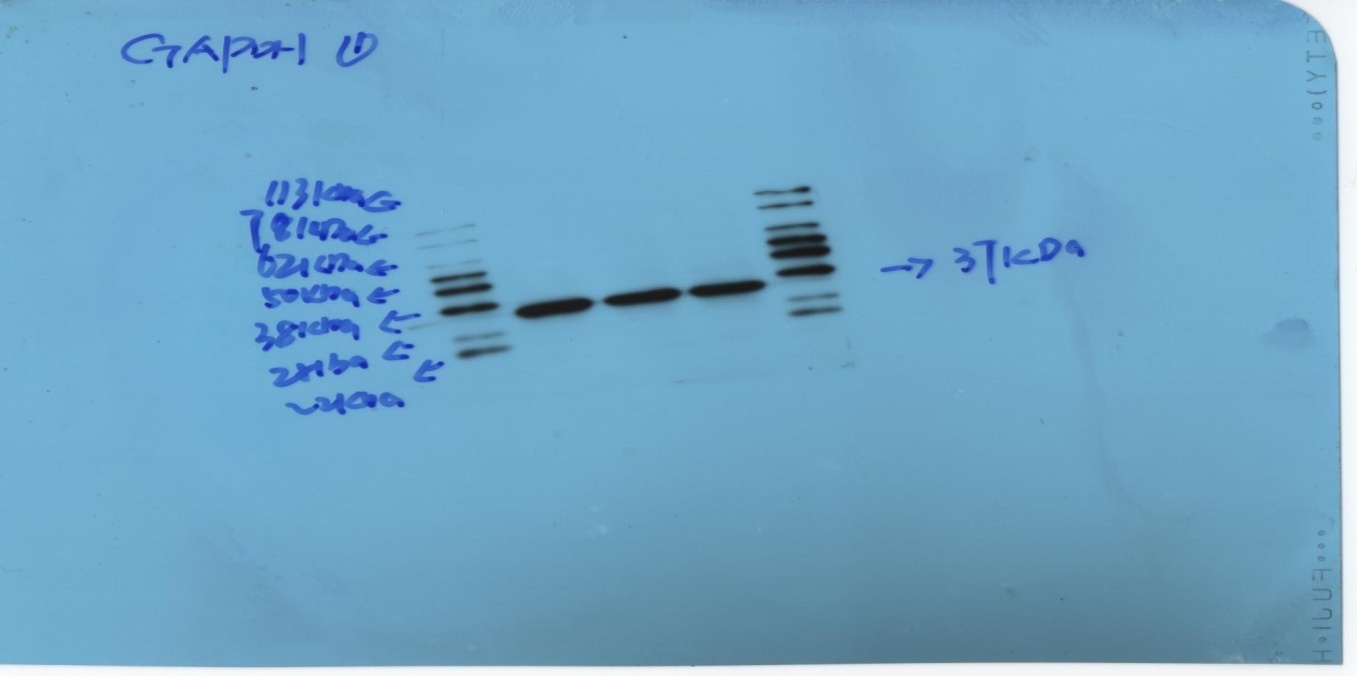

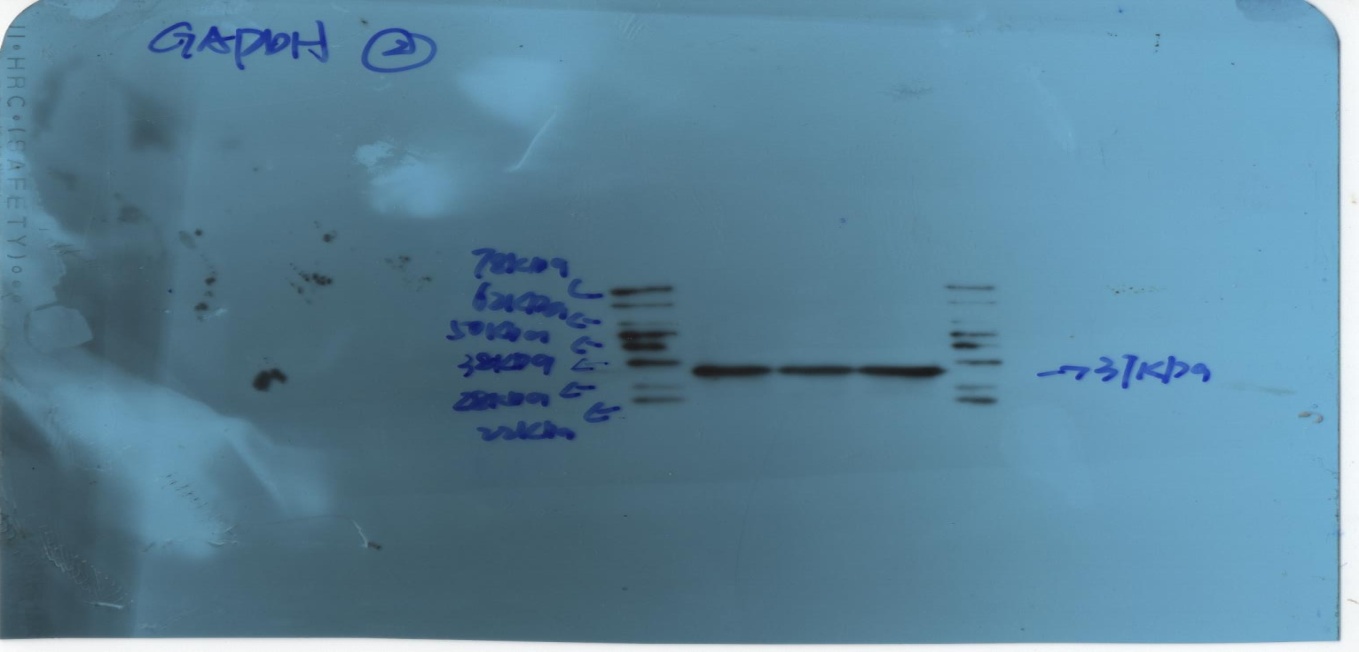


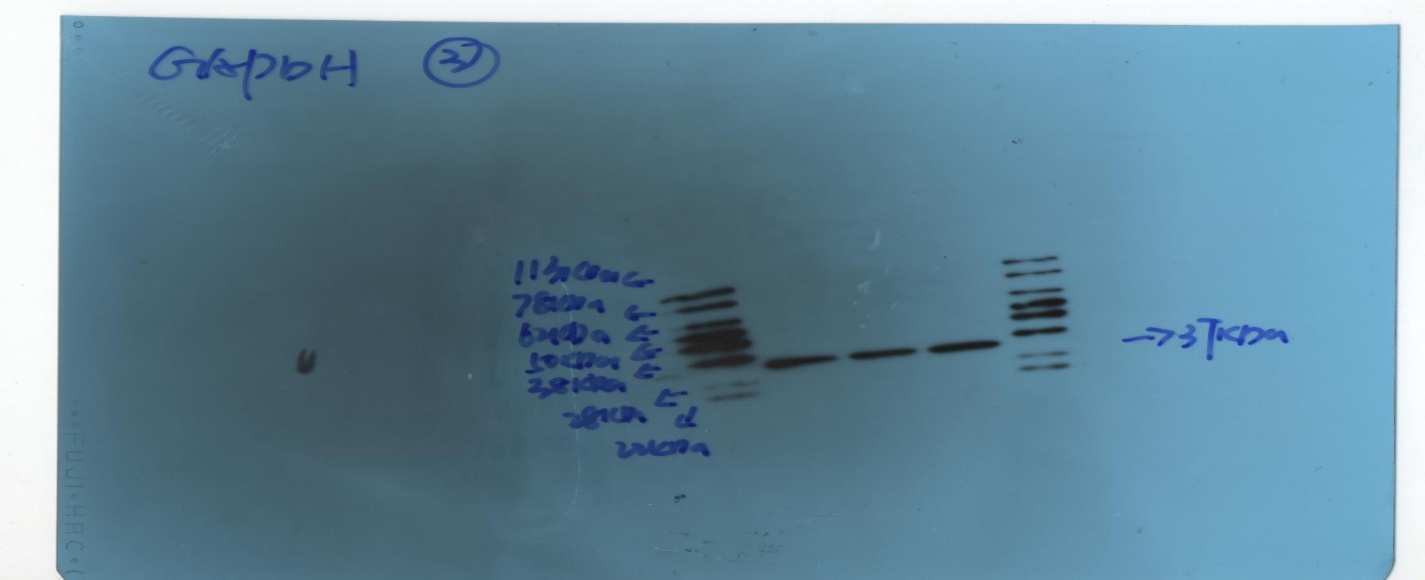


Figure 4g: The original blots/gels of GAPDH, The figure in the munuscript was cropped from the first one.


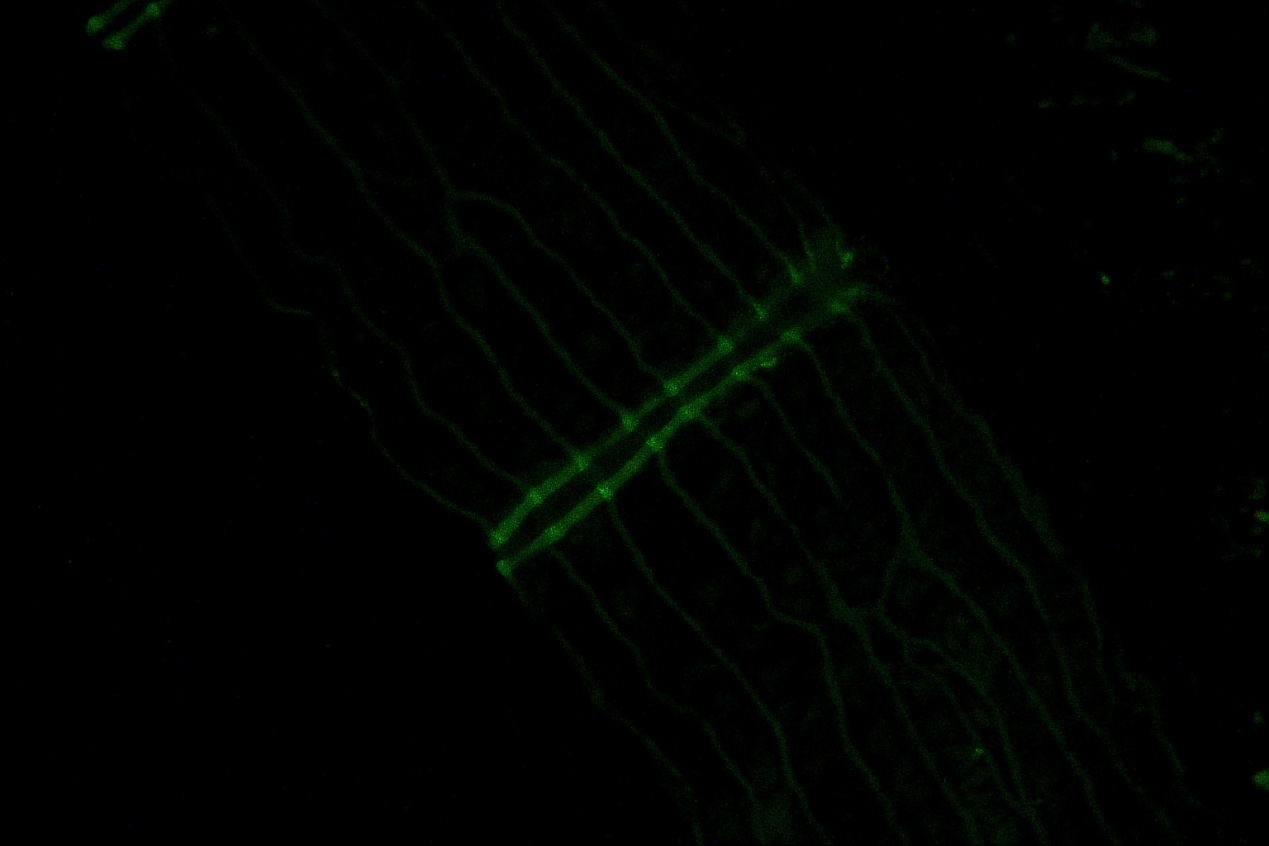


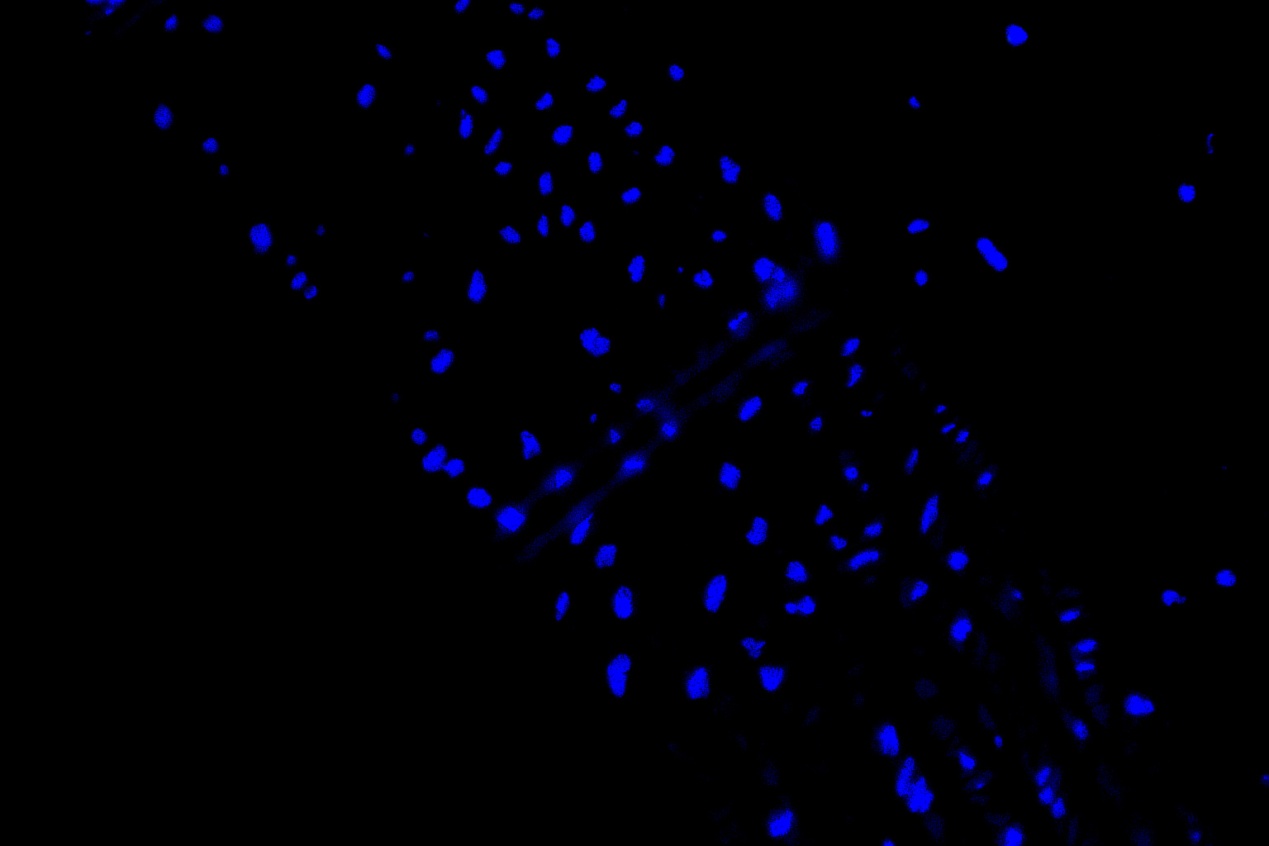


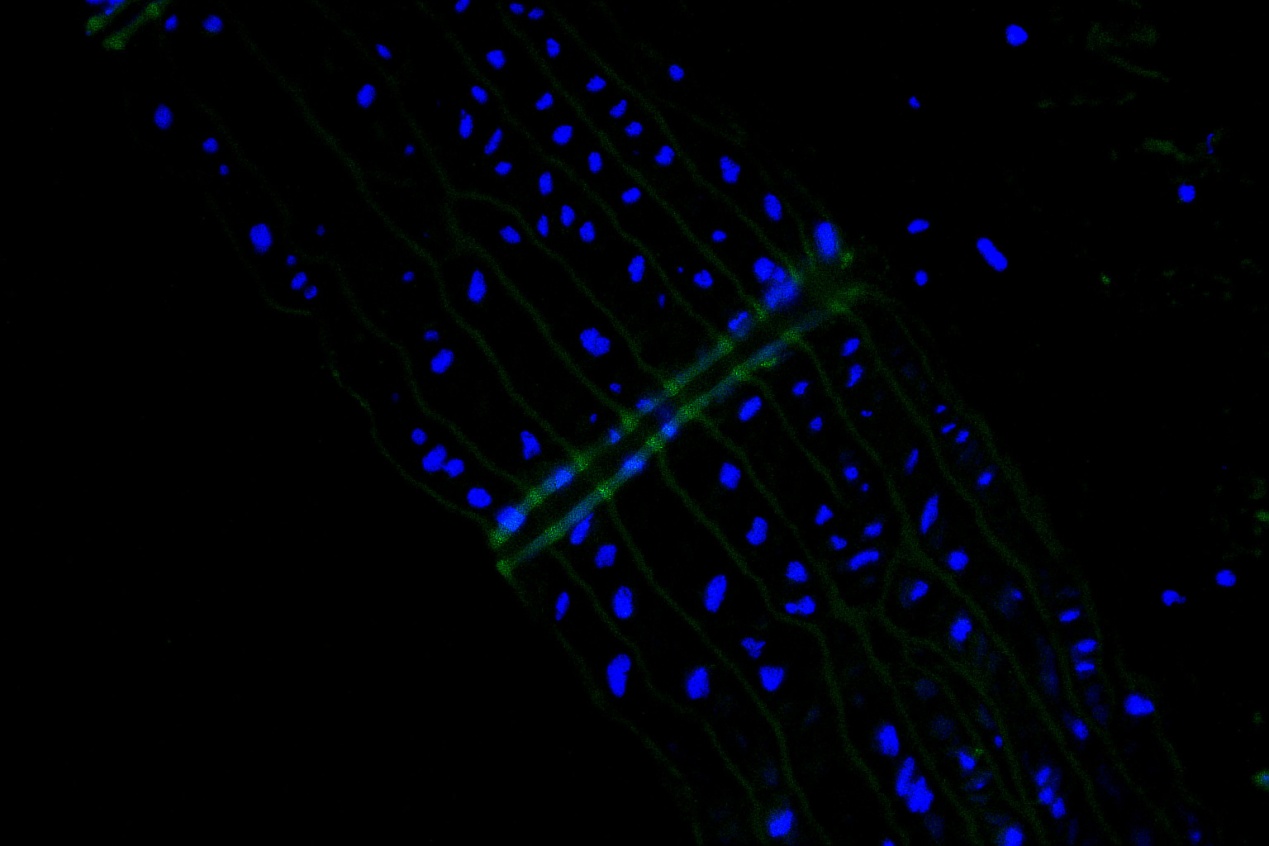


Figure 5a: The fluorescence intensity of CD40 in vascular endothelial cells in FS group.


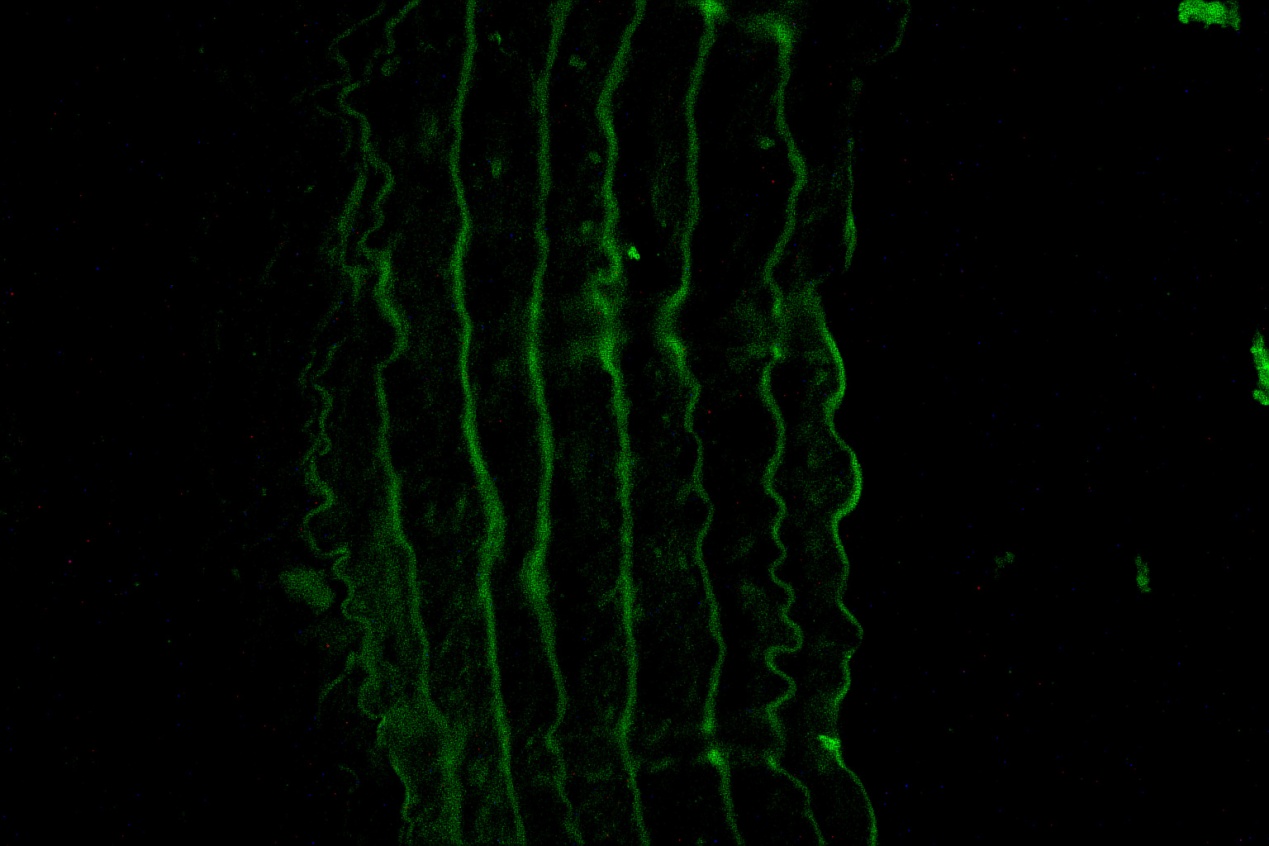


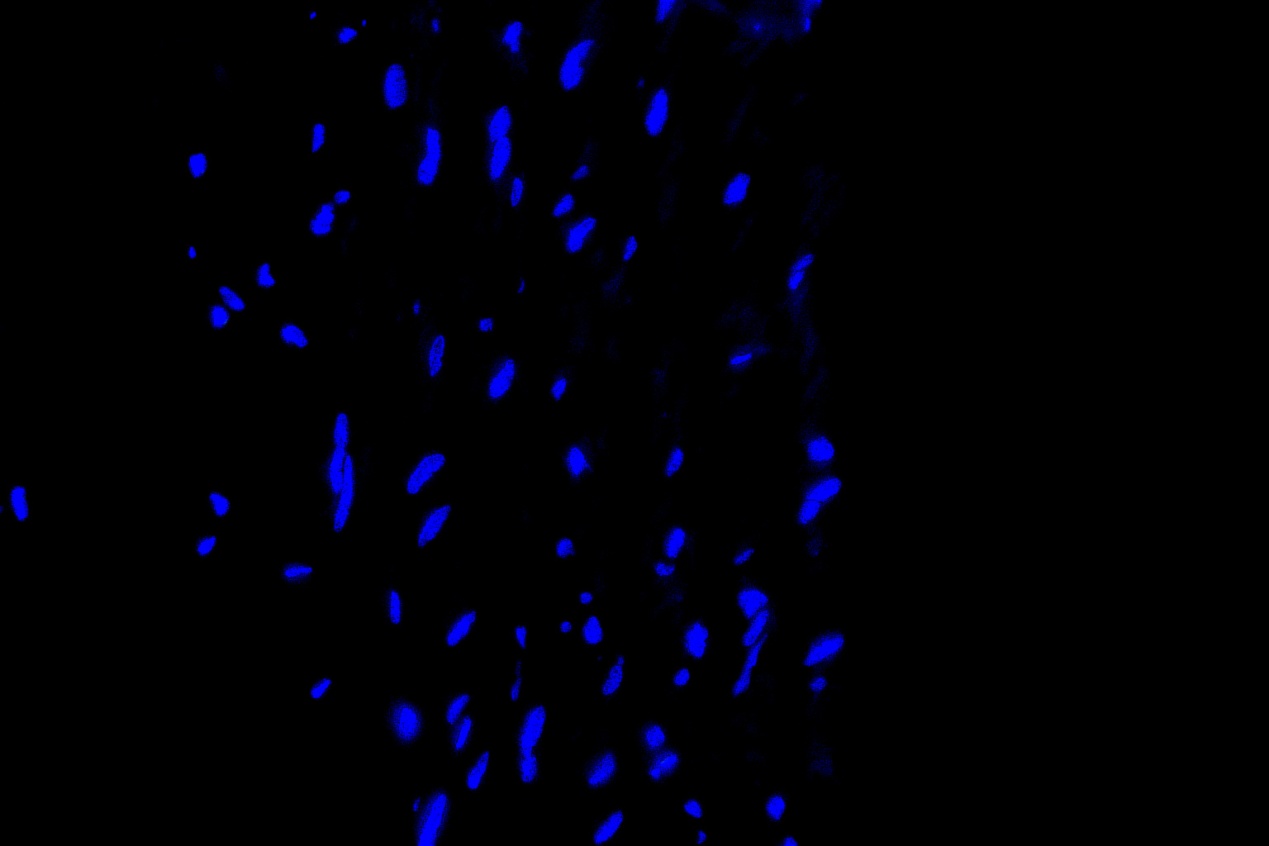


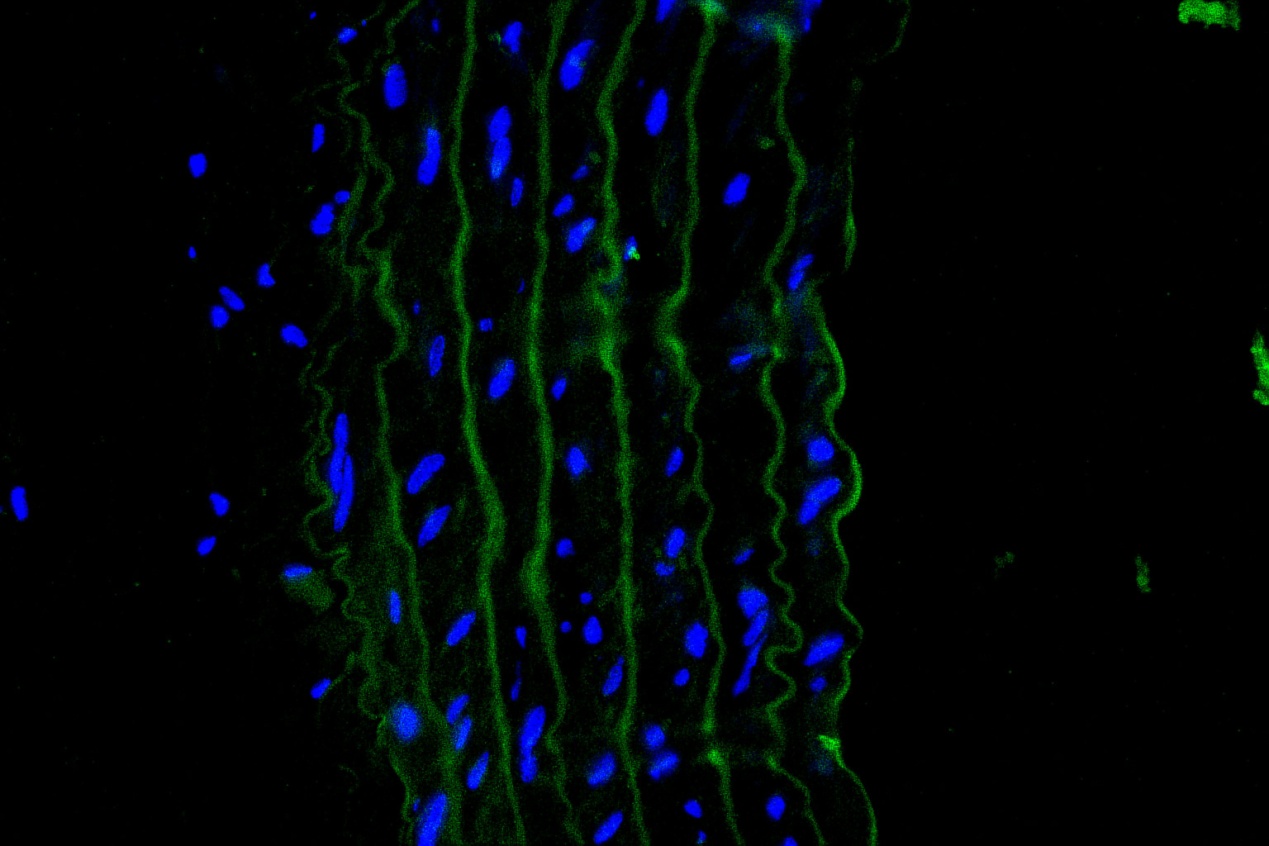


Figure 5b: The fluorescence intensity of CD40 in vascular endothelial cells in CON group.


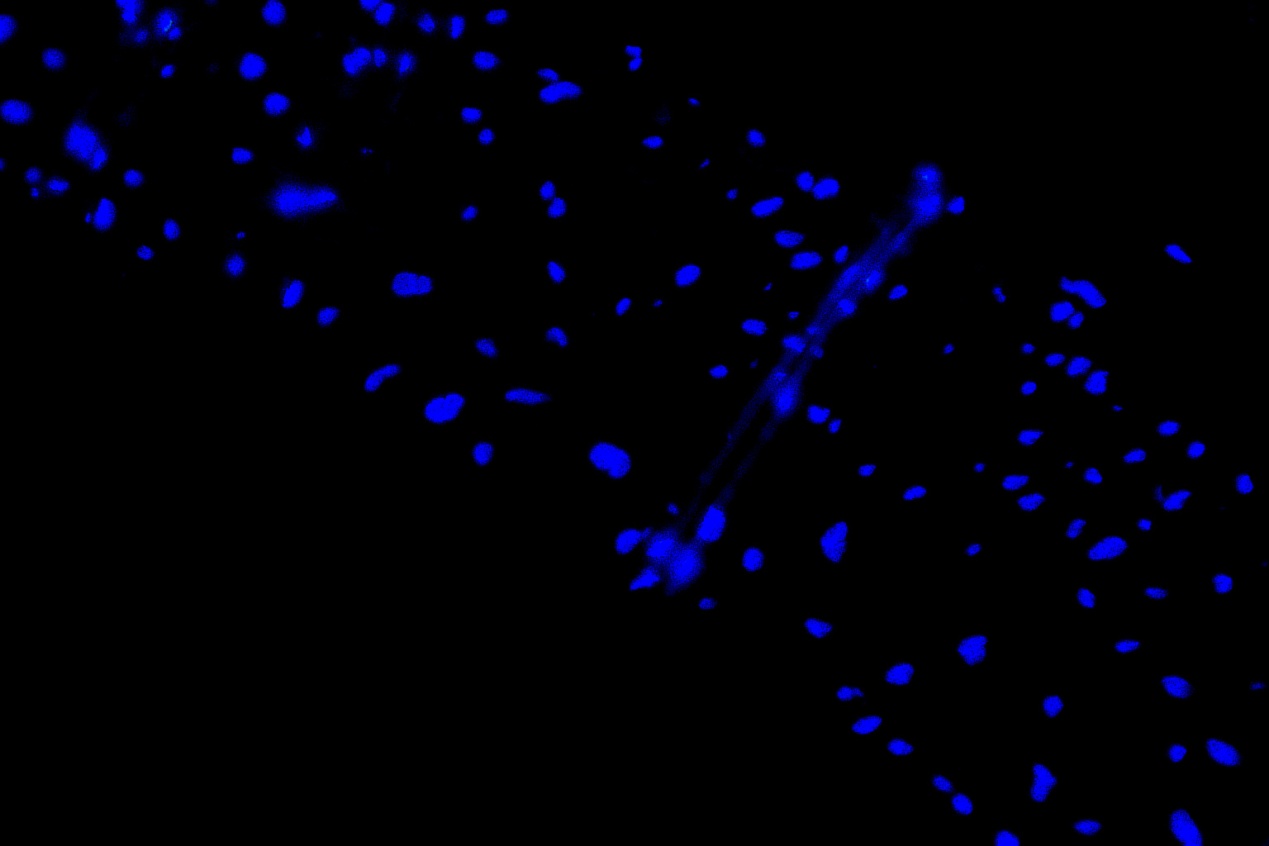


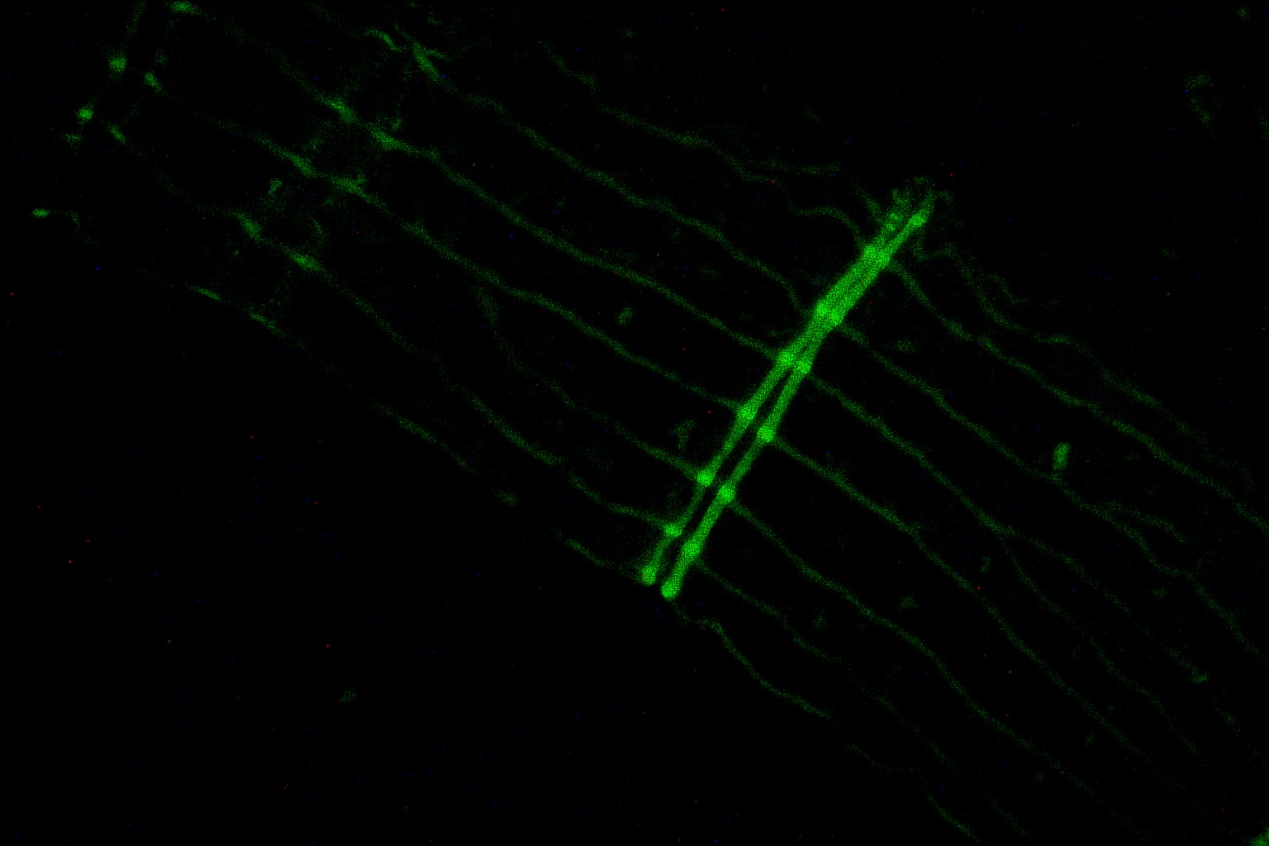


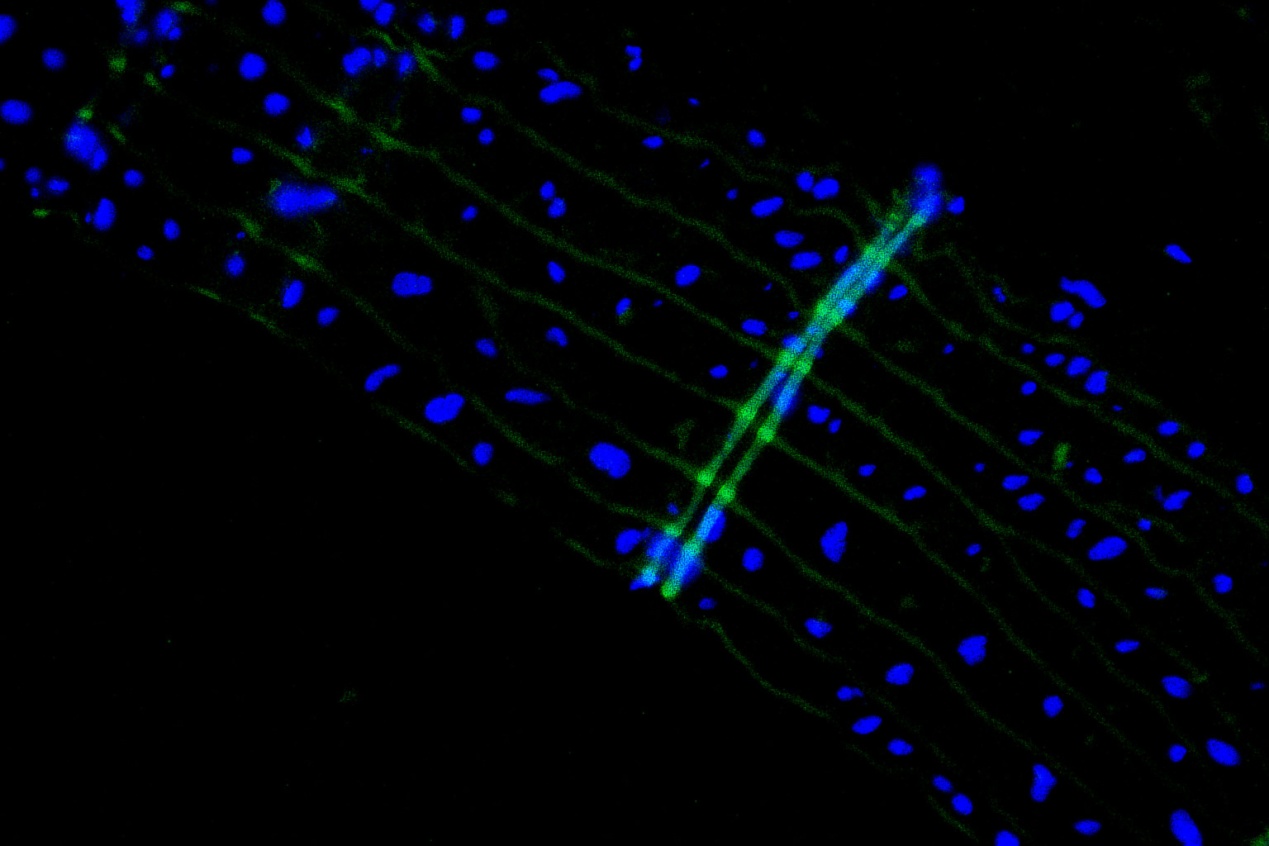


Figure 5c: The fluorescence intensity of CD40 in vascular endothelial cells in M group.
